# Ursolic acid reduces oxidative stress injury to ameliorate experimental autoimmune myocarditis by activating Nrf2/HO-1 signaling pathway

**DOI:** 10.3389/fphar.2023.1189372

**Published:** 2023-07-21

**Authors:** Yanan Fu, Tianshu Liu, Shukun He, Yichan Zhang, Yuting Tan, Ying Bai, Jiawei Shi, Wenhui Deng, Jiani Qiu, Zhen Wang, Yihan Chen, Qiaofeng Jin, Mingxing Xie, Jing Wang

**Affiliations:** ^1^ Department of Ultrasound Medicine, Union Hospital, Tongji Medical College, Huazhong University of Science and Technology, Wuhan, China; ^2^ Clinical Research Center for Medical Imaging in Hubei Province, Wuhan, China; ^3^ Hubei Province Key Laboratory of Molecular Imaging, Wuhan, China

**Keywords:** experimental autoimmune myocarditis, ursolic acid, oxidative stress, Nrf2, HO-1

## Abstract

**Background:** Oxidative stress is crucial in experimental autoimmune myocarditis (EAM)-induced inflammatory myocardial injury. Ursolic acid (UA) is an antioxidant-enriched traditional Chinese medicine formula. The present study aimed to investigate whether UA could alleviate inflammatory cardiac injury and determine the underlying mechanisms.

**Methods:** Six-week-old male BALB/c mice were randomly assigned to one of the three groups: Sham, EAM group, or UA intervention group (UA group) by gavage for 2 weeks. An EAM model was developed by subcutaneous injection of α-myosin heavy chain derived polypeptide (α-MyHC peptide) into lymph nodes on days 0 and 7. Echocardiography was used to assess cardiac function on day 21. The inflammation level in the myocardial tissue of each group was compared using hematoxylin and eosin staining (HE) of heart sections and Interleukin-6 (IL-6) immunohistochemical staining. Masson staining revealed the degree of cardiac fibrosis. Furthermore, Dihydroethidium staining, Western blot, immunohistochemistry, and enzyme-linked immunosorbent assay (ELISA) were used to determine the mechanism of cardioprotective effects of UA on EAM-induced cardiac injury, and the level of IL-6, Nrf2, and HO-1.

**Results:** In EAM mice, UA intervention significantly reduced the degree of inflammatory infiltration and myocardial fibrosis while improving cardiac function. Mechanistically, UA reduced myocardial injury by inhibiting oxidative stress (as demonstrated by a decrease of superoxide and normalization of pro- and antioxidant enzyme levels). Interestingly, UA intervention upregulated the expression of antioxidant factors such as Nrf2 and HO-1. *In vitro* experiments, specific Nrf2 inhibitors reversed the antioxidant and antiapoptotic effects of ursolic acid, which further suggested that the amelioration of EAM by UA was in a Nrf2/HO-1 pathway-dependent manner.

**Conclusion:** These findings indicate that UA is a cardioprotective traditional Chinese medicine formula that reduces EAM-induced cardiac injury by up-regulating Nrf2/HO-1 expression and suppressing oxidative stress, making it a promising therapeutic strategy for the treatment of EAM.

## Introduction

Myocarditis is an inflammation of the myocardium caused by viruses, bacteria, or parasites, as well as non-infectious causes such as autoimmunity, hypersensitivity, and cardiotoxicity (Frustaci et al., 2009; Caforio et al., 2015). It can result in sudden cardiac death or dilated cardiomyopathy (Felker et al., 2000). Currently, most myocarditis treatments are based on supportive and immunosuppressive therapy (Jensen and Marchant, 2016). However, effective therapies against myocarditis are lacking. The prevailing view indicates that oxidative stress exacerbates the autoimmune process in myocarditis (Tada and Suzuki, 2016). Moreover, long-term oxidative stress and antioxidant system suppression may result in cardiac remodeling in inflammatory cardiomyopathy (Tada and Suzuki, 2016). Oxidative stress is considered a potential therapeutic target to treat myocarditis. One of the hallmark changes in the myocarditis heart is increased oxidative stress, which leads to cardiac apoptosis. Nuclear factor erythroid-related factor 2 (Nrf2) is a transcription factor that activates under conditions of high oxidative stress and regulates many antioxidant and detoxification genes (Dinkova-Kostova et al., 2018). An imbalance between active oxidants and antioxidants causes oxidative stress. Under oxidative conditions, Nrf2 is released upon dissociation from kelch like ECH associated protein 1(Keap1) and translocated to the nucleus (Kobayashi et al., 2006). In the nucleus, it binds to antioxidant response elements (AREs), the promoter regions of its target genes. All of these genes, including Heme oxygenase-1 (HO-1), NAD(P)H quinone dehydrogenase 1 (NQO1), and Glutathione peroxidase (GSH-Px), have AREs in their promoter regions.

Traditional Chinese medicine has long been uniquely effective in treating inflammatory diseases. Ursolic acid (UA) is a pentacyclic triterpenoid compound found in many natural plants, such as apple peels, herbal medicines, and other edible plants (Cui et al., 2006; Seeram, 2008; Tannock, 2011). UA has numerous biological properties, including anti-tumor, anti-oxidation, anti-inflammation, anti-fibrosis, and anti-atherosclerosis, and can be used to treat various diseases (Wang et al., 2011; Silva et al., 2016). Some of these effects of UA are determined by its potent antioxidant properties (López-Hortas et al., 2018). Moreover, mounting evidence suggests the versatile roles of UA in the cardiovascular system (Saravanan, Pugalendi, 2006). Previous studies have confirmed that UA is a cardioprotective compound due to its antioxidant properties (Liobikas et al., 2011). However, whether UA can improve experimental autoimmune myocarditis (EAM) is unknown.

Several works have demonstrated the modulation of Nrf2 by UA as one of the main mechanisms behind their beneficial effects in pathological models like cigarette smoke-induced emphysema (Lin et al., 2017) and renal tubular epithelial cell damage induced by calcium oxalate monohydrate. Nevertheless, the regulation of this antioxidant pathway in cardiac pathology remains to be further investigated (Jia et al., 2021). The Nrf2 pathway has been shown to play a critical role in EAM as it reduces DNA damage induced by oxidative stress in EAM mice (Jaén et al., 2020). However, it is unknown whether UA can alleviate inflammation and oxidative injury in EAM through Nrf2/HO-1 signaling pathway.

In the present study, an EAM murine model was developed to investigate the regulatory role of UA in anti-inflammatory and cardiac antioxidant pathways. The present study investigated whether UA can reduce EAM-induced oxidative stress injury and whether this effect is mediated through Nrf2 and HO-1 signaling pathways. The present study provides new insights for developing therapeutic targets to improve the prognosis and treatment of myocarditis patients.

## Materials and methods

### Experimental animals and EAM model

Male BALB/c mice aged 6–7 weeks (weighing 18–20 g) were obtained from Beijing Vital River Laboratory Animal Technology Co., LTD. (China) and housed at 25°C ± 2°C with free access to water and food on a 12 h light/dark cycle. The Animal Ethics Committee of Tongji Medical College, Huazhong University of Science and Technology (China) approved all experimental procedures that complied with the National Institutes of Health Guidelines on the Use of Laboratory Animals (Approval code: S3136; The validity of approval is from 01/01/2022-12/31/2024).

All mice were anesthetized using 1.5% isoflurane before the procedure to minimize animal stress. Mice were immunized with a cardiac-specific peptide (α-MyHC peptide: Ac-RSLKLMATLFSTYASADR-OH) purchased from GL Biochem Ltd (China). The peptide was dissolved in saline and emulsified in a 1:1 ratio with Complete Freund’s Adjuvant (Sigma, United States) (n = 30). On day 0, 200 μg peptide in 0.2 mL of the emulsion was injected subcutaneously into one side of the axillary and inguinal lymph node region. On day 7, the same dose was injected subcutaneously into the opposite side. Sham group (n = 12) was injected with a physiological saline solution instead of myosin. Mice immunized with cardiac peptide were randomly divided into UA treatment (n = 16) and PBS (n = 14) groups. From day 7–21, mice were given intragastric gavage of UA ((U6753, Sigma, United States), 100 mg/kg) (UA intervention group) or PBS containing 7.5% DMSO (D2650, Sigma, United States) (sham group and peptide–immunized mice without UA). Therefore, three experimental groups were formed: 1) a sham group, 2) an EAM group, and 3) an EAM + UA group. On day 21, all mice underwent echocardiography. The blood was then obtained through cardiac puncture. Body weight (BW) and tibial length (TL) were measured. Finally, hearts were removed from the chest and subjected to subsequent experiments.

### Transthoracic echocardiography

On day 21, the cardiac function was assessed using an echocardiographic imaging system (Vevo 1,100, Canada) with a transducer MS400 (30 MHz). The mice were anesthetized with 1.5% isoflurane, and two-dimensional echocardiographic views of the mid-ventricular short axis at the papillary muscle tips below the mitral valve were obtained. The Vevo 1,100 software calculated the left ventricular ejection fraction (EF) and fractional shortening (FS).

### Measurement of cytokines by enzyme-linked immunosorbent assay (ELISA)

Tissue (10 mg) was homogenized in PBS on ice for ELISA. The samples were centrifuged for 10 min at 5,000 × g to remove any insoluble material. Plasma samples were collected by cardiac puncture. Interleukin-6 (IL-6), cardiac troponin I (cTnI), and brain natriuretic peptide (BNP) levels were measured using ELISA kits (mlbio, China) according to the manufacturer’s instructions.

### Malondialdehyde (MDA) assay

MDA was measured using the MDA assay kit (Beyotime, China) as directed by the manufacturer. Plasma (100 µL) or homogenized cells (1 × 10^6^) samples were added to 200 µL MDA Lysis Buffer. The samples were incubated at 100°C for 15 min before being cooled in an ice bath to room temperature. The samples were centrifuged at 1,000 *g* for 10 min at room temperature to remove any insoluble material. Then, 200 µL of supernatant was pipetted into a 96-well plate, and the absorbance was measured at 532 nm (A_532_).

### Cell culture and *in vitro* experimental protocols

H9c2 cells were obtained from the cell bank of the Chinese Academy of Sciences (China). The cells were cultured in dulbecco’s modified eagle medium (DMEM) (high glucose) medium (Gibco, United States) supplemented with 10% fetal bovine serum (FBS) (Gibco, United States)and 1% of penicillin-streptomycin (Gibco, United States) in a humidified atmosphere of 5% CO_2_ at 37°C. Cells were maintained in a proliferative state (i.e., undifferentiated state). To induce inflammation, cells were rendered quiescent by serum starvation for 12 h prior to stimulation with rIL-6 (25 ng/mL) for 1 h (Shi et al., 2020). In order to study the antioxidant effect of ursolic acid, 10 μM, 20 μM, 30 μM, 40 μM, and 50 µM of UA was added to the cells culture 4 h after the addition of rIL-6. For Nrf2 inhibitor experiments, cultures were co-incubated by 30 µM UA and 5 µM ML385 for 4 h (HY-100523, MCE, United States) (Chen et al., 2021). The cells were split into four groups: 1) Sham group; 2) IL-6 group; 3) UA group (IL-6 + UA); 4) ML385 group (IL-6 + UA + ML385).

### Cell viability assay

Cell viability was determined using the Cell Counting Kit-8 (CCK-8) (Biosharp, China) assay following the manufacturer’s instructions. The cells were seeded at a density of 5,000 cells/well in the 96-well plates. After 24 h, cells were treated with different concentrations of UA. The CCK-8 solution was added to each well, and the cells were incubated at 37°C for another 2 h. The absorbance was measured at 450 nm (A_450_). Each sample was replicated thrice. The cells that stained positive for CCK-8 solution was thought to be viable. The results are presented as a percentage compared with sham group.

### Histopathology and immunohistochemistry

Myocardial tissues from left ventricle (LV) of mice at the papillary muscle level were harvested, fixed in fresh 4% paraformaldehyde, embedded in paraffin, and serially sectioned (5–6 μm). Dewaxed and hydrated heart sections were stained with hematoxylin and eosin staining (HE) for general morphology or with Masson trichrome stain kit for fibrosis detection. The blue stained area (collagen deposition) was quantified and normalized to the total section area using ImageJ (1.8.0, National Institutes of Health) software to determine cardiac fibrosis.

### Immunofluorescence staining

The paraffin sections were baked for 1 h in a 65°C oven. The sections were dewaxed and antigen repaired before incubating in 3% H_2_O_2_ for 10 min to remove endogenous enzymes. After 1 h of blocking with goat serum at room temperature, heart slides were incubated overnight at 4°C with the following primary antibodies: α-Smooth Muscle Actin (α-SMA) (1:100) (#7817, Abcam, United Kingdom), IL-6 (1:50) (#290735, Abcam, United Kingdom). Samples were incubated with secondary antibodies combined with Alexa Fluor 488 and 555 (Beyotime, China) for 1 h at 1:500, followed by 4′,6-diamidino-2-phenylindole (DAPI) (Beyotime, China) for 15 min at room temperature. Finally, the sections were washed with PBS 1x and covered with coverslips.

### TUNEL assay

Myocardial apoptosis was determined by terminal deoxynucleotidyl transferase dUTP nick-end labeling (TUNEL) staining by TUNEL Apoptosis Assay Kit with fluorescein (Beyotime, China) following the manufacturer’s instructions. Green fluorescein staining was used to identify apoptotic nuclei, while DAPI stained total nuclei. The apoptotic index was expressed as the percentage of apoptotic nuclei to total nuclei.

### Western blot analysis

Cardiac tissues or cells were homogenized in ripa lysis buffer (include protease and phosphatase Inhibitor) with a sonicator on ice, and the total protein concentrations were measured using the BCA Protein Assay Kit (Beyotime, China). As previously reported, cytosolic and nucleic proteins were separated for analysis of Nrf2 release. Depending on the molecular weight of target proteins, equal amounts of protein lysates (20–40 μg/lane) were subjected to 10%–12% sodium dodecyl sulfate-polyacrylamide gel electrophoresis (SDS-PAGE) and immunoblotted to PVDF membranes (MerckMillipore, Germany). After blocking with 5% skim milk for 1 h at room temperature, the membranes were incubated at 4°C overnight with respective primary antibodies against Nrf2 (1:1,000) (16,396, proteintech, China), HO-1 (1:1,000, ab52947), LaminB1 (1:1,000, ab133741), glyceraldehyde-3-phosphate dehydrogenase (GAPDH) (1:2000, CST#5174), β-actin (1:1,000, ab8226) and washed. The protein bands were visualized using enhanced chemiluminescence (Pierce, United States) after incubation with the corresponding secondary HRP-conjugated antibodies. Densitometry values were determined using ImageJ software (1.8.0, National Institutes of Health).

### Real-time quantitative polymerase chain reaction (RT q-PCR)

TRIzol Reagent (Invitrogen, United States) was used to isolate total RNA from flash-frozen myocardial tissues or H9c2 cells. cDNA was synthesized using a reverse transcription reagent kit (Takara Biotechnology, Japan) and then amplified in the Bio-rad CXF CONNECT Detector system using the SYBR^®^ Premix Ex TaqTM Perfect Real Time Kit (Takara Biotechnology, Japan). Relative expression was computed using the CT method. Table 1 lists the SYBR Green real-time PCR primers for mouse gene expression.

**TABLE 1:** RT q-PCR primer sequences.

	Primer sequences (5′-3′)
GAPDH	Forward: 5′-ACT​CCA​CTC​ACG​GCA​AAT​TCA-3′
	Reverse: 5′-GGC​CTC​ACC​CCA​TTT​GAT​G-3′
IL-6	Forward: 5′-ACA​ACC​ACG​GCC​TTC​CCT​ACT-3′
	Reverse: 5′-CTC​ATT​TCC​ACG​ATT​TCC​CAG​A-3′
Col1a1	Forward: 5′-AAT​GGC​ACG​GCT​GTG​TGC​GA-3′
	Reverse: 5′-AGC​ACT​CGC​CCT​CCC​GTC​TT-3′
Col3a1	Forward: 5′-CCT​GGC​TCA​AAT​GGC​TCA​C-3′
	Reverse: 5′- GAC​CTC​GTG​TTC​CGG​GTA​T-3′

### Molecular docking

The crystal structure of Nrf2 protein (PDB ID: 7O7B) has been obtained from the RCSB protein data bank. For protein preparation, the natural ligand was extracted and the resulting crystal structure was then freed of the water molecules and added the polar hydrogen atoms. The 3D structure of ursolic acid has been obtained from pubchme database. This structure was optimized and polar hydrogen atoms are added. Subsequently, the docking procedure was conducted by using Autodock software and its conformation was optimized by using a genetic algorithm based on the principle of minimizing docking energy.

### Statistical analysis

The data were analyzed with GraphPad Prism software (version 8.0 for Windows, San Diego, CA, United States). All data are normally distributed. The multiple-group comparisons were performed using one-way analysis of variance (ANOVA), followed by Tukey’s multiple comparison test for homogeneity of variance and Games-Howell’s multiple comparisons test for heterogeneity of varianc. All values were presented as mean ± SD. The differences were considered statistically significant with a *p*-value <0.05.

## Results

### Ursolic acid ameliorated myocardial damage in EAM mice

**FIGURE 1:**
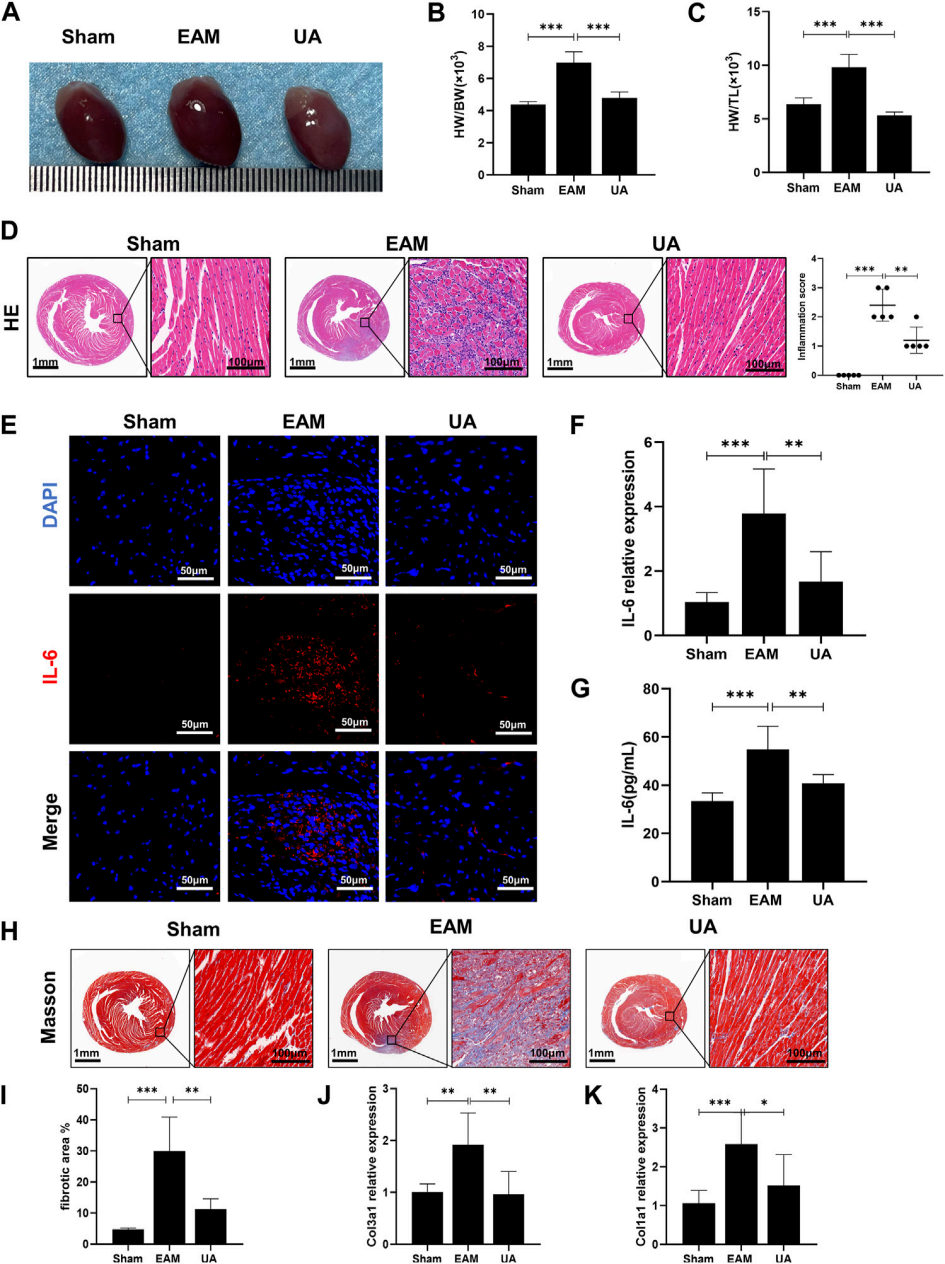
UA mitigated EAM-induced cardiac inflammation and fibrosis. **(A)** Heart images representing each experimental group. **(B)** Heart weight to body weight ratio (HW/BW). **(C)** Heart weight to tibial length ratio (HW/TL) from all experimental groups. **(D)** Representative histological images of immune cell infiltration in cardiac tissue from the indicated groups and the corresponding quantification (n = 5). Scale bars: left 1mm, inset 100 μm. **(E)** Representative immunofluorescence images of cardiac tissue stained red with IL-6 and blue with DAPI. Scale bars: 50 μm. **(F)** RT-qPCR detected the mRNA levels of IL-6 in cardiac tissue. **(G)** ELISA detected IL-6 levels in cardiac tissue from all experimental groups at day 21. **(H, I)** Representative Masson staining images indicating collagen fiber deposition in blue and the corresponding quantification (n = 8). Scale bars: left 1 mm, inset 100 μm. **(J, K)** Col1a1 and Col3a1 mRNA expression levels in cardiac tissue from the indicated groups at day 21 (n = 9). (Data were shown as the mean ± SD, ordinary one-way analysis of variance, Tukey’s multiple comparisons test and Games-Howell’s multiple comparisons test; ns = not significant, *p < 0.05, **p < 0.01, ***p < 0.001).

The ratios of heart weight/body weight (HW/BW) and heart weight/tibia length (HW/TL) were elevated and heart was enlarged in EAM mice compared to sham group, which was alleviated by UA treatment (Figures 1A–C). H&E-stained EAM heart sections revealed a high inflammation score, which was reduced by UA treatment (Figure 1D). Immunofluorescence staining was used to characterize the immune cells in the infiltrated area. Compared with sham group, IL-6 was significantly increased in EAM group, while other inflammatory factors IL-1β and TNF-α had no significant difference (Figure 1E; Supplementary Figure S1A,B). In addition, UA could partially normalize IL-6 mRNA expression and the secretion level of IL-6 in EAM mouse heart tissues (Figures 1F,G).

In EAM model, heart sections stained with Masson’s trichrome to detect collagen fiber deposition revealed some peripheral fibrotic areas in EAM mice that were reduced by UA administration (Figures 1H,I). Immunofluorescence staining with α-SMA, a fibroblast activation marker, was also increased in EAM animals, whereas it was deceased in UA-treated mice (Supplementary Figure S1C). The fibrosis analysis was supplemented by estimating mRNA levels of mediators involved in the development of cardiac fibrosis. In the present study, EAM mice had higher levels of collagen 1 (Col1a1) and 3 (Col3a1) cardiac mRNA that significantly reduced in UA-treated EAM mice (Figure 1J,K). Ursolic acid intervention alleviated myocardial inflammation and fibrosis in mice with EAM.

### Ursolic acid treatment mitigated EAM-Induced systolic dysfunction

**FIGURE 2:**
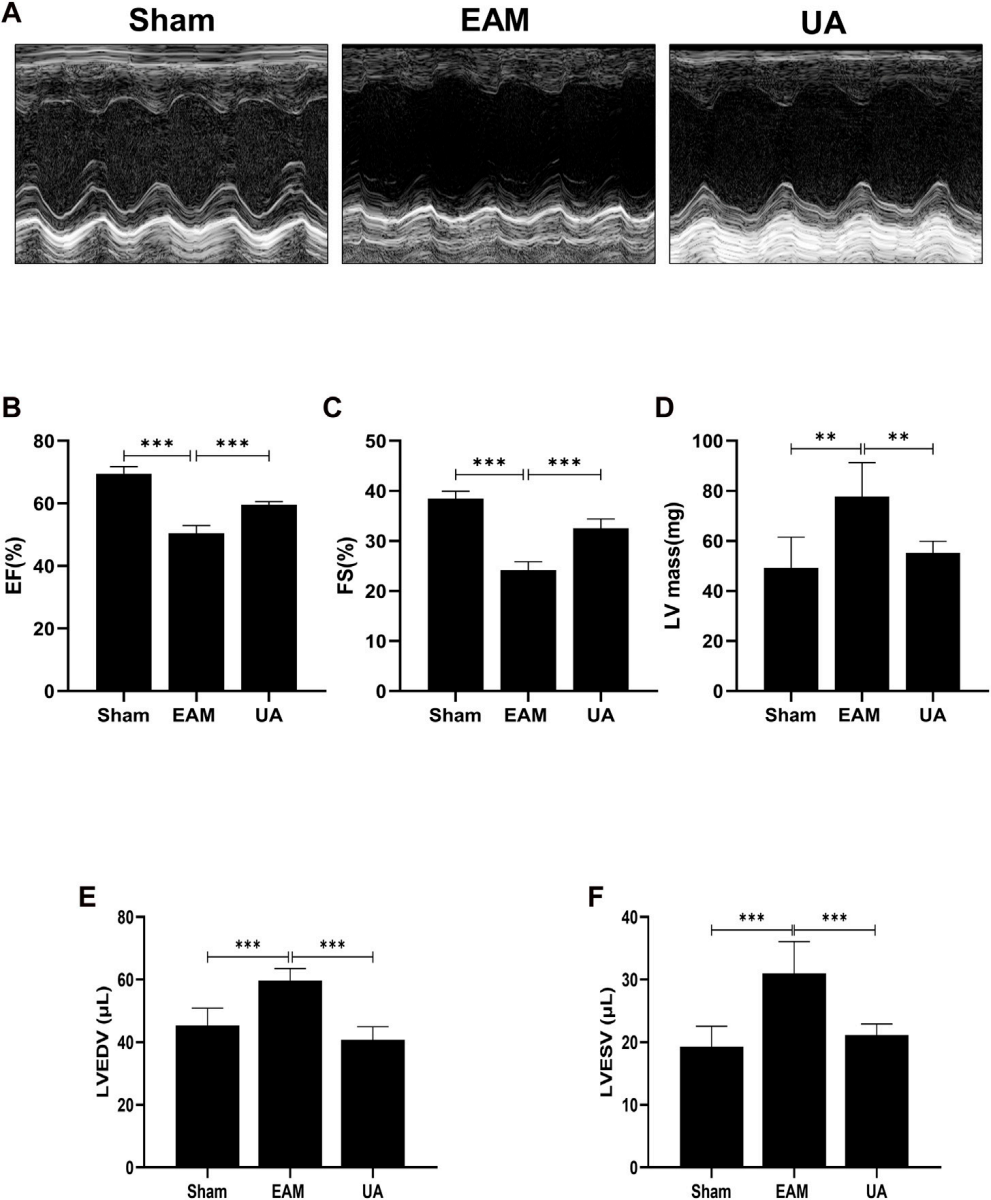
UA improved EAM-induced left ventricular dysfunction. **(A)** Representative echocardiography M-mode images from the indicated groups on day 21. **(B, C)** Statistical analysis of ejection fractions (EF) and fractional shorting (FS) in indicated groups. **(D, E, F)** Left ventricular function-related ultrasonic parameters in each group (n = 6). (Data were shown as the mean ± SD, ordinary one-way analysis of variance, Tukey’s multiple comparisons test; ns = not significant, *p < 0.05, **p < 0.01, ***p < 0.001).

On day 21, echocardiography was performed to assess cardiac function in each group (Figure 2; Table 2). Figure 2A depicts the representative M-mode echocardiograms for each group. Cardiac systolic dysfunction, as indicated by left ventricular EF and FS, decreased significantly in the EAM group but not in the UA-treated group (Figures 2B,C). When compared to sham group, the EAM group had substantially higher levels of left ventricular end-diastolic volume (LVEDV), left ventricular end-systolic volume (LVESV), and left ventricular mass (LV Mass), which was mitigated in the UA-treated group (Figures 2D–F). Other factors, such as heart rate (HR), did not differ between the groups (Table 2). Ursolic acid reduced left ventricular dysfunction induced by EAM.

**TABLE 2:** Cardiac function parameters obtained from echocardiography.

	Sham	EAM	UA
Heart rate (bpm)	401 ± 86	410 ± 21	382 ± 26
Ejection fraction%	69.5 ± 2	50 ± 2.5***	59.5 ± 1^###^
Fractional shortening%	38 ± 1.5	24 ± 2***	32 ± 1.5^###^
LVEDV (μL)	46 ± 6	60 ± 4***	41 ± 4^###^
LVESV (μL)	19 ± 3	31 ± 5***	21 ± 2^###^
LV mass (mg)	49 ± 12	78 ± 14**	55 ± 4.5^##^

Data are presented as mean ± SD, ordinary one-way analysis of variance, Tukey’s multiple comparisons test.

* *p* < 0.05.

** *p* < 0.01.

*** *p* < 0.001 vs. Control.

# *p* < 0.05.

## *p* < 0.01.

### *p* < 0.001 vs EAM. LVEDV, Left ventricular end-diastolic volume; LVESV, left ventricular end-systolic volume.

### Ursolic acid treatment reduced cardiac apoptosis

**FIGURE 3:**
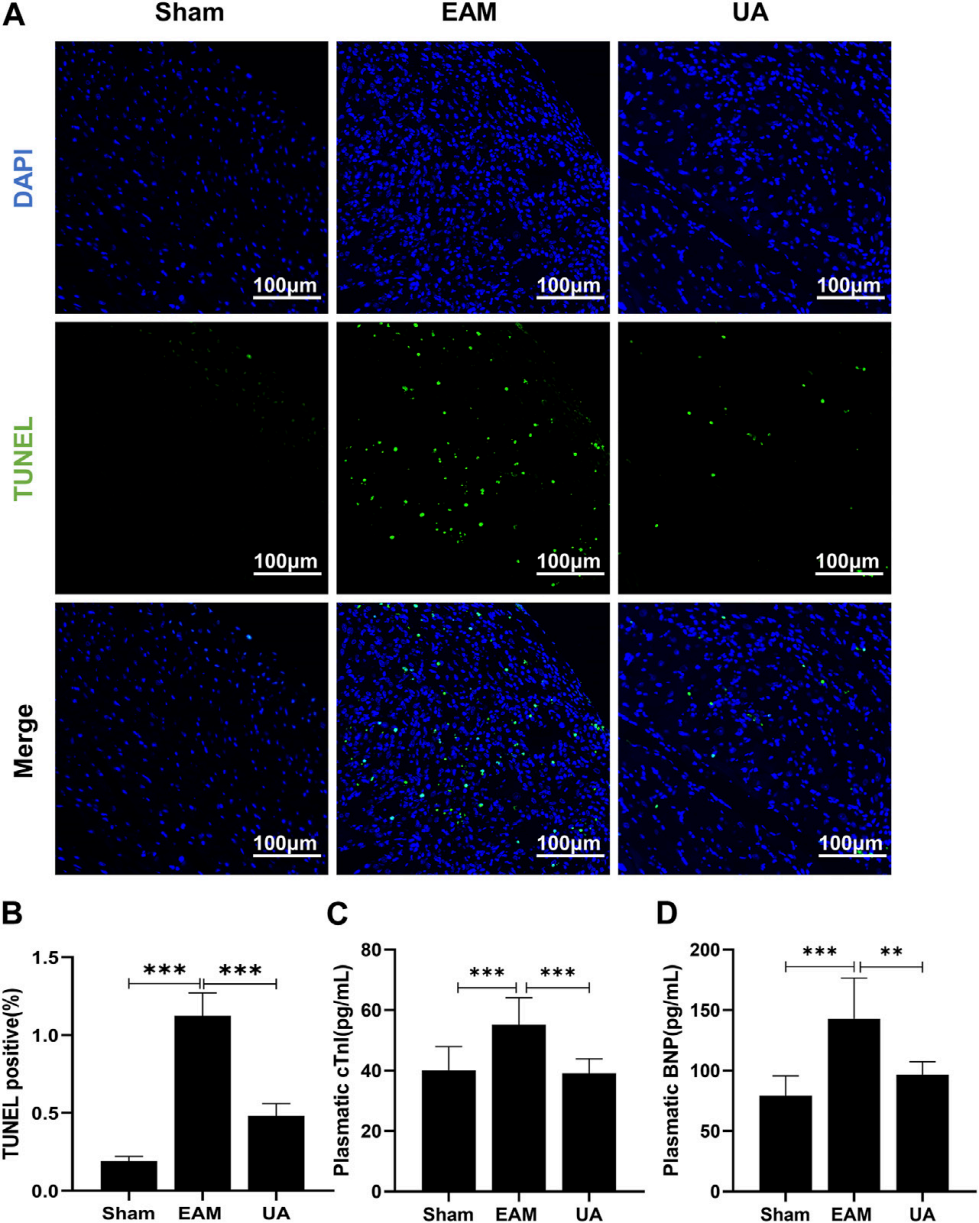
UA reduced EAM-induced cardiomyocyte apoptosis. **(A)** TUNLE staining images of cardiac tissue stained with TUNEL-positive nuclei in green and DAPI in blue. Scale bars: 100 μm. **(B)** Quantitative analysis of apoptotic index among the indicated groups (n = 5). **(C, D)** cTnI and BNP levels detected by ELISA in cardiac tissue from the indicated groups (n = 9). (Data were shown as the mean ± SD, ordinary one-way analysis of variance, Tukey’s multiple comparisons test and Games-Howell’s multiple comparisons test; ns = not significant, *p < 0.05, **p < 0.01, ***p < 0.001).

Cardiac dysfunction has been associated with increased cardiomyocytes death, which eventually debilitates the contractile capacity of the heart. Findings indicated that the proportion of TUNEL-positive nuclei increased significantly in EAM group (Figures 3A,B). In addition, increased plasmatic levels of the cardiac damage marker cTnI (Figure 3C) and BNP (Figure 3D) were found in EAM mice. However, both parameters were significantly lower in UA-treated EAM mice.

### Effects of ursolic acid in the cardiac oxidative stress development: Nrf2 signaling pathway

**FIGURE 4:**
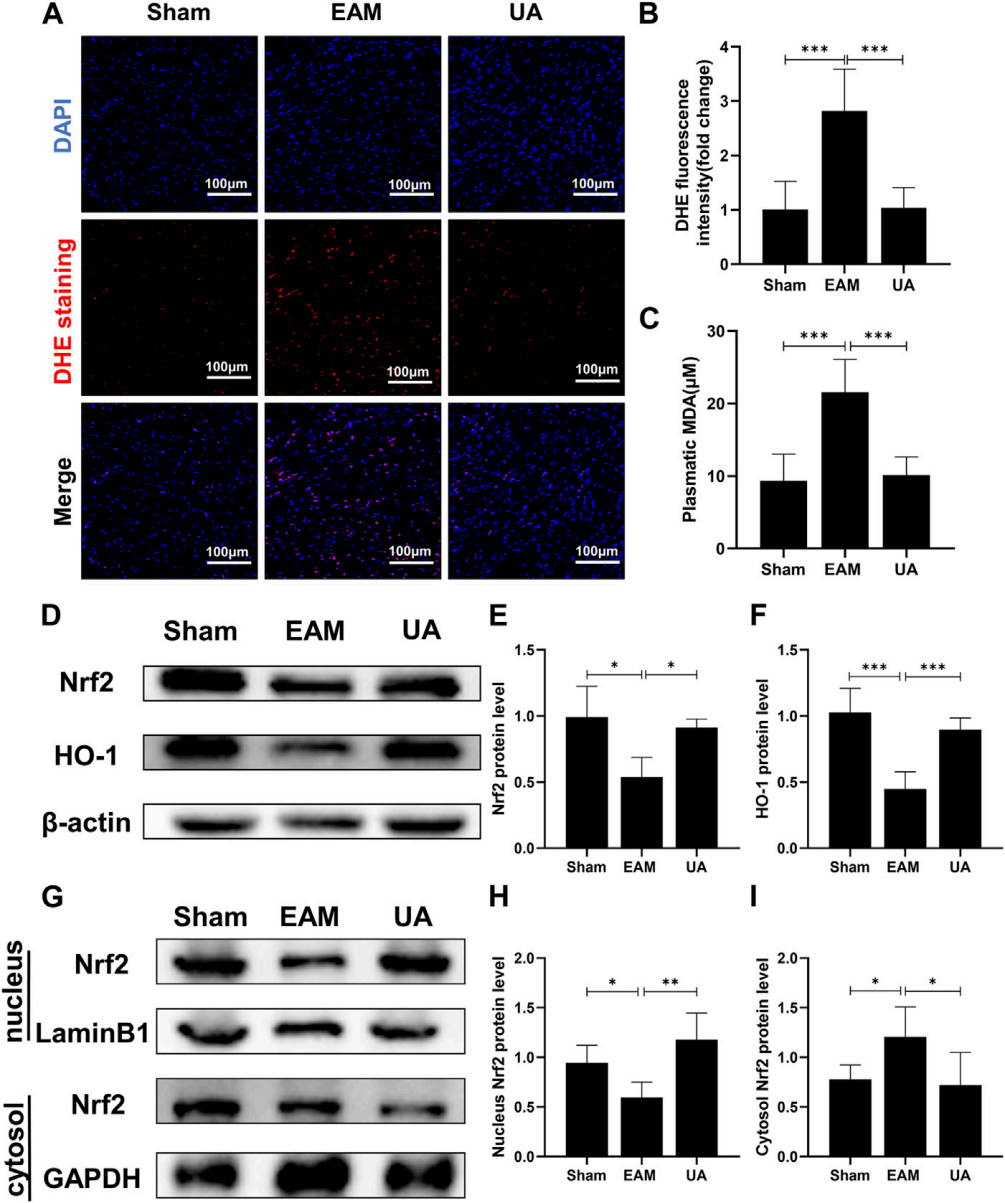
UA reduced the EAM-induced cardiac oxidative stress by regulating Nrf2 levels. **(A)** Heart images of DHE staining in the indicated groups. Scale bars: 100 μm. **(B)** The fluorescence intensity of DHE staining was measured and expressed as a fold change (n = 3). **(C)** An MDA assay measured serum MDA levels in each group. **(D,E, F)** The total cellular Nrf2 and HO-1 protein expression levels in each group were measured with Western blot and the representative quantification (n = 6). **(G,H, I)** Protein expressions of Nrf2 in cytoplasmic and nuclear cell lysates in mouse hearts of the different groups. (Data were shown as the mean ± SD, ordinary one-way analysis of variance, Tukey’s multiple comparisons test and Games-Howell’s multiple comparisons test; ns = not significant, *p < 0.05, **p < 0.01, ***p < 0.001).

Hearts from EAM animals were also exposed to elevated levels of oxidative stress that overwhelms the capacity of antioxidant systems to cope with this severe inflammatory scenario. Therefore, cytosolic reactive oxygen species (ROS) sensor DHE was used to characterize intracellular ROS, and MDA content was measured to assess lipid peroxidation end products. The findings revealed that EAM mice had higher ROS levels and lipid peroxidation levels than sham group, while UA-treated mice had lower levels (Figure 4A–C).

Nrf2 is an important transcriptional factor and the Nrf2/HO-1 axis is an important antioxidative stress pathway in the body. The present study identified that EAM reduced Nrf2/HO-1 protein expression of mice while UA could restore it (Figure 4D–F). Furthermore, UA treatment activated total cellular and nuclear Nrf2 in mouse heart but not cytoplasmic Nrf2 (Figure 4G–I). These findings demonstrate that UA’s cardio-protective effect is partially due to the induction of the antioxidant response via the Nrf2 pathway activation.

### Ursolic acid upregulated Nrf2/HO-1 signaling pathway *in Vitro*

**FIGURE 5:**
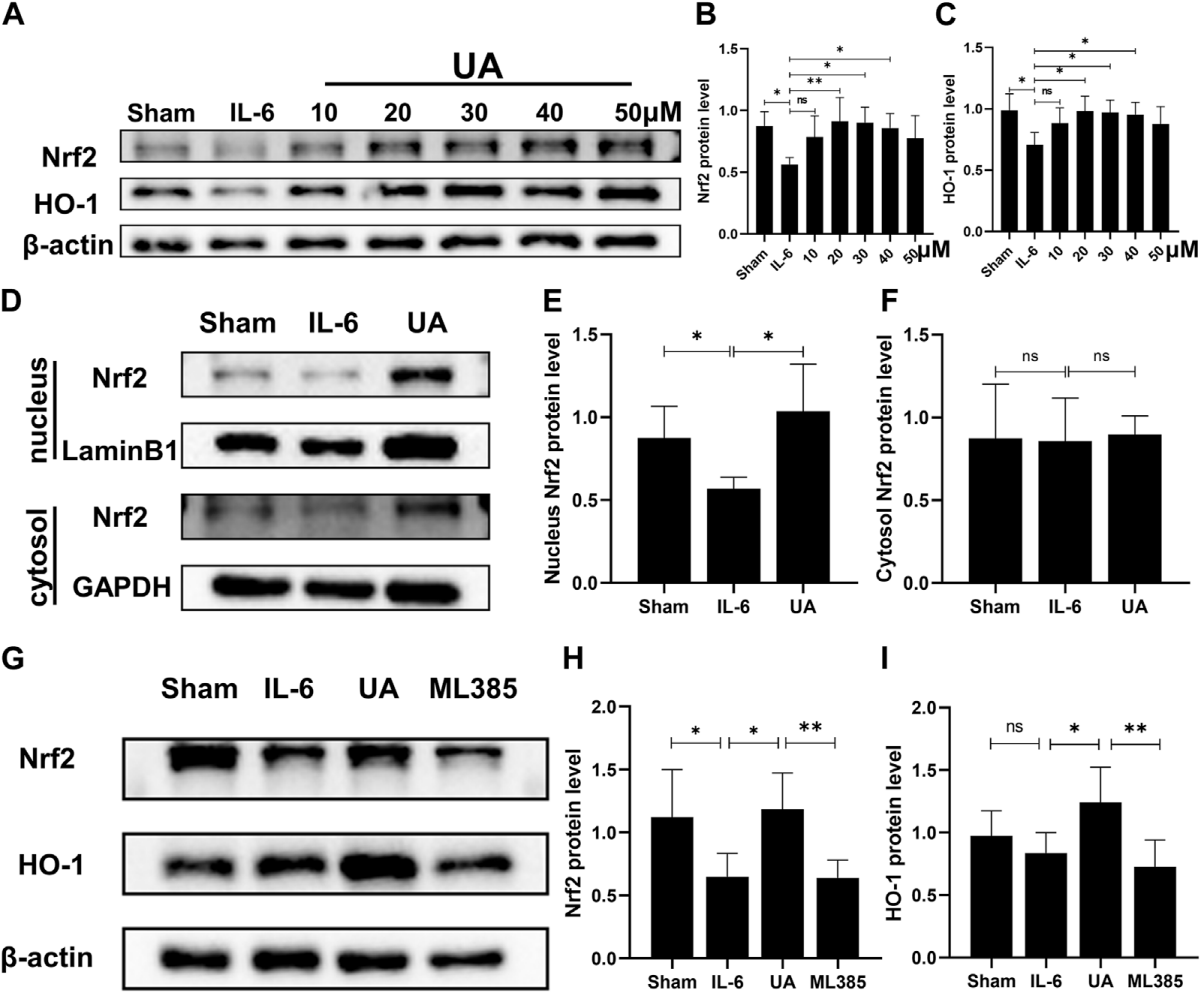
UA upregulated Nrf2/HO-1 signaling pathway *in vitro*. **(A, B, C)** Representative Western blot images and quantification of Nrf2 and HO-1 protein levels of H9c2 cells treated with UA at various concentrations (n = 6). **(D, E, F)** Protein expression of Nrf2 in cytoplasmic and nuclear cell lysate from different groups of H9c2 cells (UA = 30 µM). **(G, H, I)** The Nrf2 and HO-1 protein expression in H9c2 cells were measured with Western blot and the representative quantification (n = 6). (Data were shown as the mean ± SD, ordinary one-way analysis of variance, Tukey’s multiple comparisons test and Games-Howell’s multiple comparisons test; ns = not significant, *p < 0.05, **p < 0.01, ***p < 0.001).

To further confirm the molecular mechanism of UA in the inflammatory injury of cardiomyocytes, H9c2 cell was used *in vitro* experiments. To investigate the role of Nrf2 in the UA-mediated beneficial effects, we performed a set of experiments in which we exposed IL-6-stimulated cells to UA (10 μM, 20 μM, 30 μM, 40 μM, and 50 µM) for 4 h and quantified Nrf2 total protein levels in H9c2 cell (Figure 5A–C). To investigate the toxicity of UA to cells, we examined cell survival at various concentration points after 24 h of UA intervention (Supplementary Figure S1D). Findings revealed that cell survival was significantly reduced after 24 h of treatment with 50 µM UA. However, the cell survival rate did not change significantly when the concentration of UA less than 50 µM. In conclusion, 30 µM UA was selected for further experiments. The inflammatory cells stimulated by IL-6 were separated nucleoprotein from cytoplasmic protein by nuclear protein extraction kit. In IL-6-stimulated cells, the expression level of Nrf2 in nucleoprotein increased significantly after UA treatment. Meanwhile, there were no significant differences in the expression of Nrf2 in the cytoplasm between the groups (Figure 5D–F).

To further testify that the effects of UA were Nrf2/HO-1 pathway-dependent, specifc Nrf2 inhibitor ML385 was utilized *in vitro*. Protein expression of both Nrf2 and HO-1 was reduced after co-incubation of ML385 with UA in cardiomyocytes (Figure 5G–I; Supplementary Figure S1E–G).

### ML385 counteracted the anti-apoptosis and antioxidant effects of ursolic acid

**FIGURE 6:**
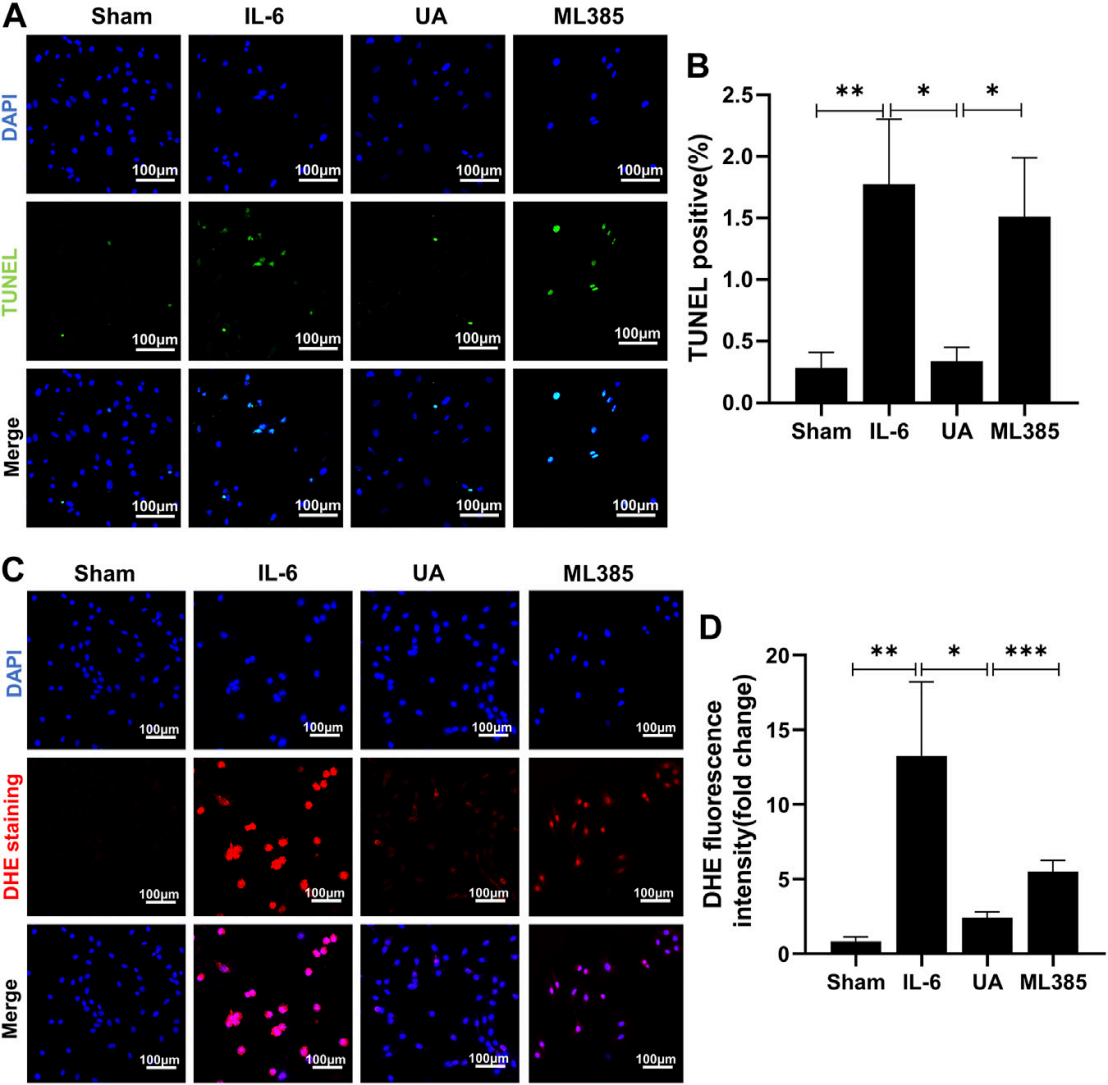
ML385 counteracted the anti-apoptosis and antioxidant effects of Ursolic Acid. **(A)** TUNEL staining images of H9c2 cells stained with TUNEL-positive nuclei in green and DAPI in blue. Scale bars: 100 μm. **(B)** Quantitative analysis of apoptotic index among the indicated groups (n = 5). **(C)** Representative images of DHE staining in H9c2 cells. Scale bars: 100 μm. **(D)** Fluorescence intensity of DHE staining was measured, and the results were expressed as a fold change (n = 6). (Data were shown as the mean ± SD, ordinary one-way analysis of variance, Tukey’s multiple comparisons test and Games-Howell’s multiple comparisons test; ns = not significant, *p < 0.05, **p < 0.01, ***p < 0.001).

Inflammation increased the percentage of apoptotic cells and intracellular ROS. UA attenuated inflammation-induced apoptosis and ROS accumulation, which were nearly abrogated by administration of ML385 (Figure 6). It suggested that the anti-apoptotic and antioxidant effects of UA were mainly achieved by activating Nrf2.

### Docking analysis

**FIGURE 7:**
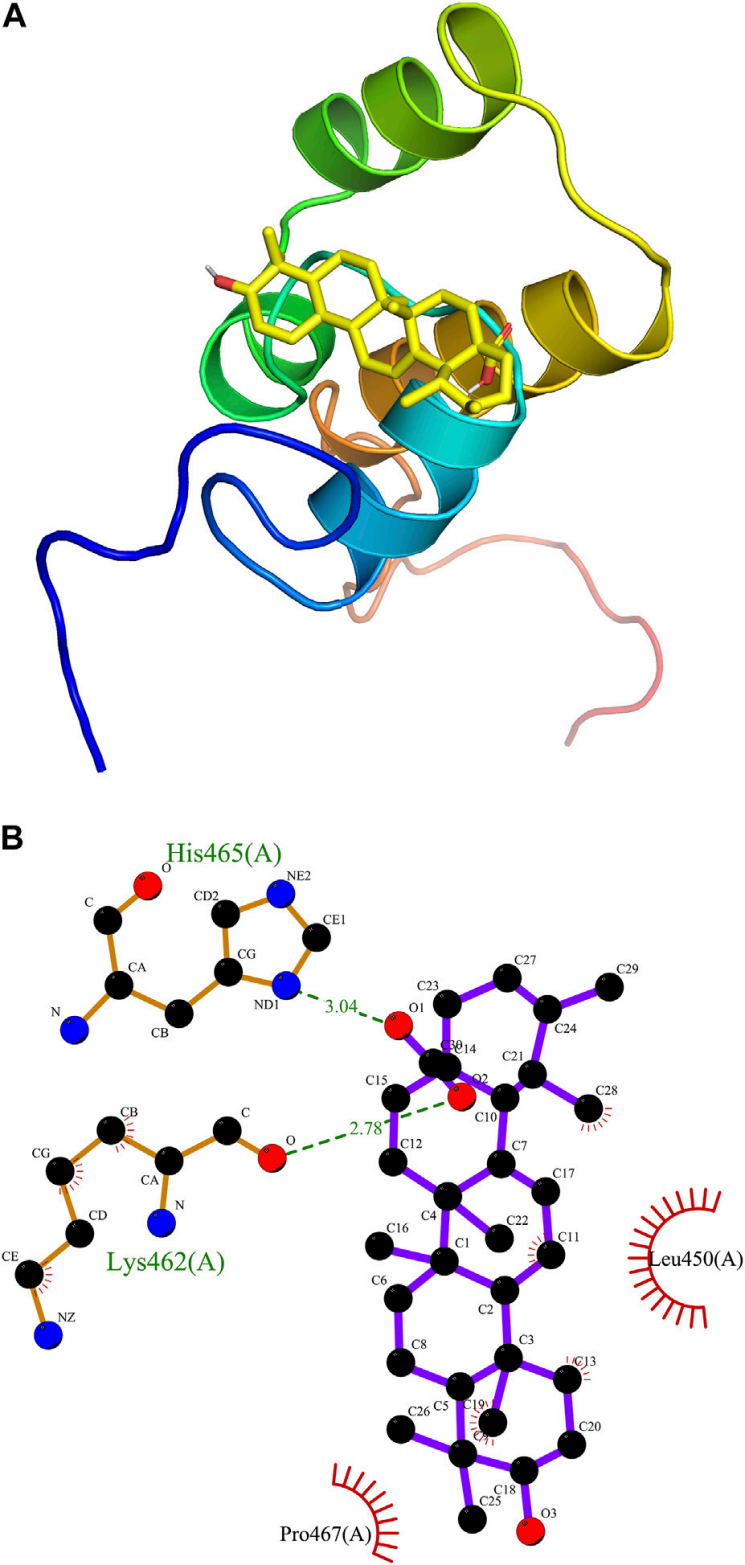
Molecular docking. Predicted docking pose of ursolic acid in complex with Nrf2 (PDB ID: 7O7B). **(A)** Overall structure of Nrf2/Ursolic acid complex. **(B)** LigPlot 2D structure analysis of protein-ligand interaction showing interactions between Nrf2 and ursolic acid. Green dashed lines indicate hydrogen bonds. In addition, ursoilc acid also interacts with Pro467 and Leu 450 via hydrophobic interactions, which are shown as arcs (red).

For a better understanding of the correlation between ursolic acid and Nrf2 domain, AutoDock Vina (Molecular Graphics Lab at the Scripps Research Institute) was employed for the receptor and ligand docking analysis. An interacting complex of Nrf2 and ursolic acid was obtained after docking the molecular proteins with different conformations and poses. The 3D molecular simulation illustrate the binding modes of ursoilc acid with Nrf2 via the hydrogen bond and other interactions (Figure 7A). The binding energy between Nrf2 and ursolic is −6.9 kCal/mol. In Figure 7B, Nrf2 primarily relied on hydrogen bonds to interact with ursolic acid, ursolic acid forms a hydrogen bond (2.78 Å) with Lys462 and a hydrogen bond (3.04 Å). In addition, ursoilc acid also interacts with Pro467 and Leu 450 via hydrophobic interactions, which are shown as arcs (red) in Figure 7B.

## Discussion

In the present study, we found that UA could reduce EAM-induced myocardial injury through the oxidative stress pathway. UA ameliorated EAM-induced myocardial injury by scavenging ROS and activating the Nrf2/HO-1 pathway.

Despite the host-activated immune response, many studies have confirmed the presence of multiple anti-myocardial antibodies in patients with myocarditis and DCM, the most common of which are anti-cardiac myosin antibodies (Caforio et al., 2008). In the present study, the EAM model in mice is induced by immunizing mice with myocardial self-antigens, which simulates the autoimmunological process of acute myocarditis (Pando et al., 2010; Zempo et al., 2015). An autoimmune response is a common pathological process of myocarditis caused by various pathogenic factors. The EAM model has been used in many studies to investigate the pathogenesis of clinical myocarditis and post-myocarditis cardiac dysfunction (Tada and Suzuki, 2016). Therefore, we select the EAM model to further investigate the role and potential mechanisms of UA in protection against myocarditis.

In an abnormal immune response in the myocardium, antigen presence stimulates the differentiation and proliferation of many T cells in the peripheral immune organs, which migrates from the blood circulation to the heart. Lymphocytes infiltrate the myocardium and release inflammatory cytokines such as IL-6, IL-17, and IL-23, causing cardiomyocyte hypertrophy or death, resulting in myocardial hypertrophy or fibrosis and cardiac systolic or diastolic dysfunction (Vdovenko and Eriksson, 2018; Chang et al., 2019). IL-6 plays an important role in the inflammatory storm of myocarditis. Yamashita et al. demonstrated that blocking IL-6 receptors prior to immunization inhibited the development of autoimmune myocarditis in mice. In a study of autoimmune myocarditis in IL-6-deficient mice, it was found that the prevalence and severity of myocarditis were markedly reduced in the absence of IL-6. These may provide evidences for our selection of IL-6 as the primary inflammatory agent. Therefore, in the present study, we provide experimental evidence that IL-6 stimulation of H9c2 cells causes ROS production and cell apoptosis (Figure 6).

In recent years, Nrf2 has been shown to have a protective role in major cardiovascular diseases (CVDs) in murine models. It can improve cardiac function and reduce efferocytosis and the release of pro-inflammatory cytokines, thereby improving the pathological phenotype (Gu et al., 2017; Shen et al., 2019; Chen et al., 2021). Nrf2 is the master regulator of antioxidant responses. The activation of the Nrf2-dependent antioxidant system is important in cellular defense against oxidative stress damage, and the deficiency of the Nrf2 system may pose a significant risk to the cardiovascular system (da Costa et al., 2019). Curcumin, a natural Nrf2 activator, inhibits oxidative stress and inflammation caused by free fatty acid treatment of cardiomyocytes, implying that Nrf2 is closely associated with the risk of obesity-related metabolic cardiovascular disease (Zeng et al., 2015). In experimental diabetic cardiomyopathy, reduced Nrf2 activity was observed, as was antioxidant enzyme activity downstream and increased oxidative stress (Wang et al., 2017). In addition, numerous studies have demonstrated that Nrf2 protects against atherosclerosis by reducing vascular smooth muscle cell (VSMC) migration, proliferation, calcification, and vascular remodeling (Duckers et al., 2001; Ashino et al., 2013; Ashino et al., 2016), although it has not been thoroughly studied that Nrf2-ARE may play a regulatory role in myocarditis (Tada and Suzuki, 2016). Our findings revealed that EAM downregulates the Nrf2 protein expression and reduces the nuclear entry of Nrf2. Moreover, EAM downregulates Nrf2 transcription regulation of phase II antioxidant enzyme HO-1, increases the oxidative stress-induced lipid peroxidation index Malondialdehyde (MDA) and cell apoptosis in heart. Under oxidative conditions, Nrf2 translocates from the cytoplasm to the nucleus, which binds to AREs to regulate downstream genes, such as HO-1, in the present study. These findings are consistent with a previous study that describe the role of Nrf2 upregulation in protecting the EAM-induced cardiomyocyte from oxidant stress damage (Jaén et al., 2020). We determined that UA can reduce damage associated with oxidative stress in the EAM model by activating the Nrf2 pathway and its antioxidant targets. To further validate this view, in the present study, we identified Nrf2 as the primary target of UA through the inhibition of Nrf2 by ML385, which nearly reversed the beneficial effects of UA on apoptosis and oxidative stress damage.

Currently, the prevailing view is that oxidative stress exacerbates the dysfunction and structural remodeling of myocarditis. There are also many studies for the effects of antioxidants. Meanwhile, more and more molecular targets and new drugs have been discovered. However, due to the difficulty of manufacturing the drugs and safety, clinical translation cannot be achieved. In contrast, ursolic acid has following advantages, which make it promising as an antioxidant to assist in EAM treatment: 1) UA is a natural product ingredient that can be extracted from natural plants (e.g., apple peels) in plentiful quantities and at a relatively cheap price; 2) UA probably provides a better safety profile in clinical use, even people with a high risk of myocarditis may benefit from taking UA as a health supplement. Furthermore, the findings of the present study on the regulatory role of UA in EAM broaden our understanding of the multiple functions of UA in the heart. These findings could serve as a model for drug development in autoimmune myocarditis. The results, along with previous studies on the role of UA in myocardial infarction and atherosclerosis, suggest that UA may be useful for various clinical indications in CVDs.

However, there are several limitations to present study. To further validate the role of Nrf2/HO-1 signaling pathway in H9c2, we selected Nrf2 inhibitor, ML385, rather than Nrf2 siRNA, which is relatively less persuasion. Furthermore, the antioxidant mechanisms of ursolic acid are complex and still need further study.

## Conclusion

**FIGURE 8:**
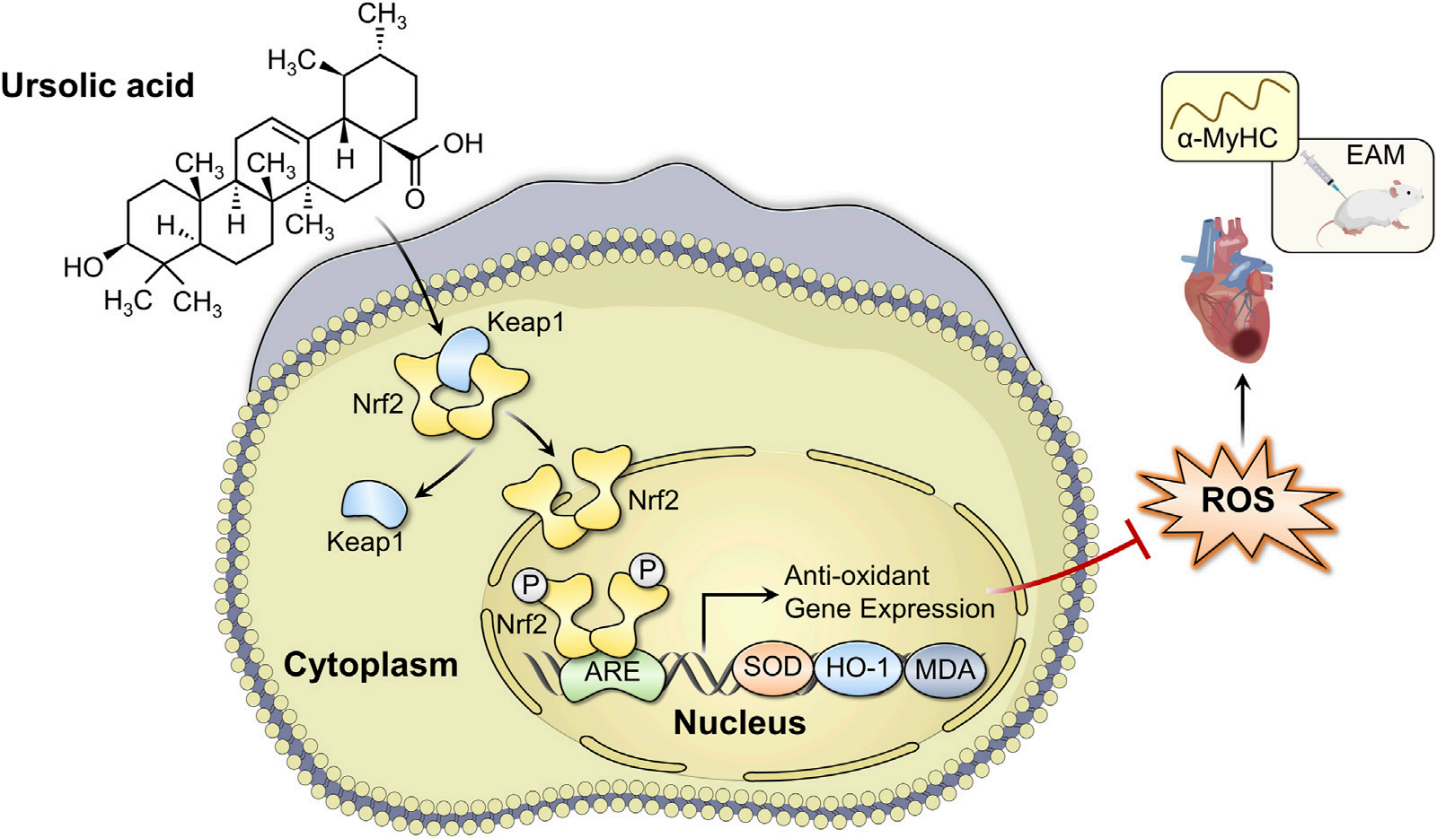
Schematic diagram of the mechanism of ursolic acid in the treatment of experimental autoimmune myocarditis.

In conclusion, we have demonstrated that UA administration could alleviate EAM-induced myocardial injury, as evidenced by improvements in cardiac functions, cardiac inflammation, and structural remodeling, partially by attenuating oxidative stress in a Nrf2/HO-1 pathway-dependent manner (Figure 8). The present study indicates that UA may be a therapeutic candidate drug for the treatment of myocarditis.

## Data Availability

The raw data supporting the conclusion of this article will be made available by the authors, without undue reservation.

## Abbreviations

UA, ursolic acid; EAM, experimental autoimmune myocarditis; Nrf2, Nuclear factor erythroid-related factor 2; HO-1, Heme oxygenase-1; NQO1, NAD(P)H quinone dehydrogenase 1; GSH-Px, Glutathione peroxidase; Elisa, enzyme-linked immunosorbent assay; DHE, Dihydroethidium; MDA, Malondialdehyde; ROS, Reactive oxygen species; AREs, antioxidant response elements; EF, ejection fraction; FS, fractional shortening; LV, left ventricle; cTnI, cardiac troponin I; BNP, brain natriuretic peptide.
