# Supplementary figures and images for: Ursolic acid reduces oxidative stress injury to ameliorate experimental autoimmune myocarditis by activating Nrf2/HO-1 signaling pathway
Source: Front Pharmacol. 2023 Jul 21;14:1189372. doi: 10.3389/fphar.2023.1189372 (PMC10403233; doi:10.3389/fphar.2023.1189372)

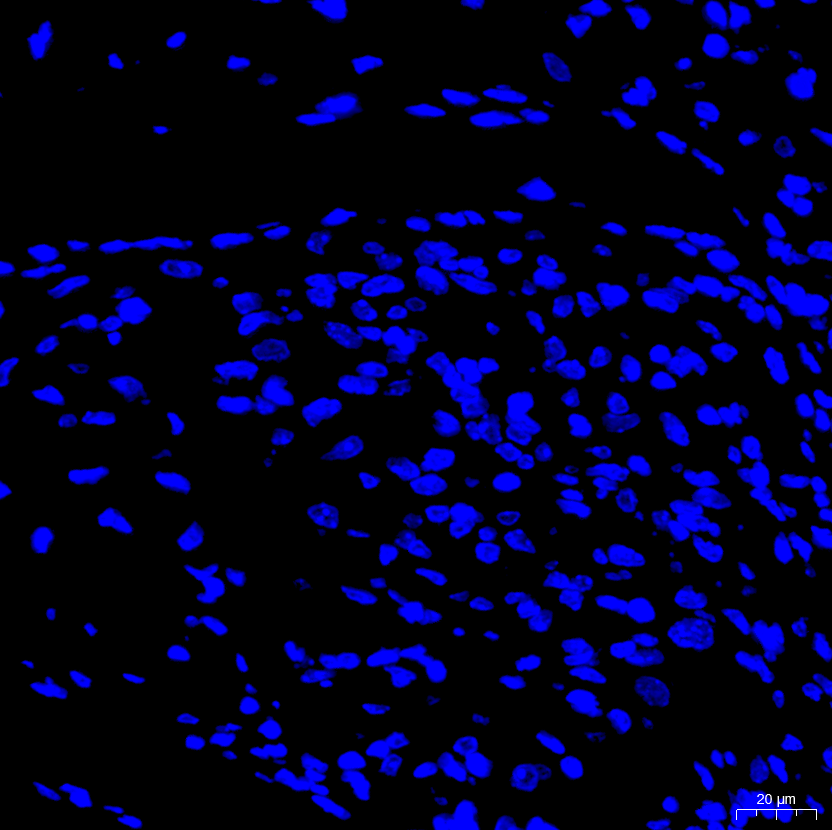

Supplement: Supplementary file 2 [file DataSheet3.ZIP › Raw data-Immunofluorescence,TUNEL,DHE/Figure 1E-IL-6/EAM/DAPI.tif]

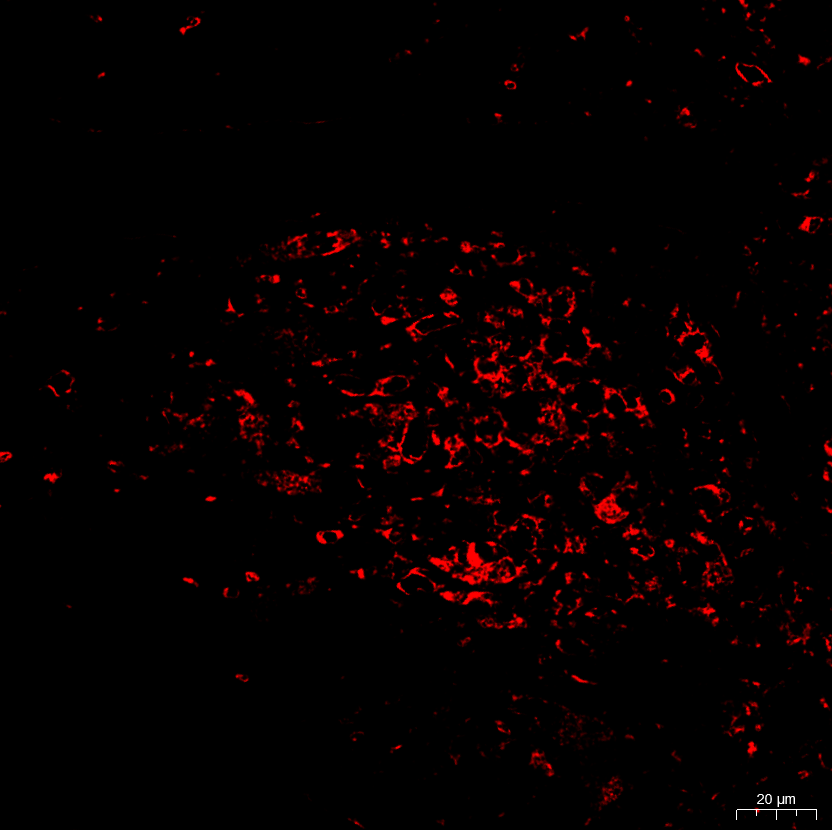

Supplement: Supplementary file 2 [file DataSheet3.ZIP › Raw data-Immunofluorescence,TUNEL,DHE/Figure 1E-IL-6/EAM/IL-6.tif]

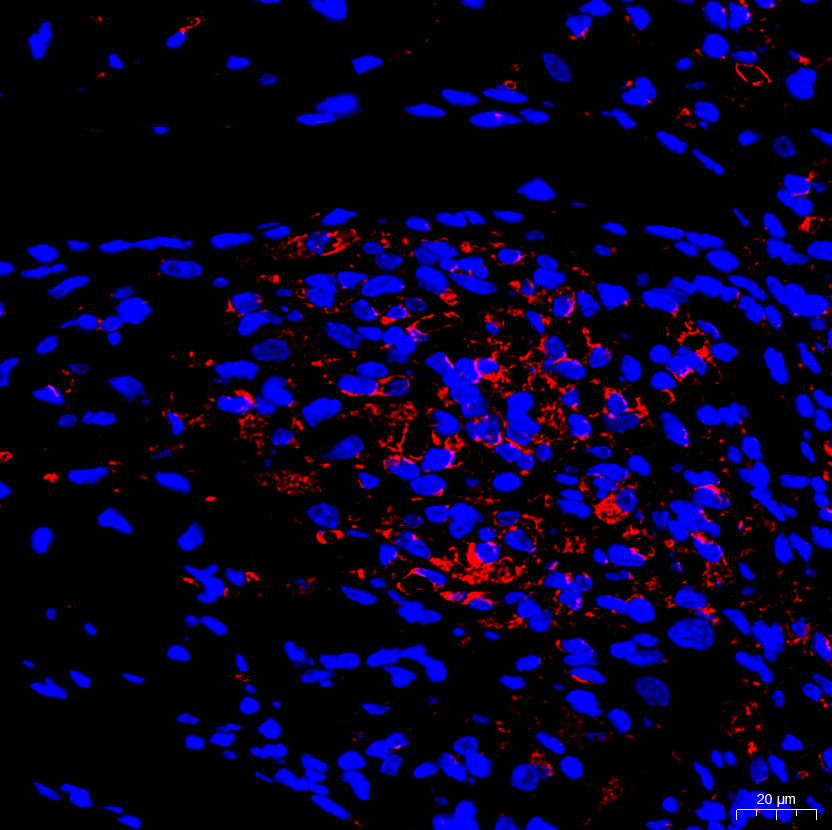

Supplement: Supplementary file 2 [file DataSheet3.ZIP › Raw data-Immunofluorescence,TUNEL,DHE/Figure 1E-IL-6/EAM/Merge.tif]

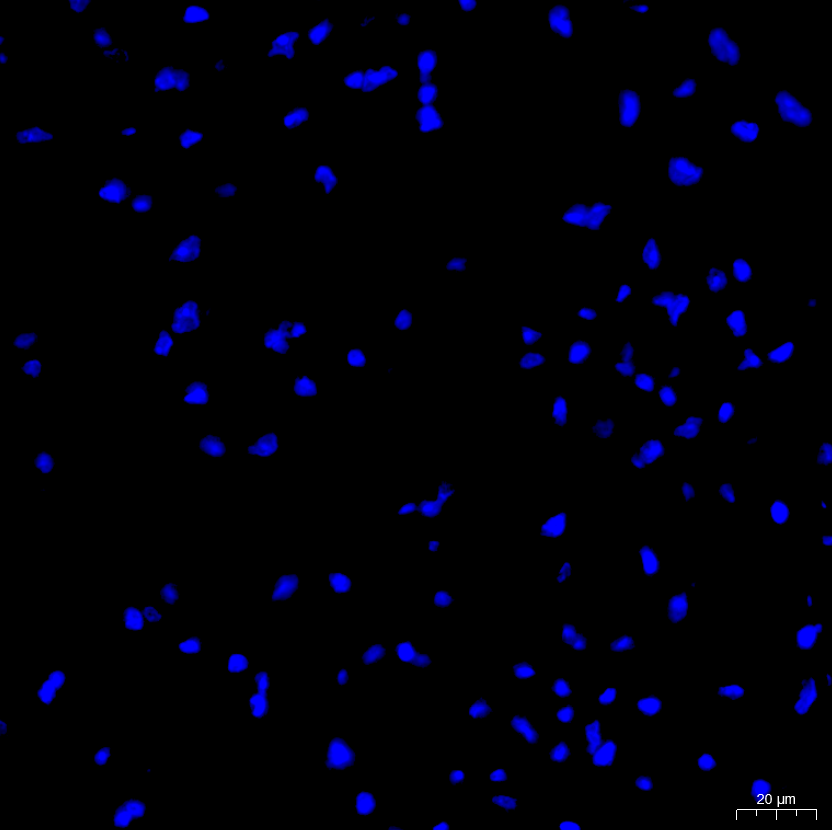

Supplement: Supplementary file 2 [file DataSheet3.ZIP › Raw data-Immunofluorescence,TUNEL,DHE/Figure 1E-IL-6/Sham/DAPI.tif]

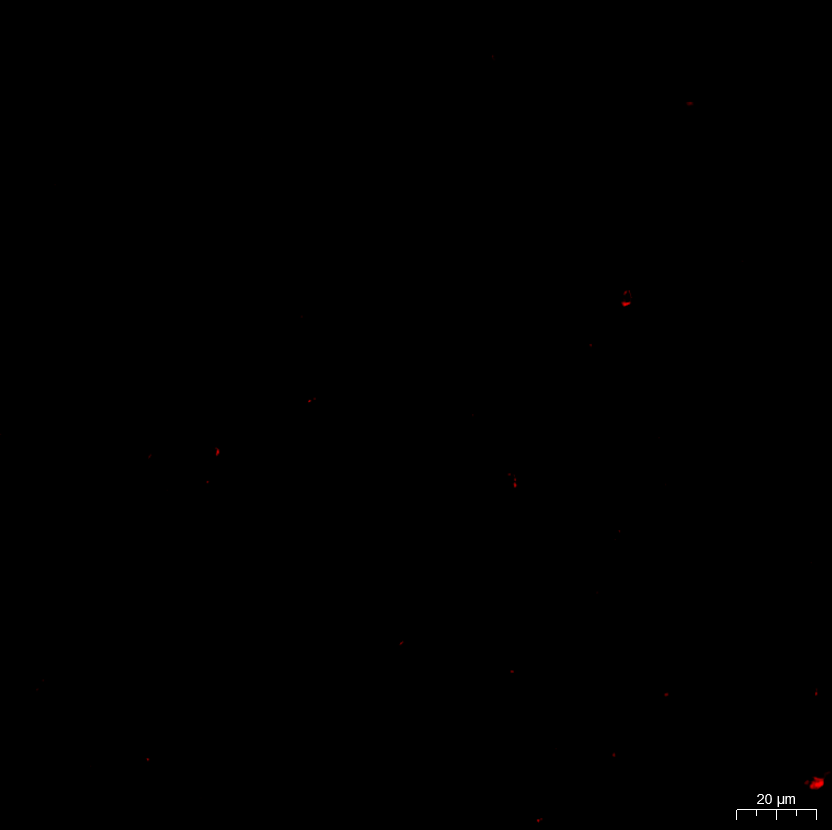

Supplement: Supplementary file 2 [file DataSheet3.ZIP › Raw data-Immunofluorescence,TUNEL,DHE/Figure 1E-IL-6/Sham/IL-6.tif]

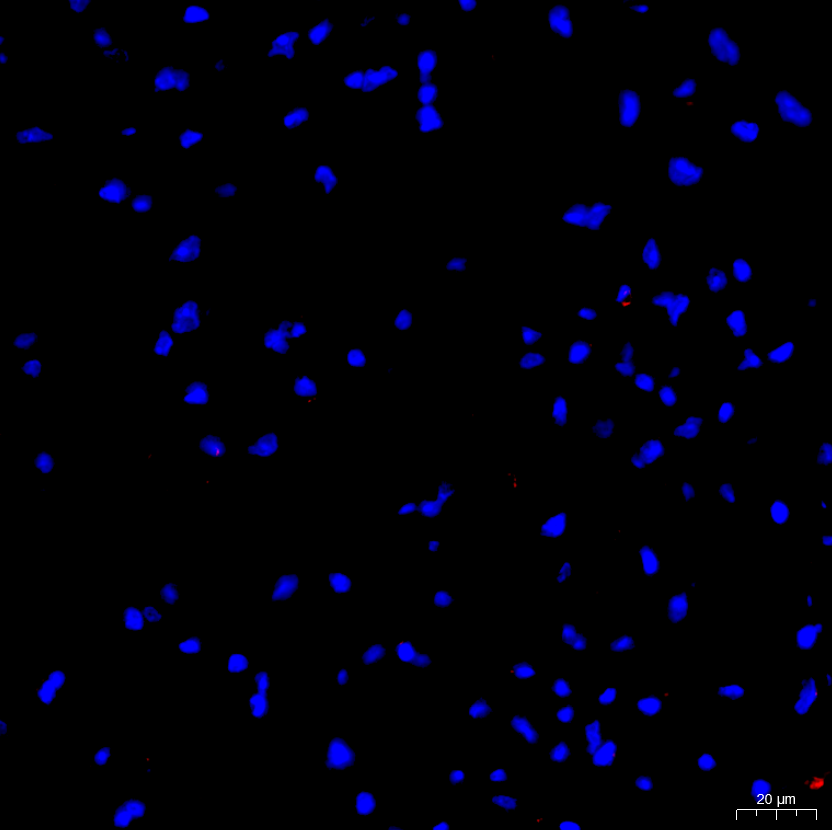

Supplement: Supplementary file 2 [file DataSheet3.ZIP › Raw data-Immunofluorescence,TUNEL,DHE/Figure 1E-IL-6/Sham/Merge.tif]

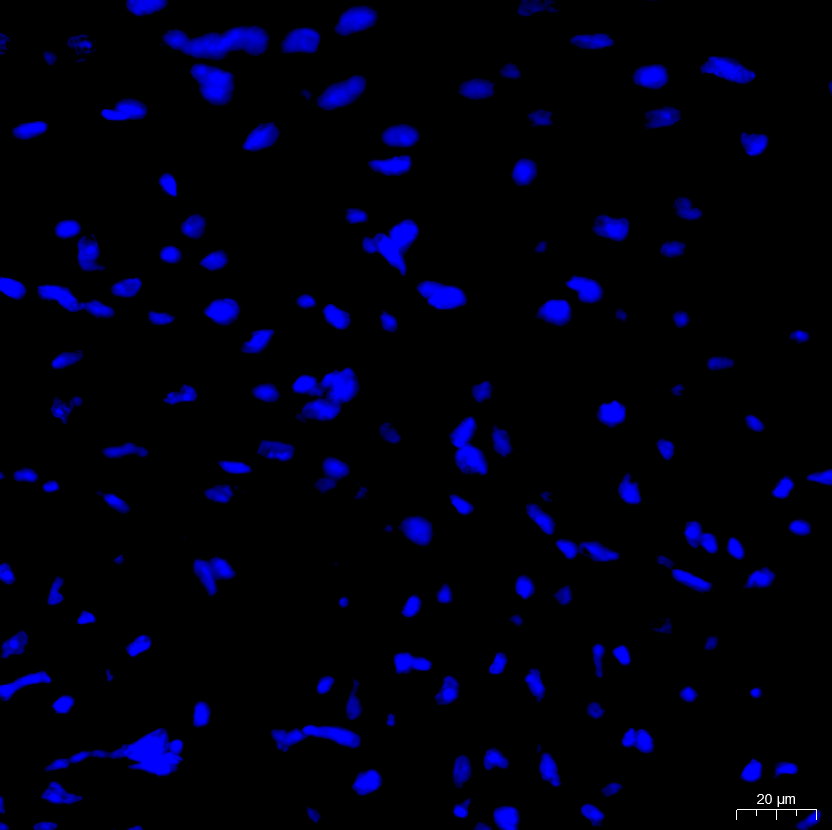

Supplement: Supplementary file 2 [file DataSheet3.ZIP › Raw data-Immunofluorescence,TUNEL,DHE/Figure 1E-IL-6/UA/DAPI.tif]

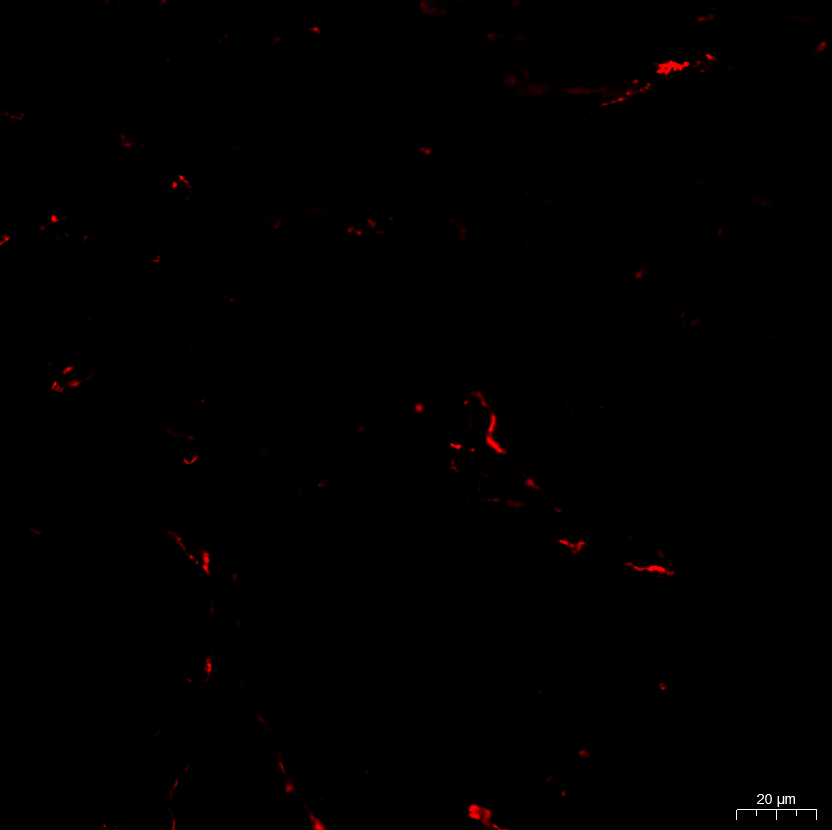

Supplement: Supplementary file 2 [file DataSheet3.ZIP › Raw data-Immunofluorescence,TUNEL,DHE/Figure 1E-IL-6/UA/IL-6.tif]

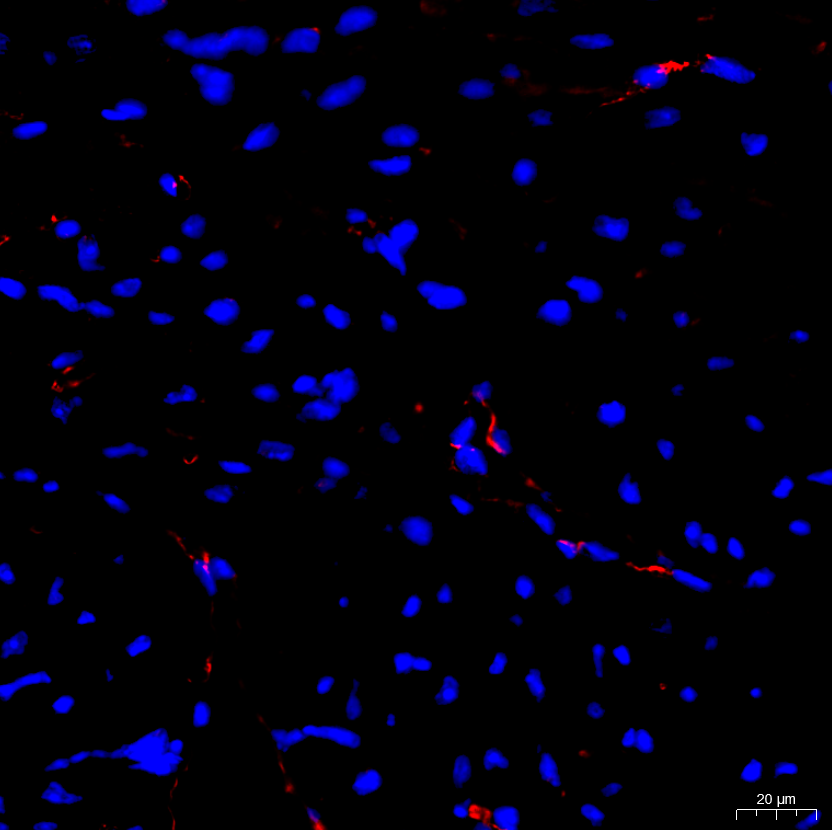

Supplement: Supplementary file 2 [file DataSheet3.ZIP › Raw data-Immunofluorescence,TUNEL,DHE/Figure 1E-IL-6/UA/Merge.tif]

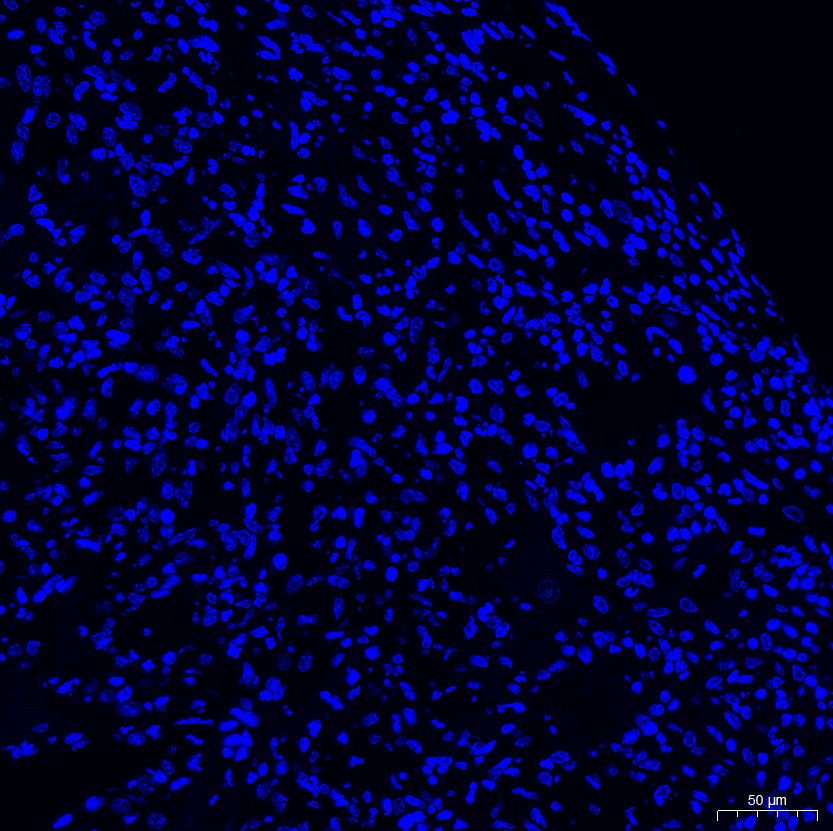

Supplement: Supplementary file 2 [file DataSheet3.ZIP › Raw data-Immunofluorescence,TUNEL,DHE/Figure 3A and 3B-TUNEL/Figure 3A/EAM/DAPI.tif]

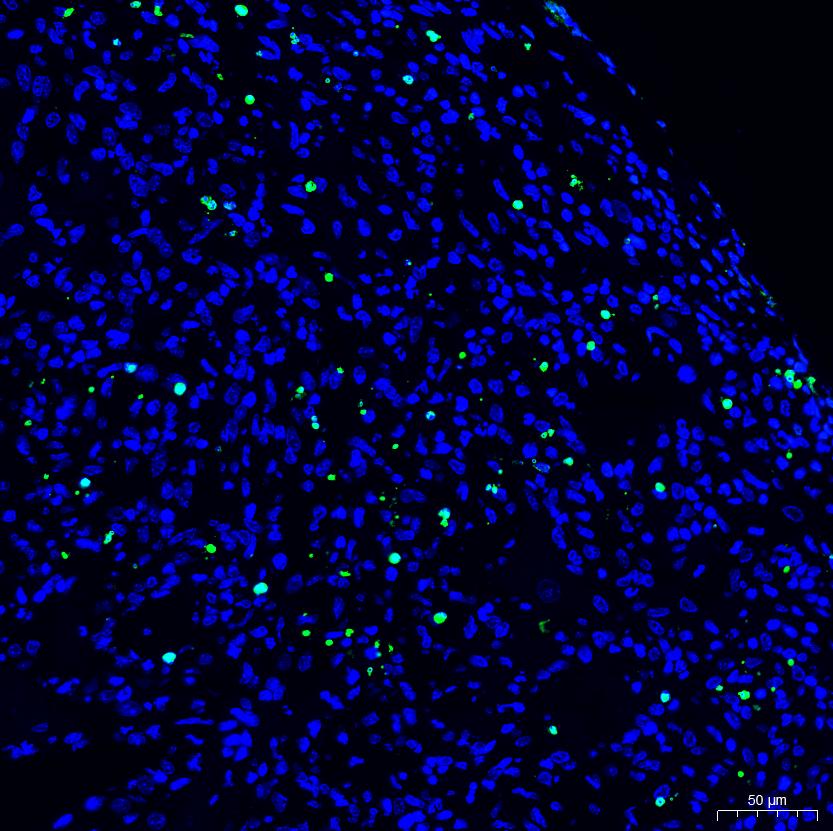

Supplement: Supplementary file 2 [file DataSheet3.ZIP › Raw data-Immunofluorescence,TUNEL,DHE/Figure 3A and 3B-TUNEL/Figure 3A/EAM/Merge.tif]

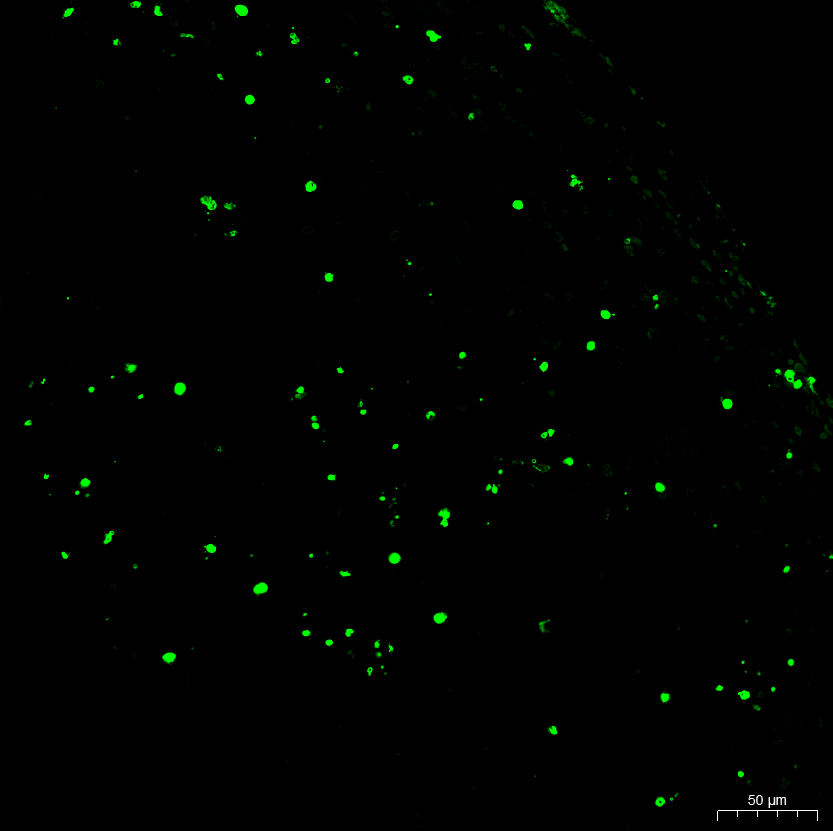

Supplement: Supplementary file 2 [file DataSheet3.ZIP › Raw data-Immunofluorescence,TUNEL,DHE/Figure 3A and 3B-TUNEL/Figure 3A/EAM/TUNEL.tif]

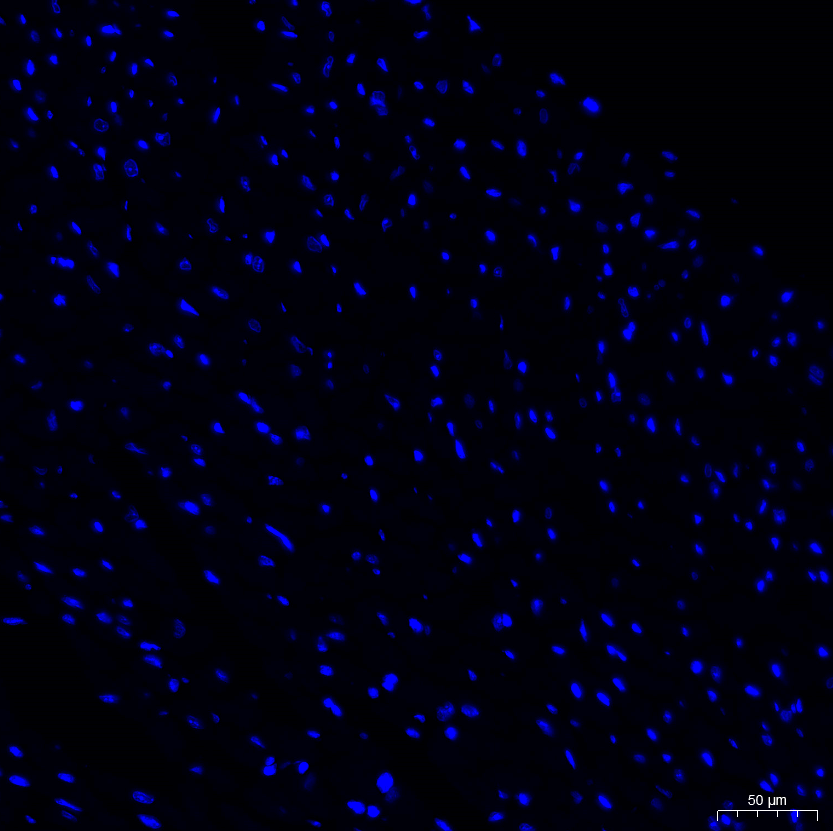

Supplement: Supplementary file 2 [file DataSheet3.ZIP › Raw data-Immunofluorescence,TUNEL,DHE/Figure 3A and 3B-TUNEL/Figure 3A/Sham/DAPI.tif]

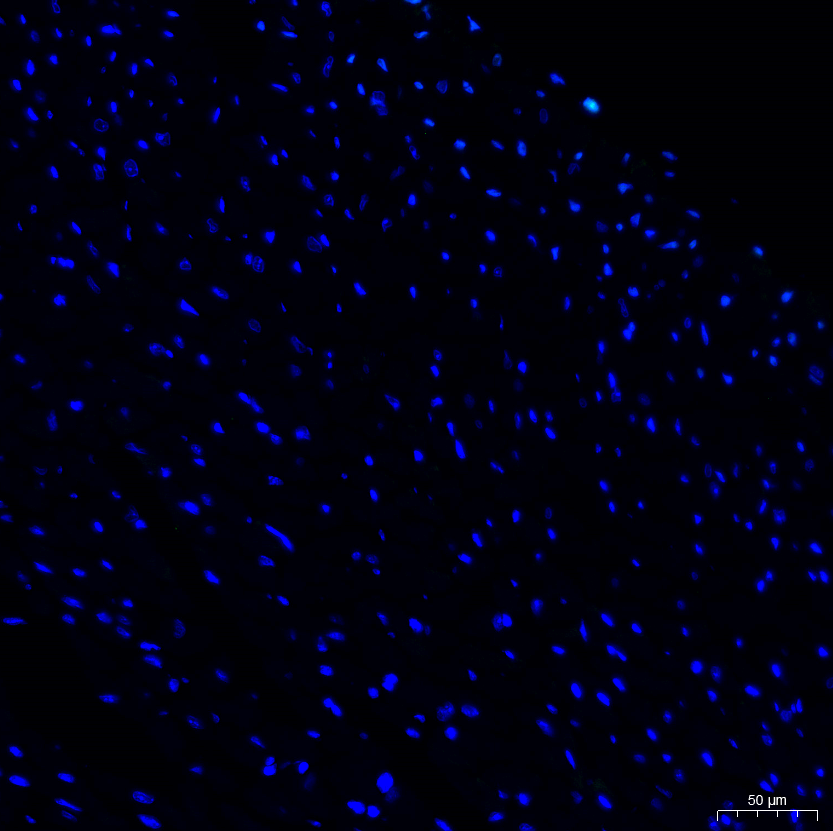

Supplement: Supplementary file 2 [file DataSheet3.ZIP › Raw data-Immunofluorescence,TUNEL,DHE/Figure 3A and 3B-TUNEL/Figure 3A/Sham/Merge.tif]

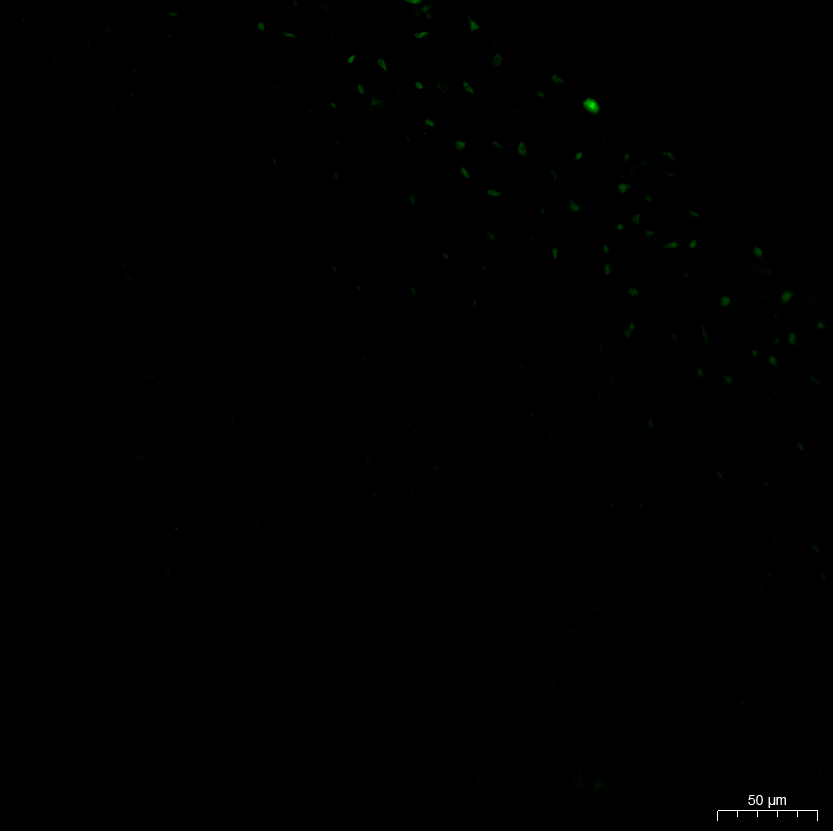

Supplement: Supplementary file 2 [file DataSheet3.ZIP › Raw data-Immunofluorescence,TUNEL,DHE/Figure 3A and 3B-TUNEL/Figure 3A/Sham/TUNEL.tif]

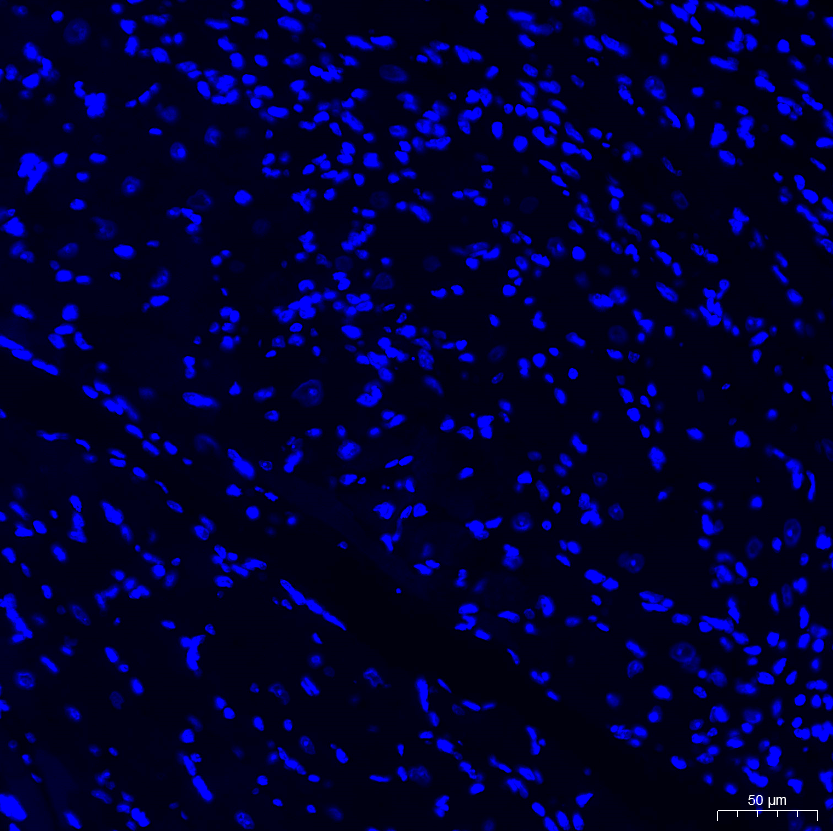

Supplement: Supplementary file 2 [file DataSheet3.ZIP › Raw data-Immunofluorescence,TUNEL,DHE/Figure 3A and 3B-TUNEL/Figure 3A/UA/DAPI.tif]

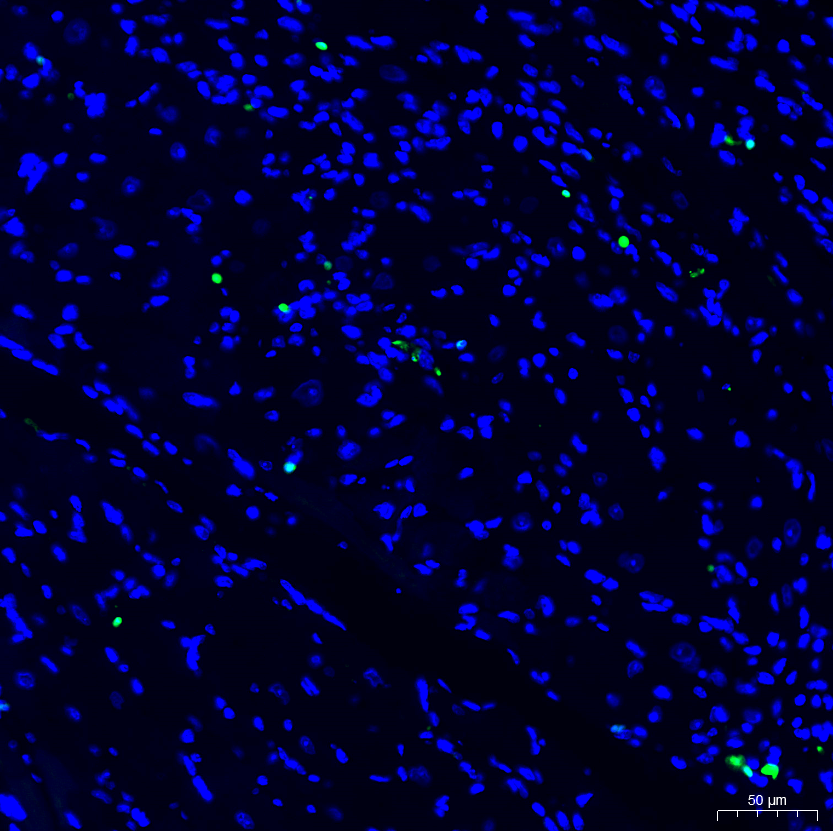

Supplement: Supplementary file 2 [file DataSheet3.ZIP › Raw data-Immunofluorescence,TUNEL,DHE/Figure 3A and 3B-TUNEL/Figure 3A/UA/Merge.tif]

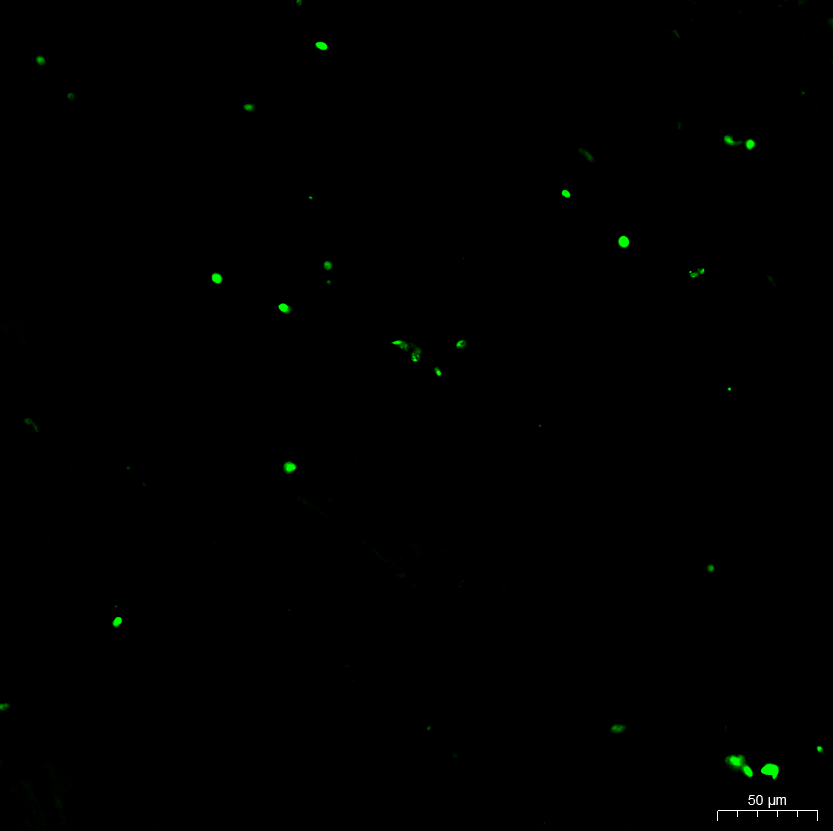

Supplement: Supplementary file 2 [file DataSheet3.ZIP › Raw data-Immunofluorescence,TUNEL,DHE/Figure 3A and 3B-TUNEL/Figure 3A/UA/TUNEL.tif]

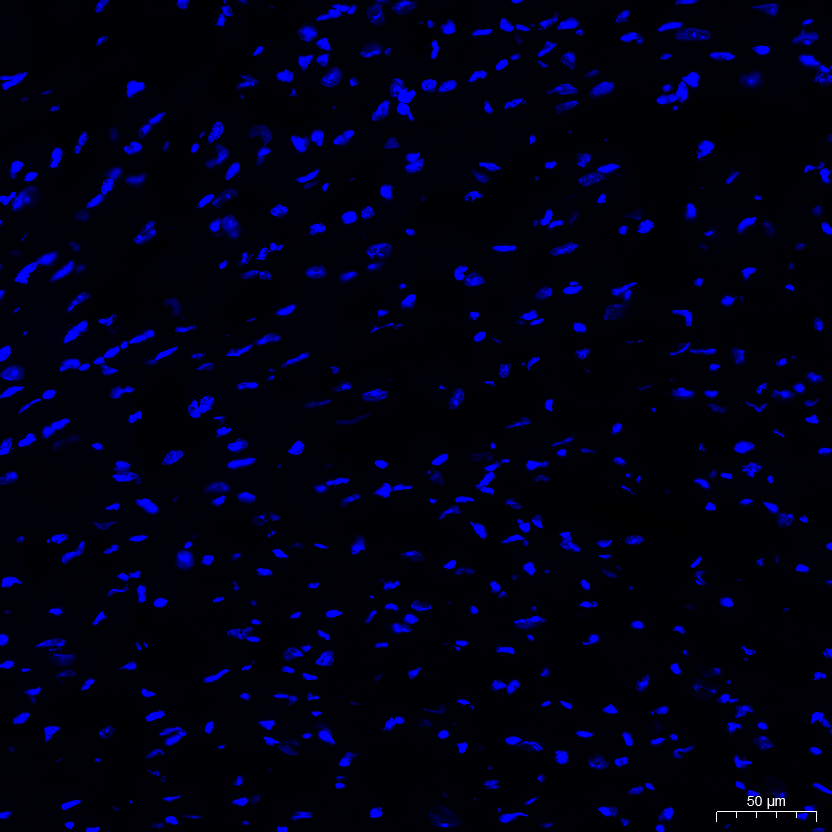

Supplement: Supplementary file 2 [file DataSheet3.ZIP › Raw data-Immunofluorescence,TUNEL,DHE/Figure 4A and 4B-DHE/Figure 4A/EAM/DAPI.tif]

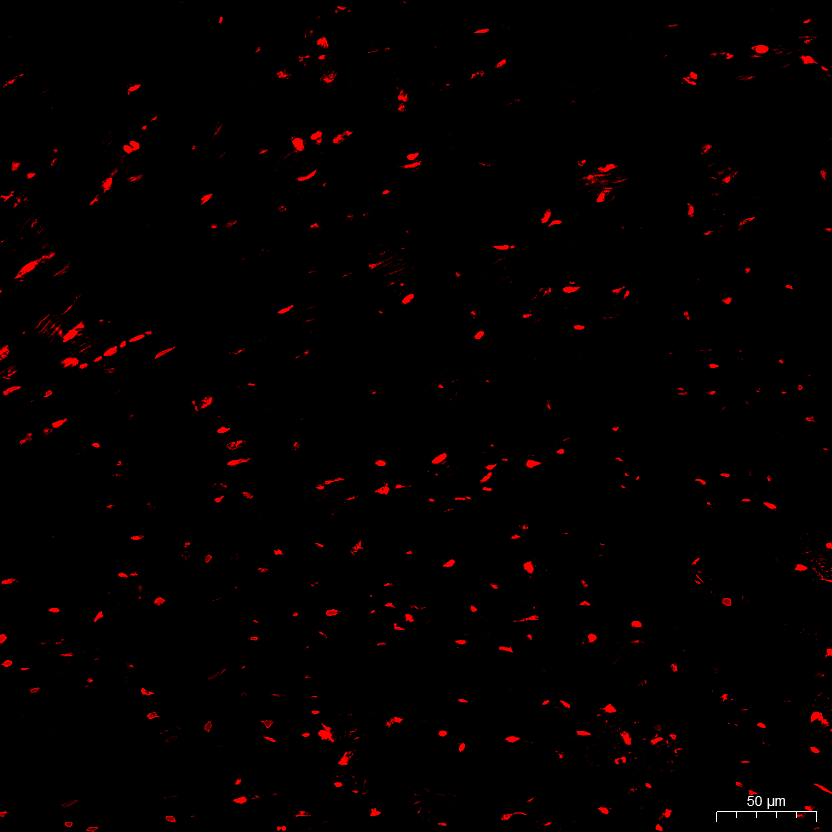

Supplement: Supplementary file 2 [file DataSheet3.ZIP › Raw data-Immunofluorescence,TUNEL,DHE/Figure 4A and 4B-DHE/Figure 4A/EAM/DHE staining.tif]

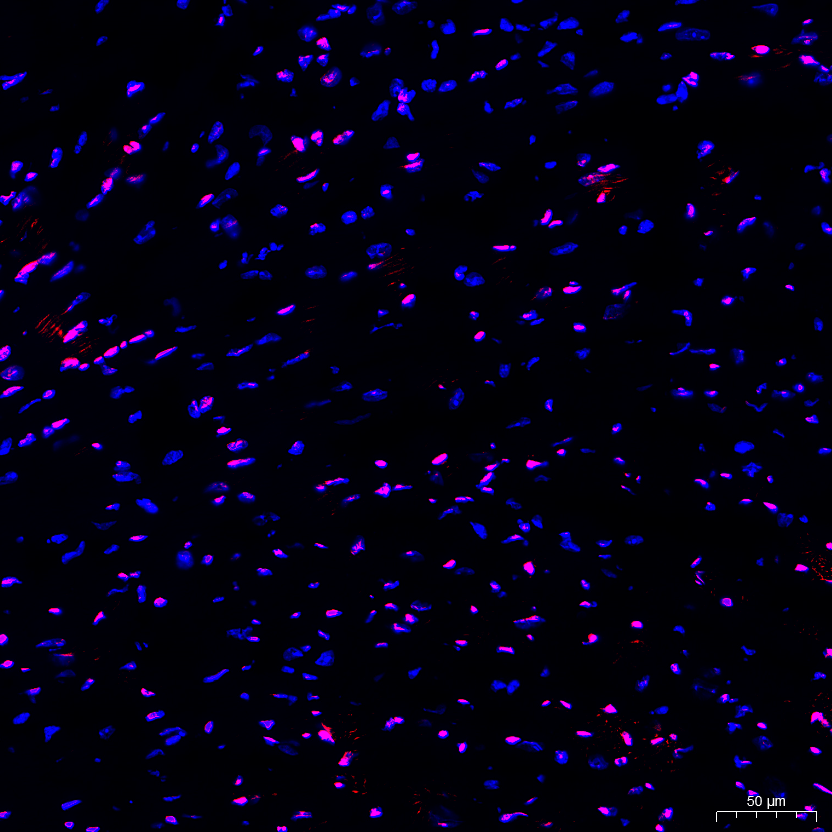

Supplement: Supplementary file 2 [file DataSheet3.ZIP › Raw data-Immunofluorescence,TUNEL,DHE/Figure 4A and 4B-DHE/Figure 4A/EAM/Merge.tif]

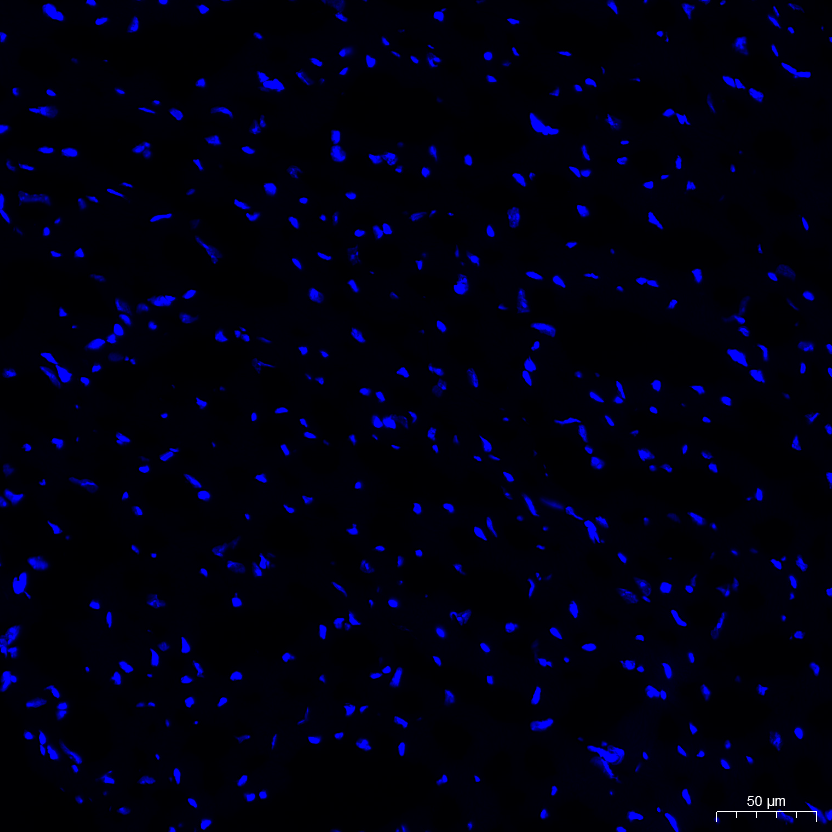

Supplement: Supplementary file 2 [file DataSheet3.ZIP › Raw data-Immunofluorescence,TUNEL,DHE/Figure 4A and 4B-DHE/Figure 4A/Sham/DAPI.tif]

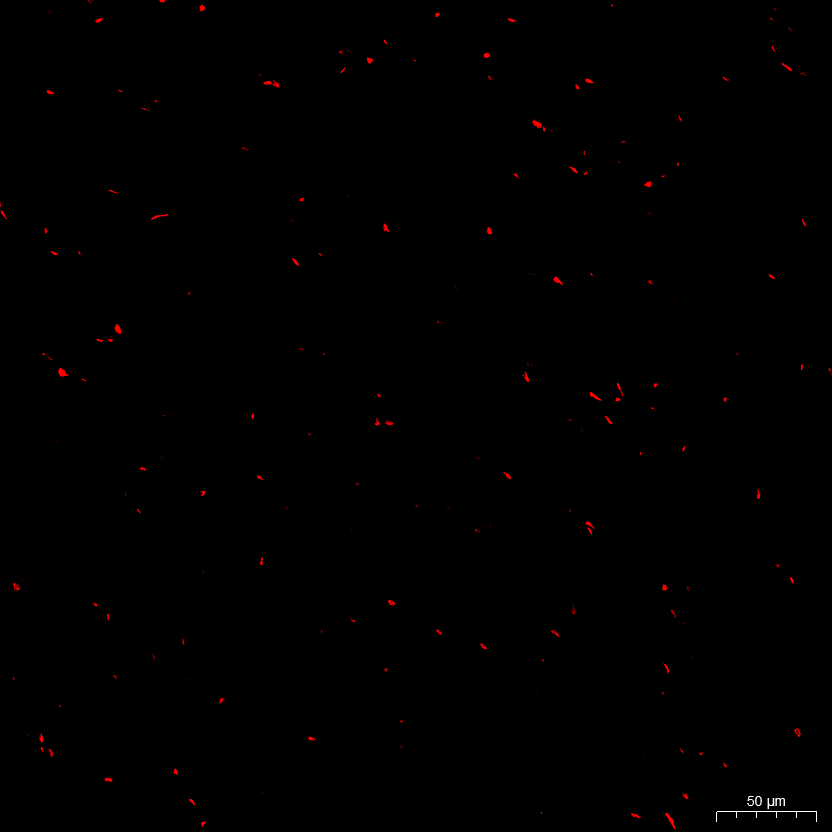

Supplement: Supplementary file 2 [file DataSheet3.ZIP › Raw data-Immunofluorescence,TUNEL,DHE/Figure 4A and 4B-DHE/Figure 4A/Sham/DHE staining.tif]

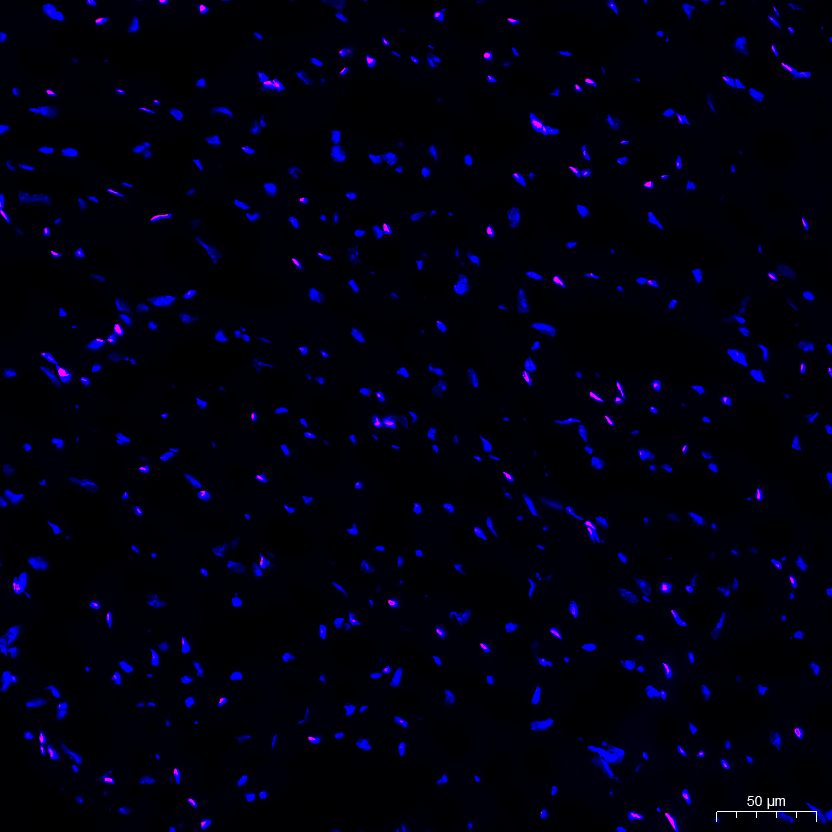

Supplement: Supplementary file 2 [file DataSheet3.ZIP › Raw data-Immunofluorescence,TUNEL,DHE/Figure 4A and 4B-DHE/Figure 4A/Sham/Merge.tif]

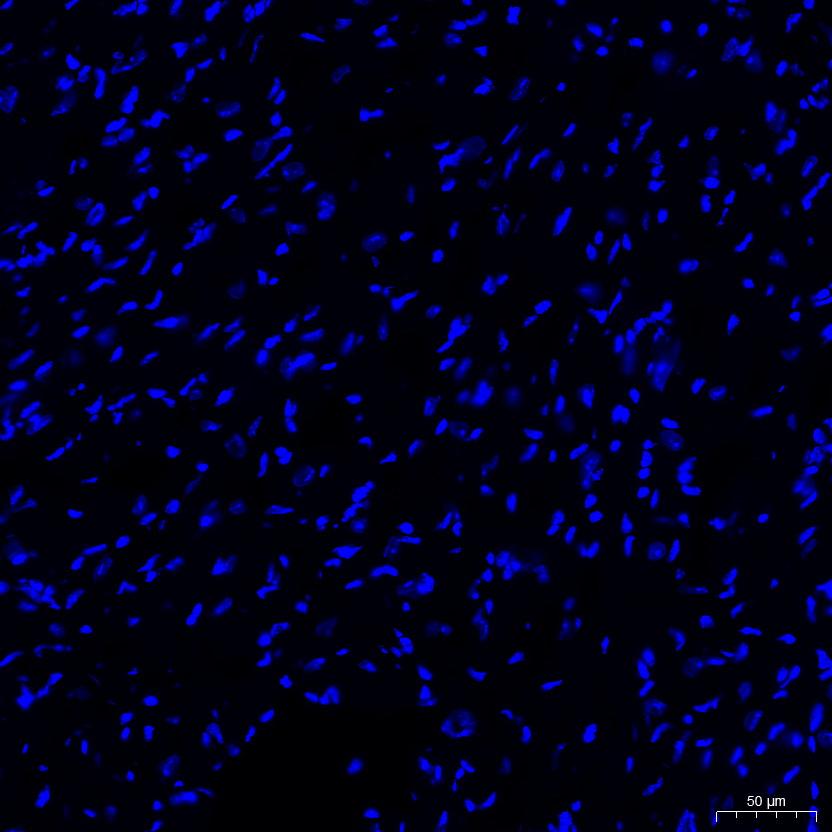

Supplement: Supplementary file 2 [file DataSheet3.ZIP › Raw data-Immunofluorescence,TUNEL,DHE/Figure 4A and 4B-DHE/Figure 4A/UA/DAPI.tif]

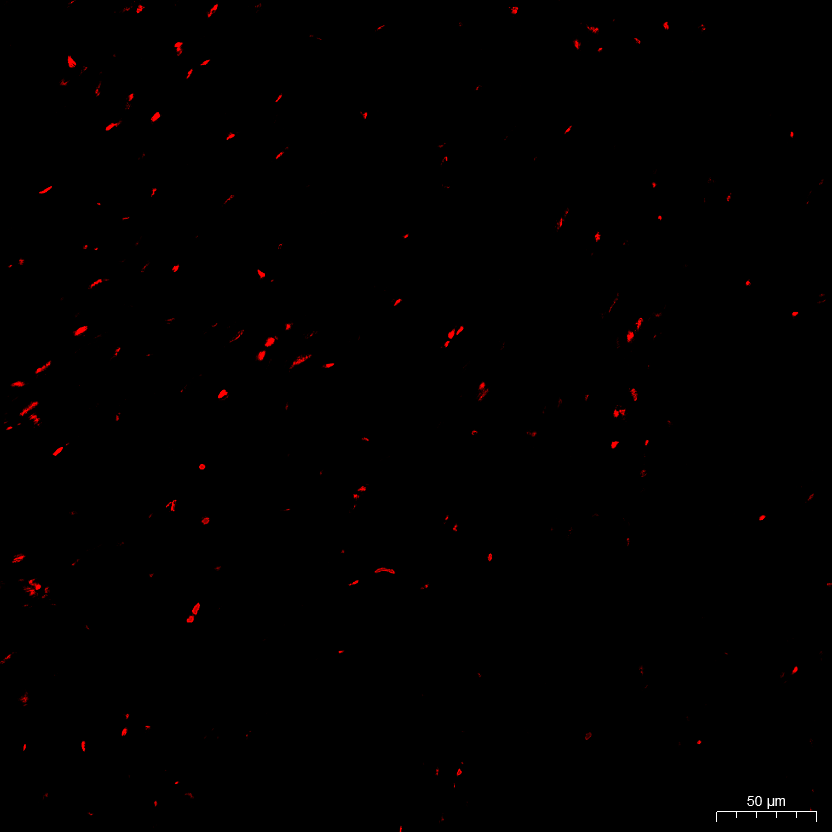

Supplement: Supplementary file 2 [file DataSheet3.ZIP › Raw data-Immunofluorescence,TUNEL,DHE/Figure 4A and 4B-DHE/Figure 4A/UA/DHE staining.tif]

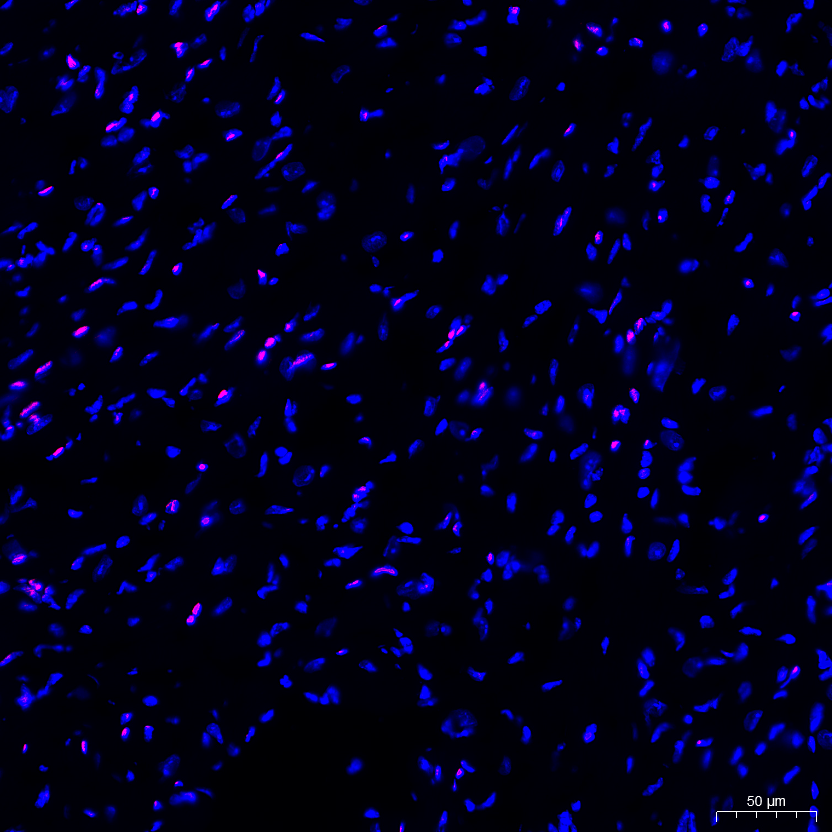

Supplement: Supplementary file 2 [file DataSheet3.ZIP › Raw data-Immunofluorescence,TUNEL,DHE/Figure 4A and 4B-DHE/Figure 4A/UA/Merge.tif]

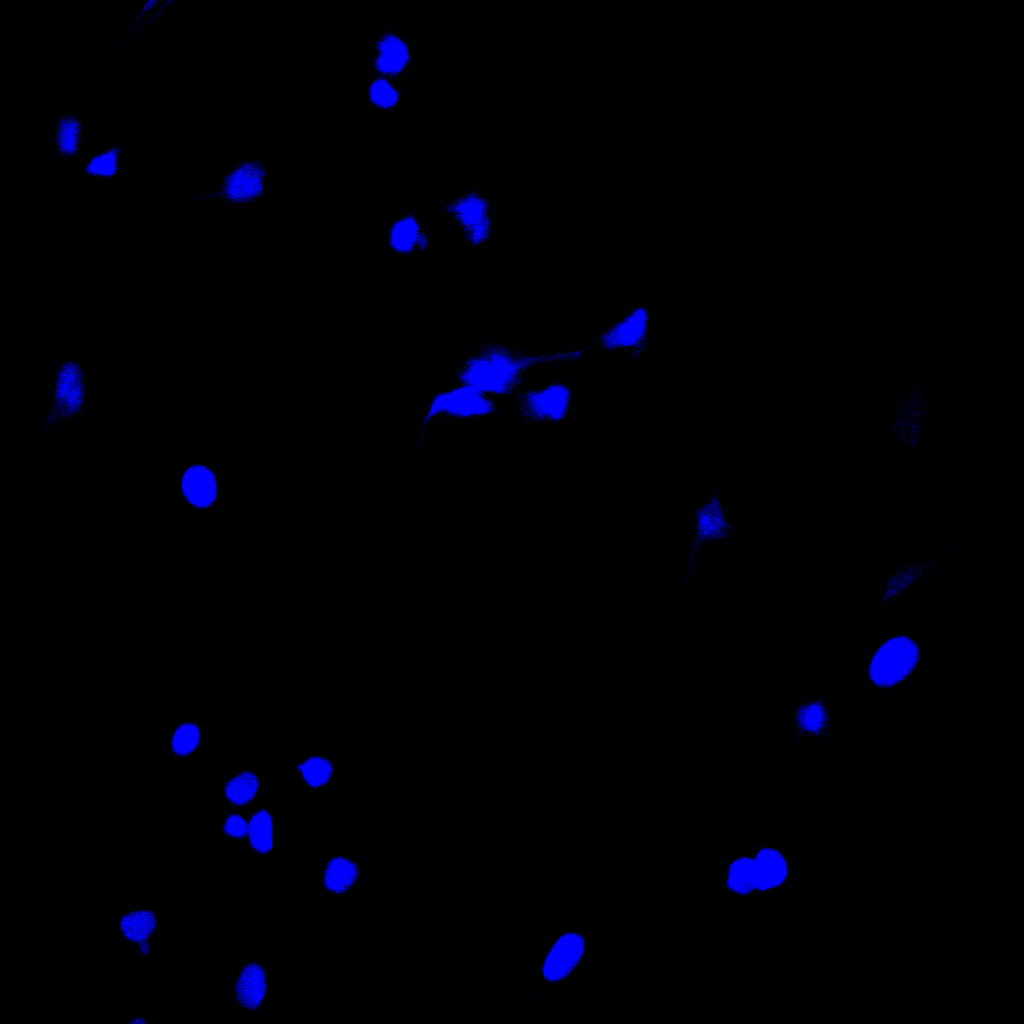

Supplement: Supplementary file 2 [file DataSheet3.ZIP › Raw data-Immunofluorescence,TUNEL,DHE/Figure 6A and 6B-TUNEL/Figure 6A/IL-6/DAPI.tif]

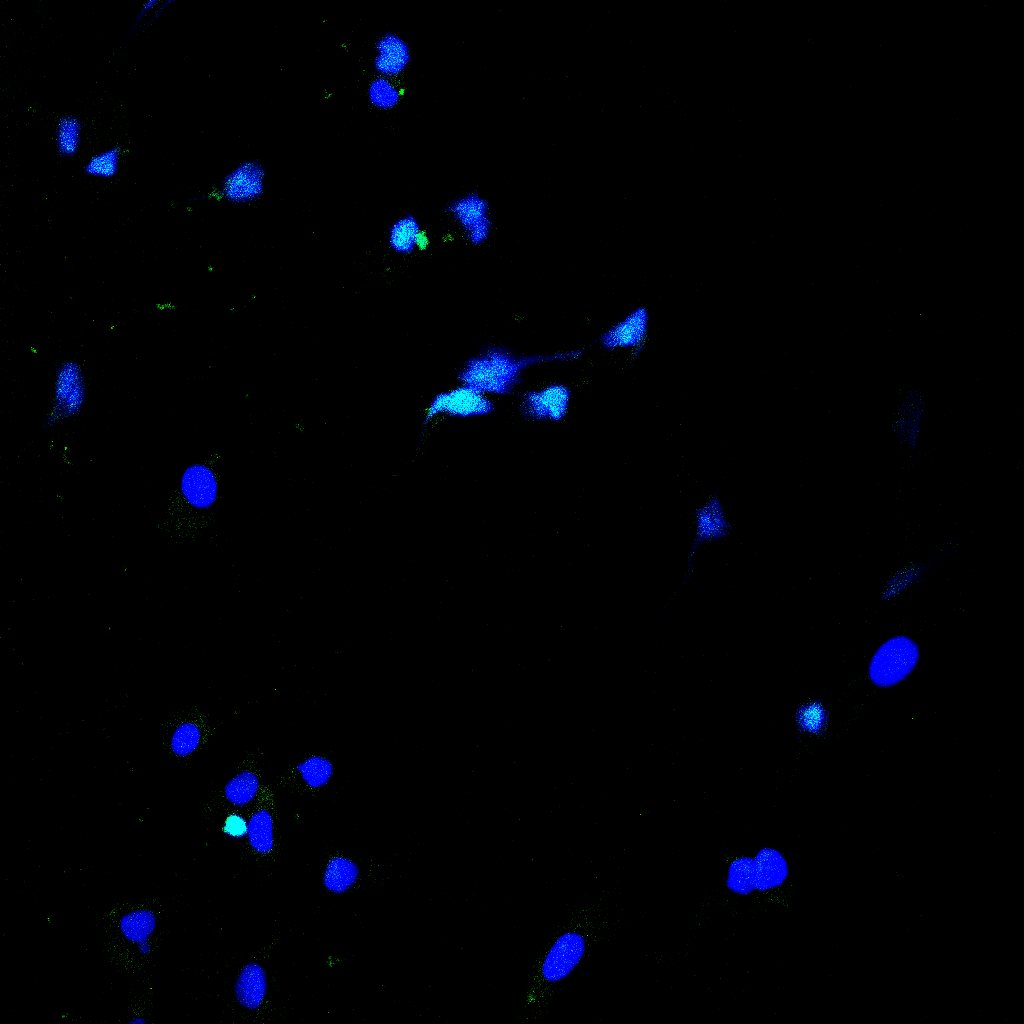

Supplement: Supplementary file 2 [file DataSheet3.ZIP › Raw data-Immunofluorescence,TUNEL,DHE/Figure 6A and 6B-TUNEL/Figure 6A/IL-6/Merge.tif]

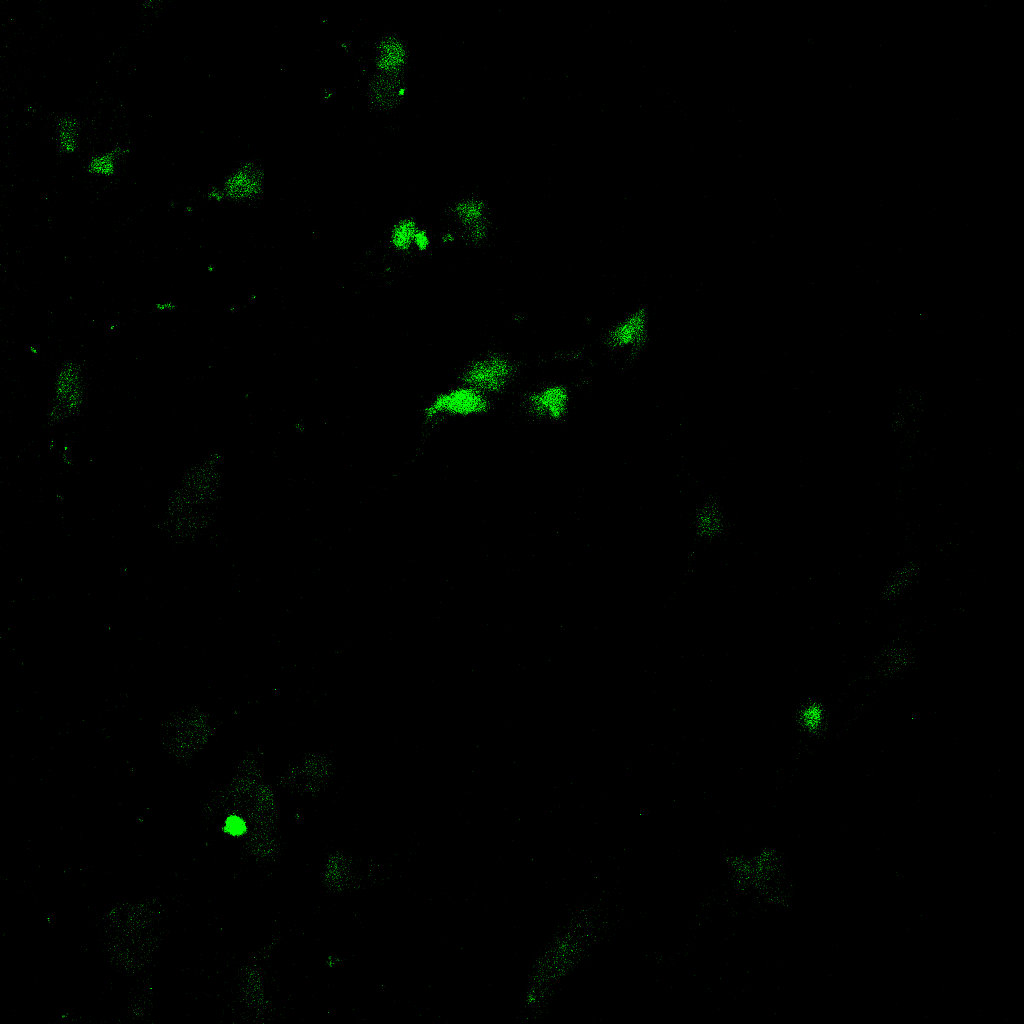

Supplement: Supplementary file 2 [file DataSheet3.ZIP › Raw data-Immunofluorescence,TUNEL,DHE/Figure 6A and 6B-TUNEL/Figure 6A/IL-6/TUNEL.tif]

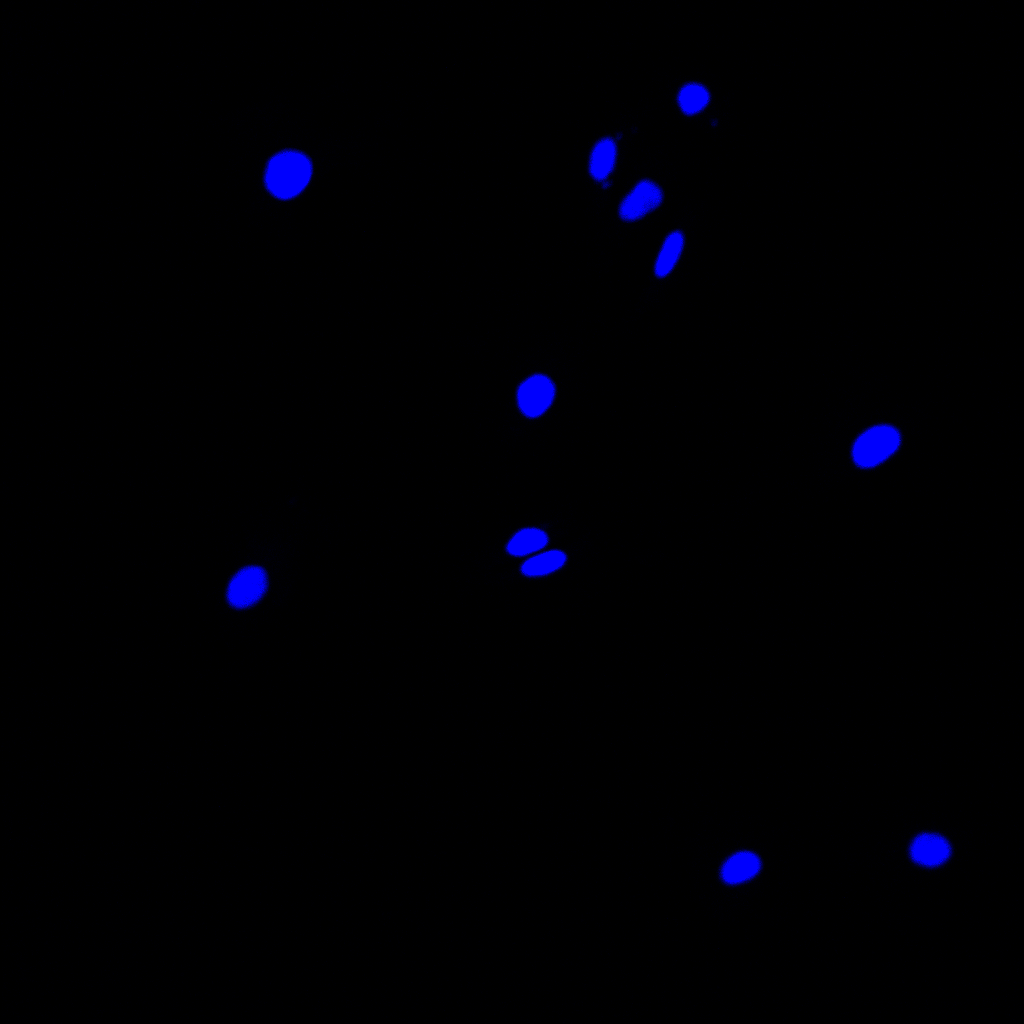

Supplement: Supplementary file 2 [file DataSheet3.ZIP › Raw data-Immunofluorescence,TUNEL,DHE/Figure 6A and 6B-TUNEL/Figure 6A/ML385/DAPI.tif]

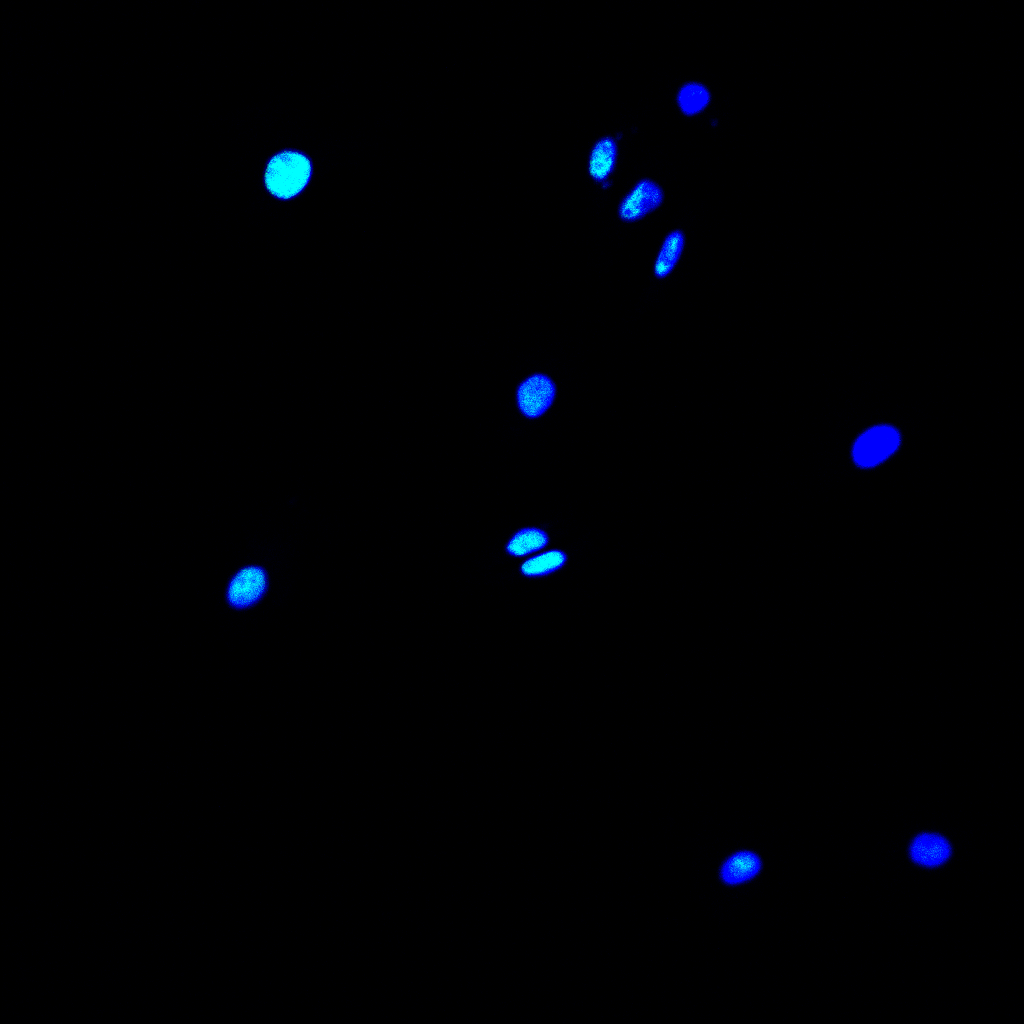

Supplement: Supplementary file 2 [file DataSheet3.ZIP › Raw data-Immunofluorescence,TUNEL,DHE/Figure 6A and 6B-TUNEL/Figure 6A/ML385/Merge.tif]

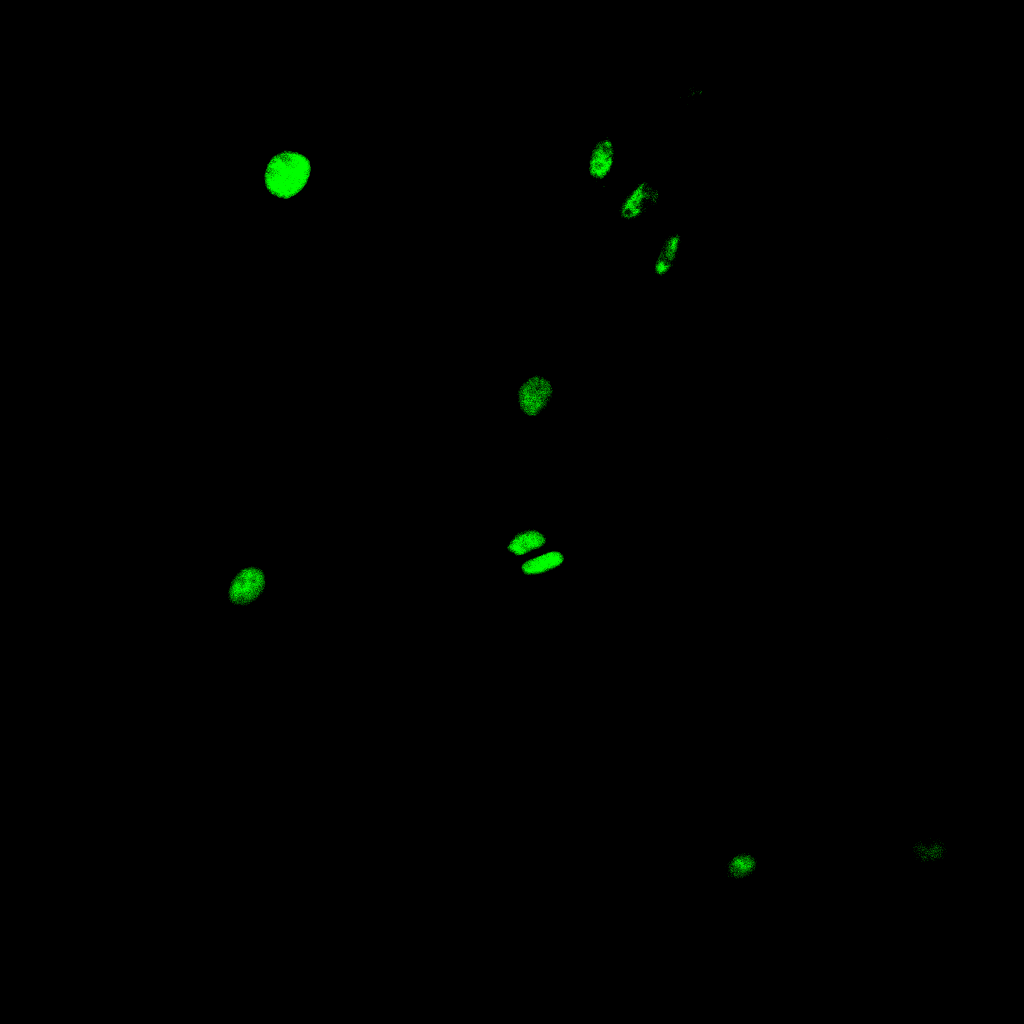

Supplement: Supplementary file 2 [file DataSheet3.ZIP › Raw data-Immunofluorescence,TUNEL,DHE/Figure 6A and 6B-TUNEL/Figure 6A/ML385/TUNEL.tif]

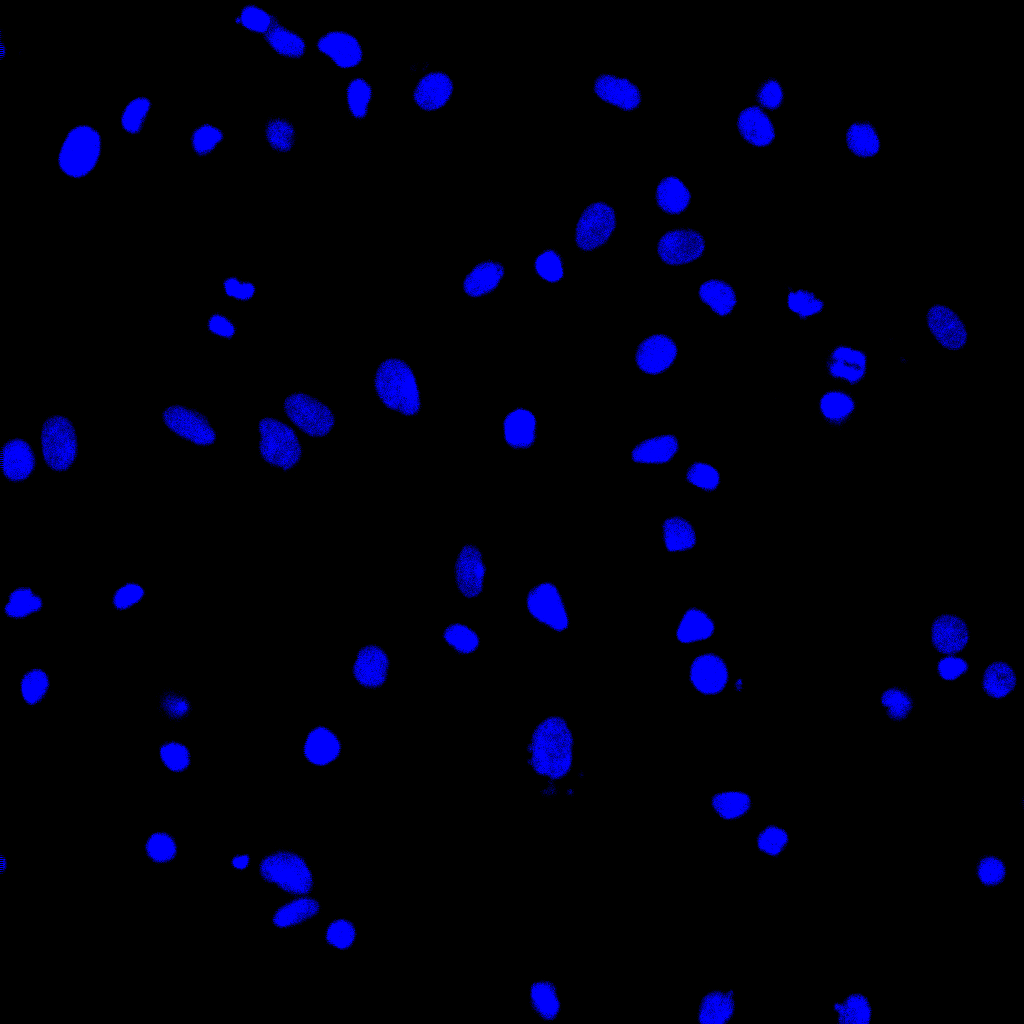

Supplement: Supplementary file 2 [file DataSheet3.ZIP › Raw data-Immunofluorescence,TUNEL,DHE/Figure 6A and 6B-TUNEL/Figure 6A/Sham/DAPI.tif]

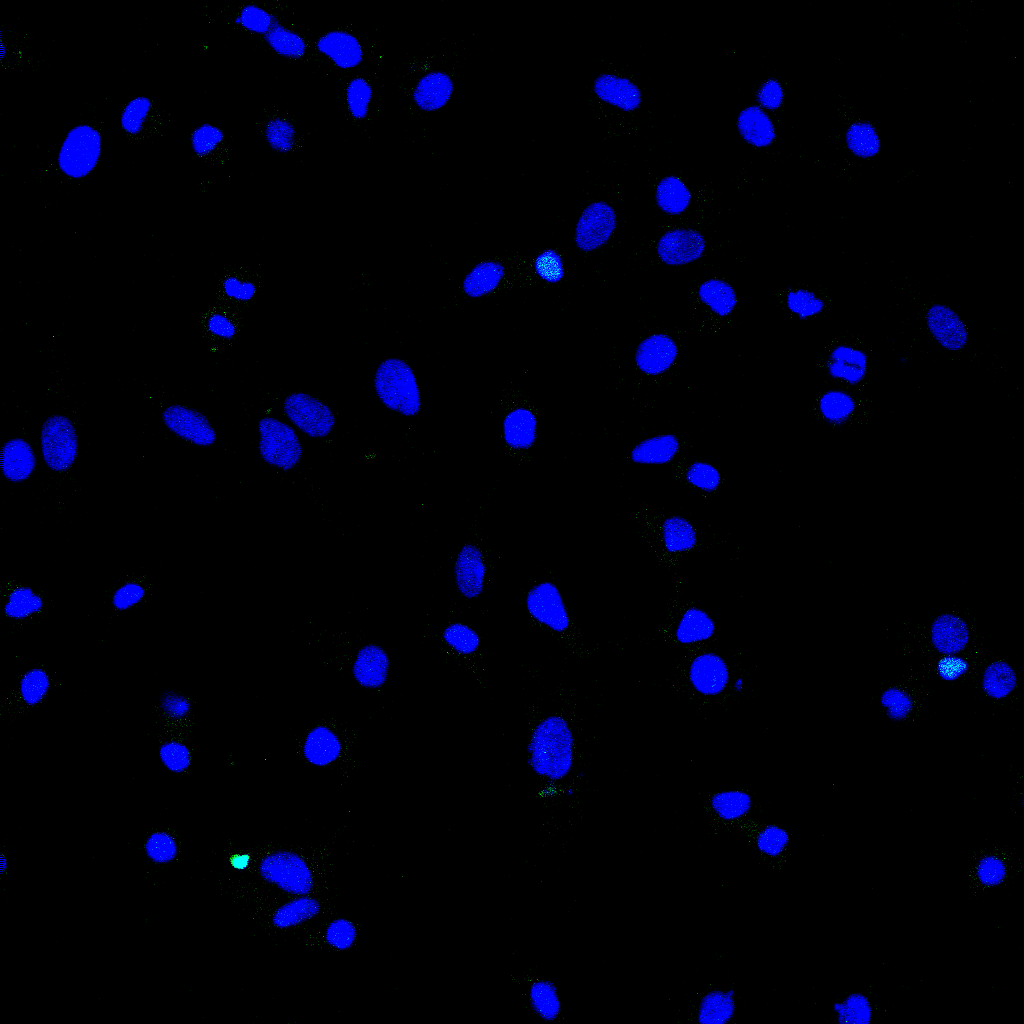

Supplement: Supplementary file 2 [file DataSheet3.ZIP › Raw data-Immunofluorescence,TUNEL,DHE/Figure 6A and 6B-TUNEL/Figure 6A/Sham/Merge.tif]

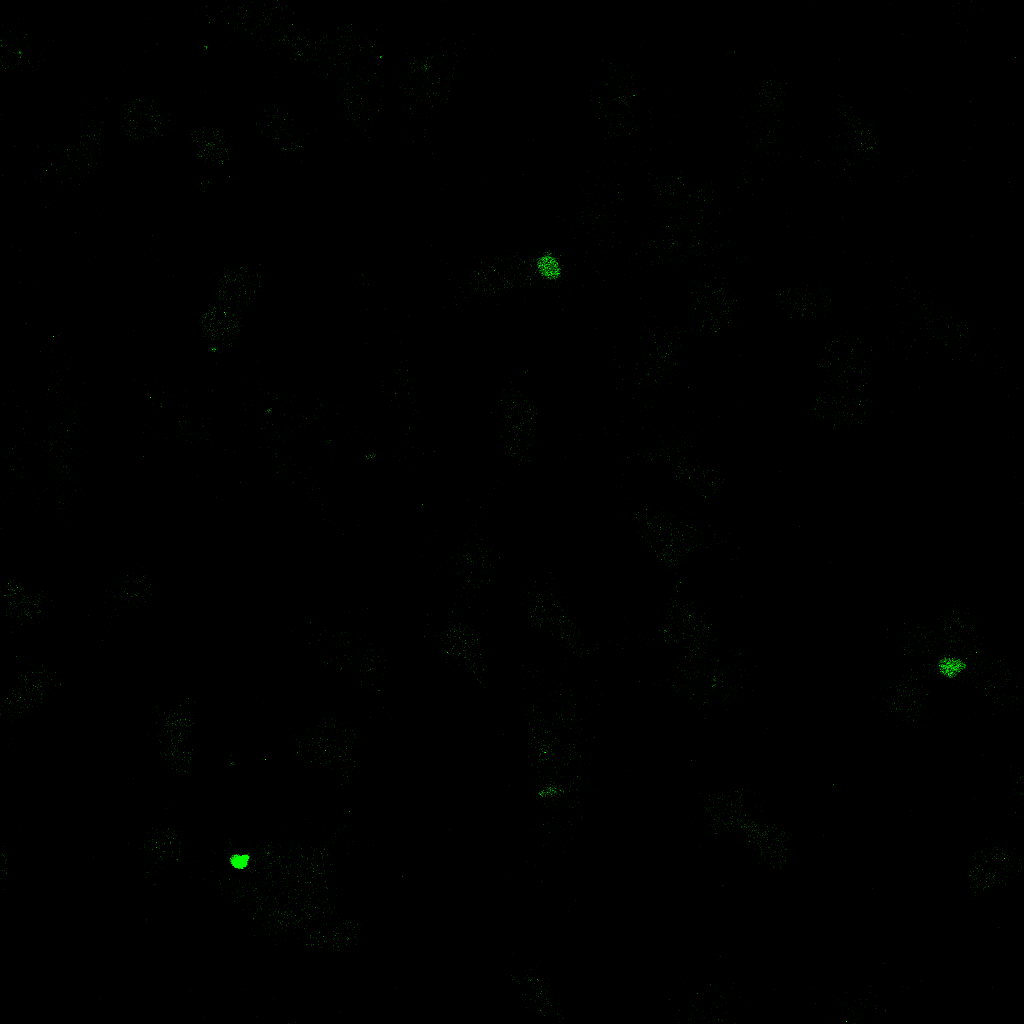

Supplement: Supplementary file 2 [file DataSheet3.ZIP › Raw data-Immunofluorescence,TUNEL,DHE/Figure 6A and 6B-TUNEL/Figure 6A/Sham/TUNEL.tif]

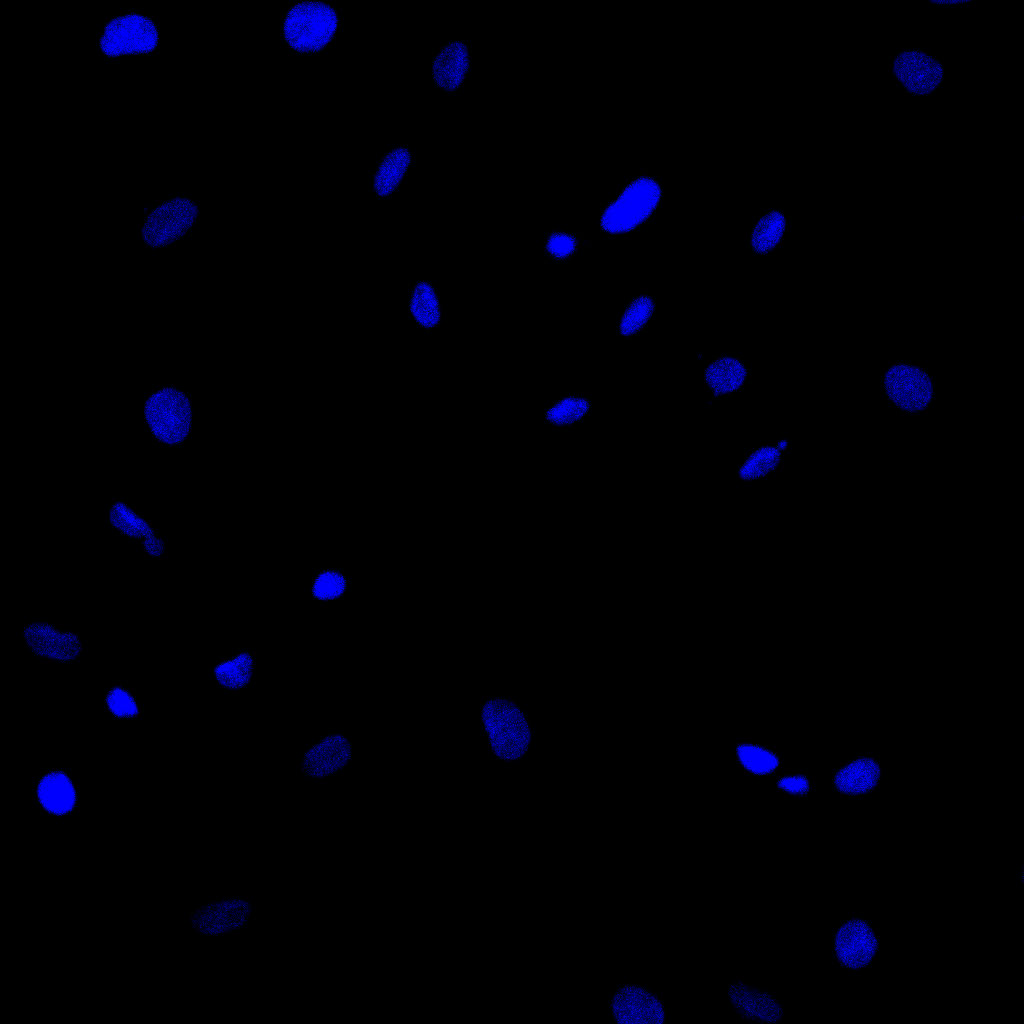

Supplement: Supplementary file 2 [file DataSheet3.ZIP › Raw data-Immunofluorescence,TUNEL,DHE/Figure 6A and 6B-TUNEL/Figure 6A/UA/DAPI.tif]

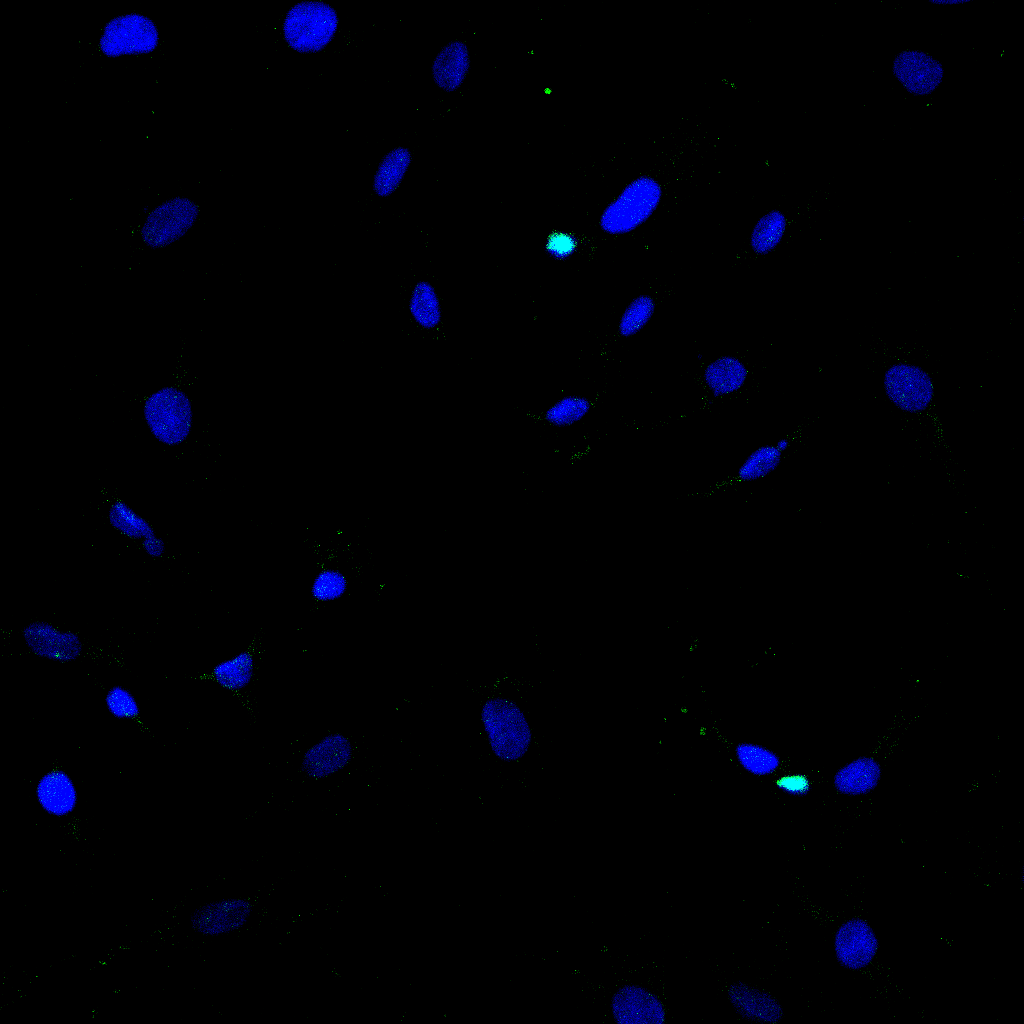

Supplement: Supplementary file 2 [file DataSheet3.ZIP › Raw data-Immunofluorescence,TUNEL,DHE/Figure 6A and 6B-TUNEL/Figure 6A/UA/Merge.tif]

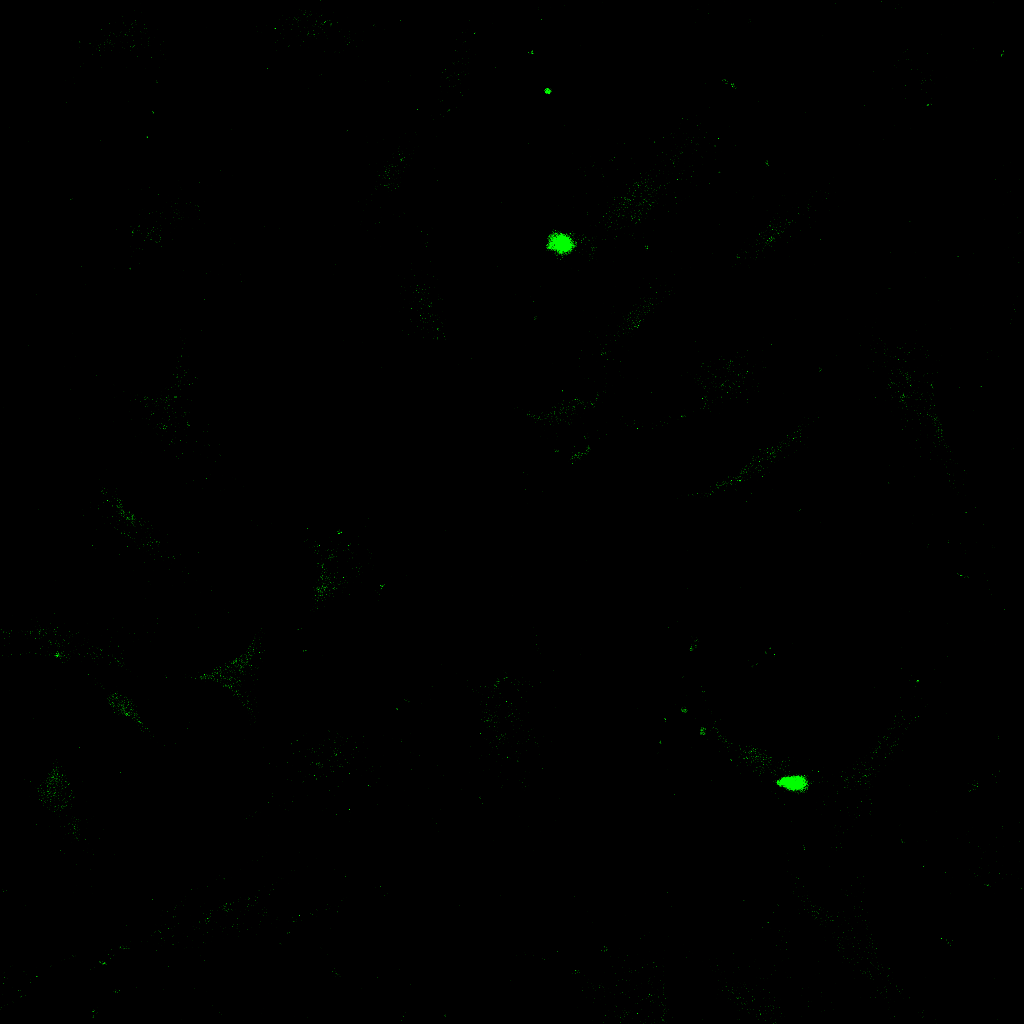

Supplement: Supplementary file 2 [file DataSheet3.ZIP › Raw data-Immunofluorescence,TUNEL,DHE/Figure 6A and 6B-TUNEL/Figure 6A/UA/TUNEL.tif]

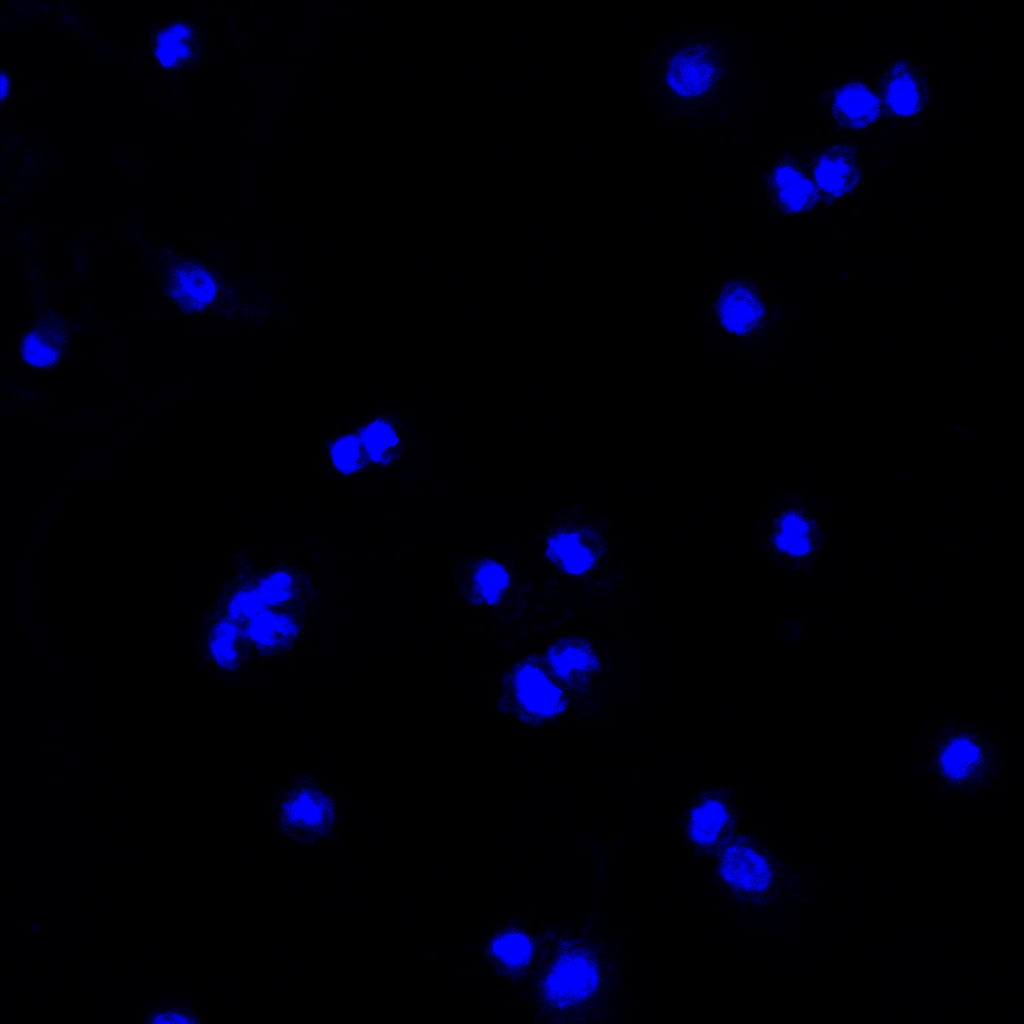

Supplement: Supplementary file 2 [file DataSheet3.ZIP › Raw data-Immunofluorescence,TUNEL,DHE/Figure 6C and 6D-DHE/Figure 6C/IL-6/DAPI.tif]

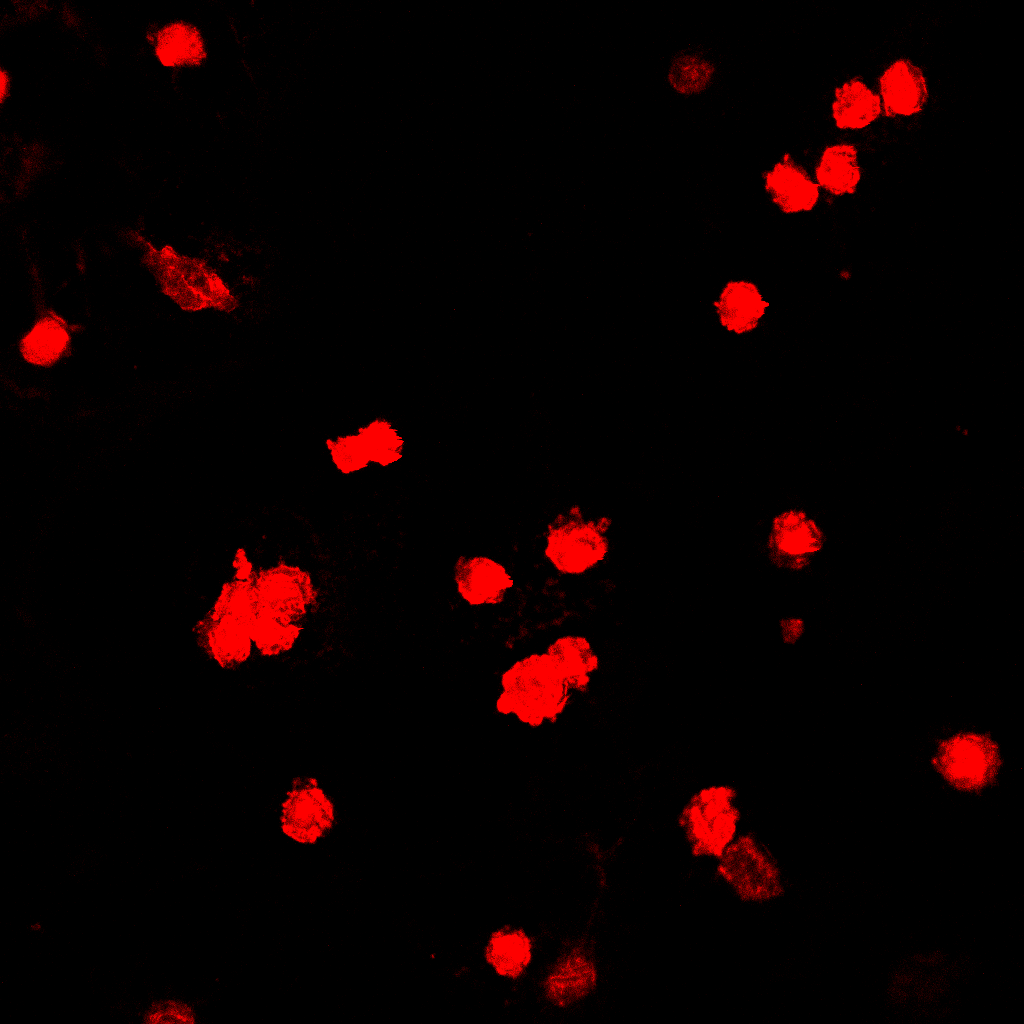

Supplement: Supplementary file 2 [file DataSheet3.ZIP › Raw data-Immunofluorescence,TUNEL,DHE/Figure 6C and 6D-DHE/Figure 6C/IL-6/DHE staining.tif]

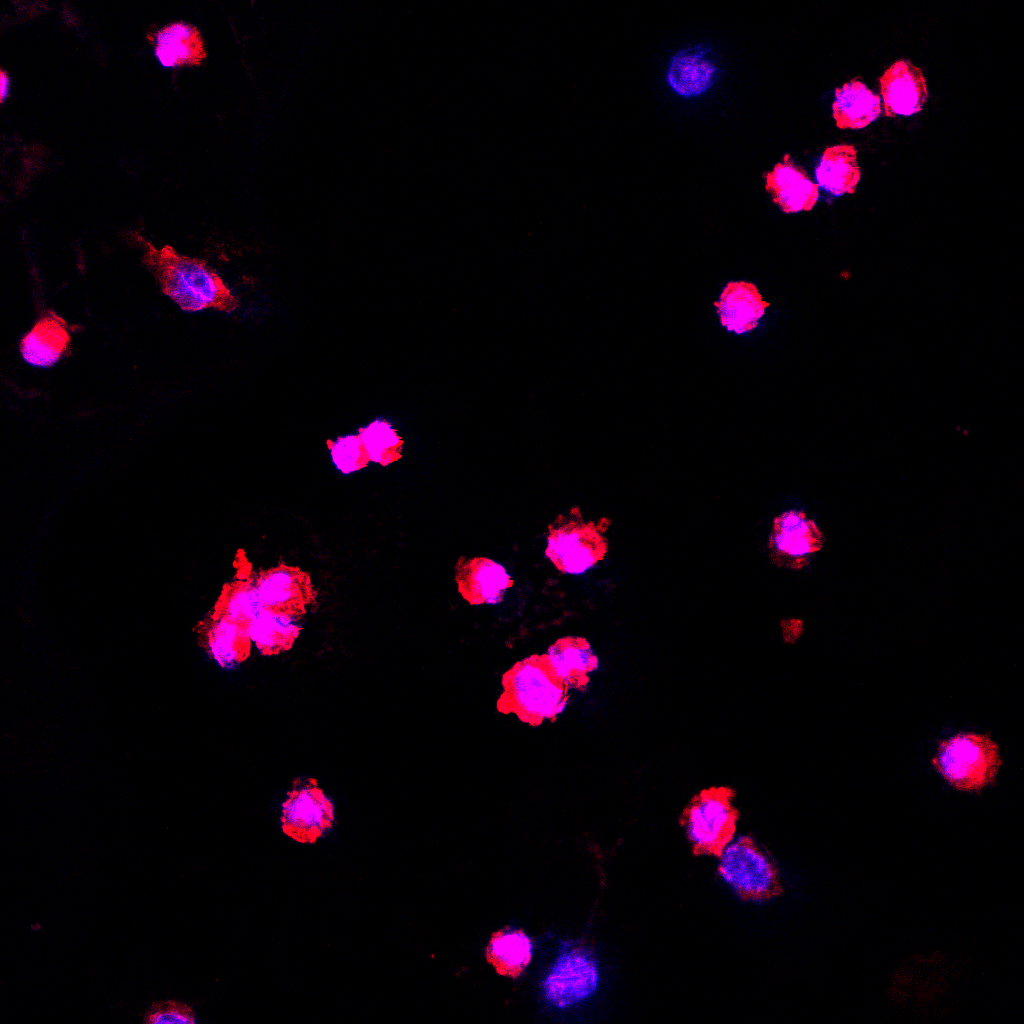

Supplement: Supplementary file 2 [file DataSheet3.ZIP › Raw data-Immunofluorescence,TUNEL,DHE/Figure 6C and 6D-DHE/Figure 6C/IL-6/Merge.tif]

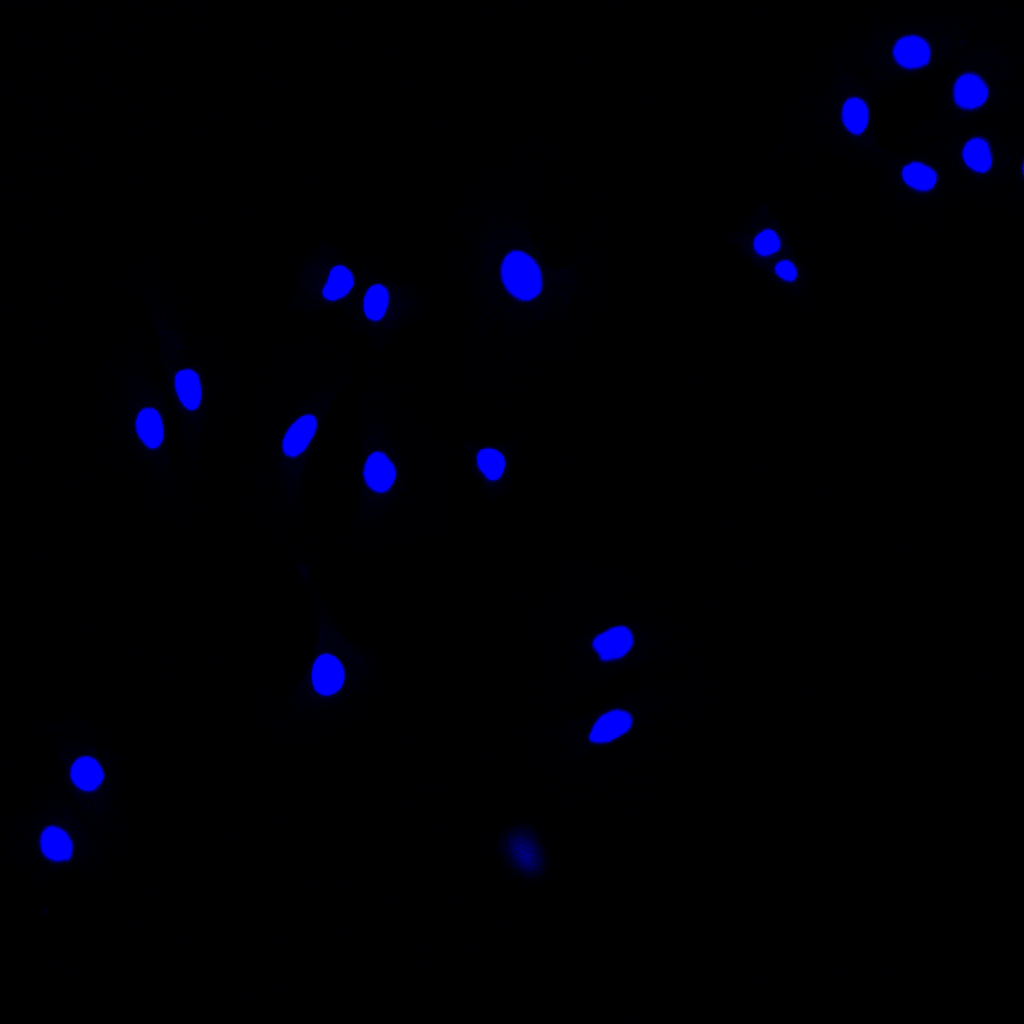

Supplement: Supplementary file 2 [file DataSheet3.ZIP › Raw data-Immunofluorescence,TUNEL,DHE/Figure 6C and 6D-DHE/Figure 6C/ML385/DAPI.tif]

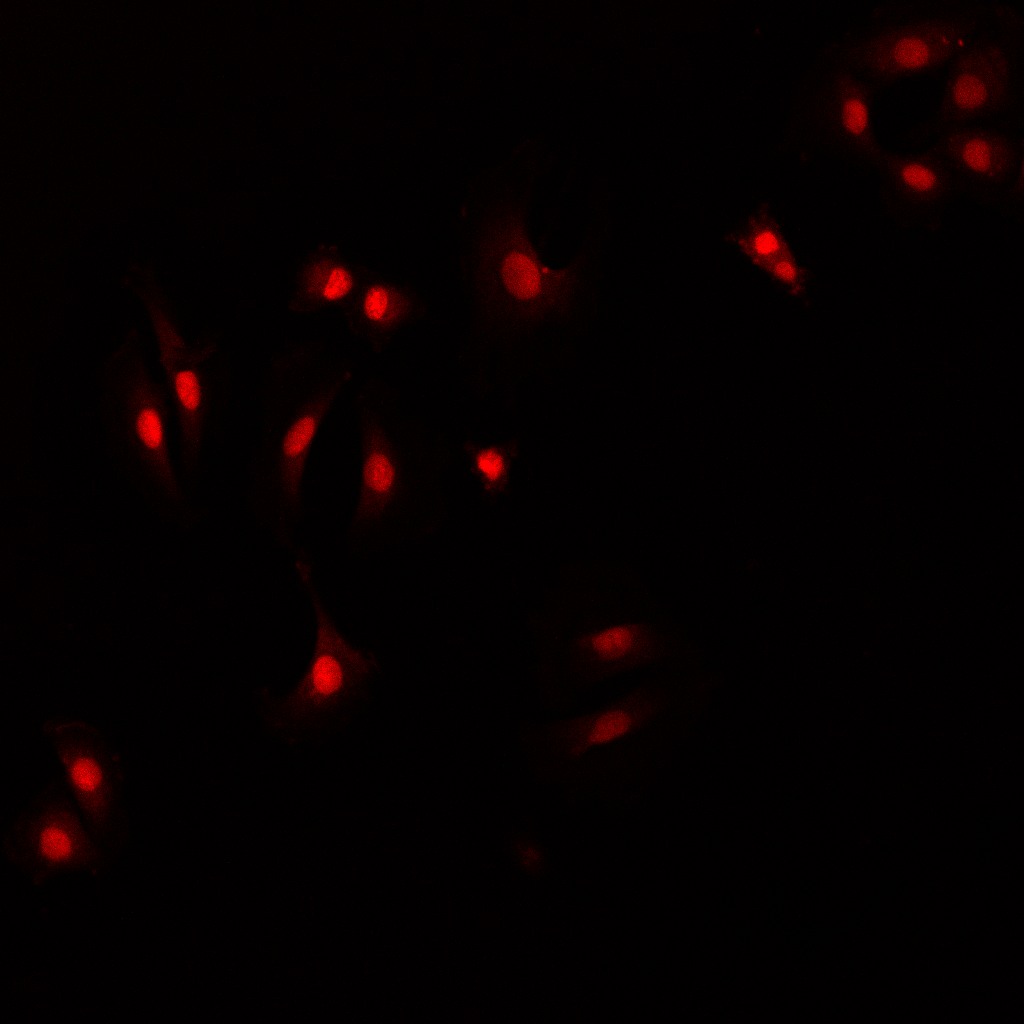

Supplement: Supplementary file 2 [file DataSheet3.ZIP › Raw data-Immunofluorescence,TUNEL,DHE/Figure 6C and 6D-DHE/Figure 6C/ML385/DHE staining.tif]

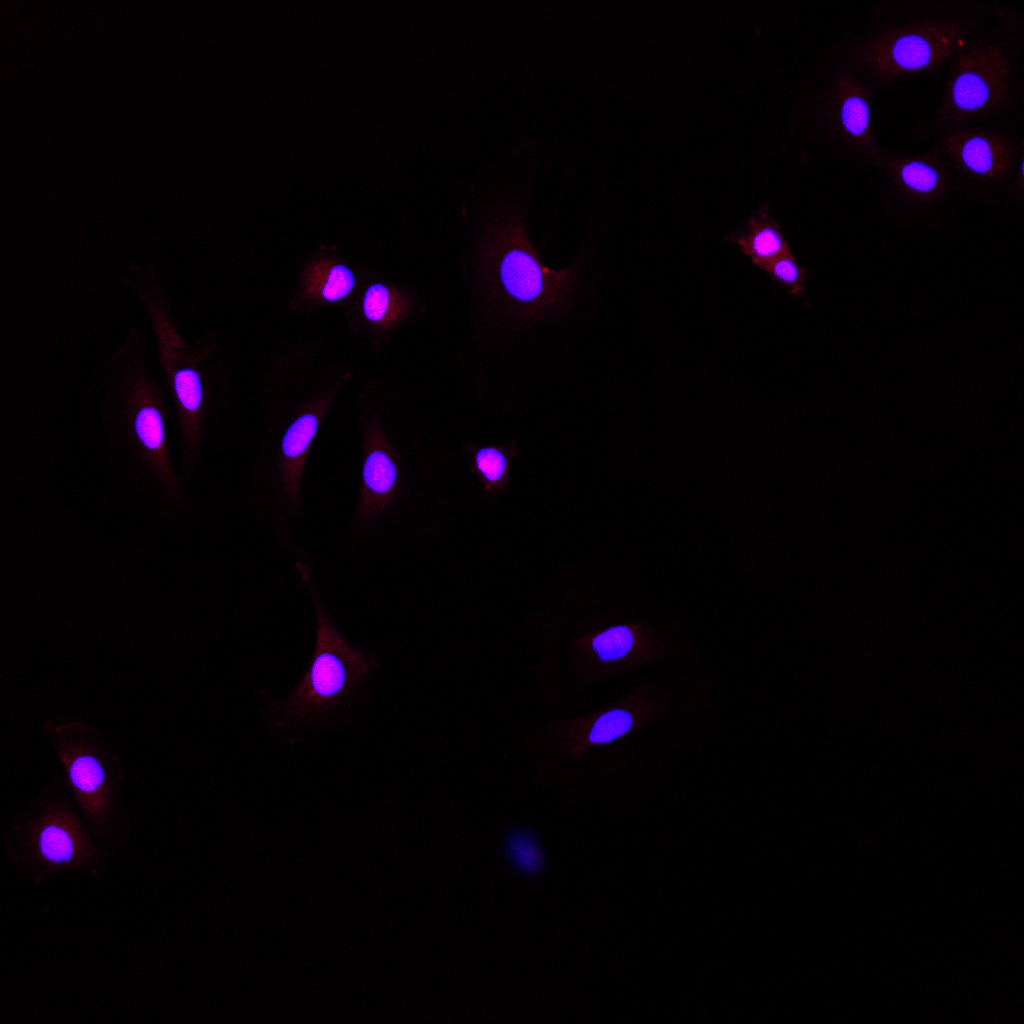

Supplement: Supplementary file 2 [file DataSheet3.ZIP › Raw data-Immunofluorescence,TUNEL,DHE/Figure 6C and 6D-DHE/Figure 6C/ML385/Merge.tif]

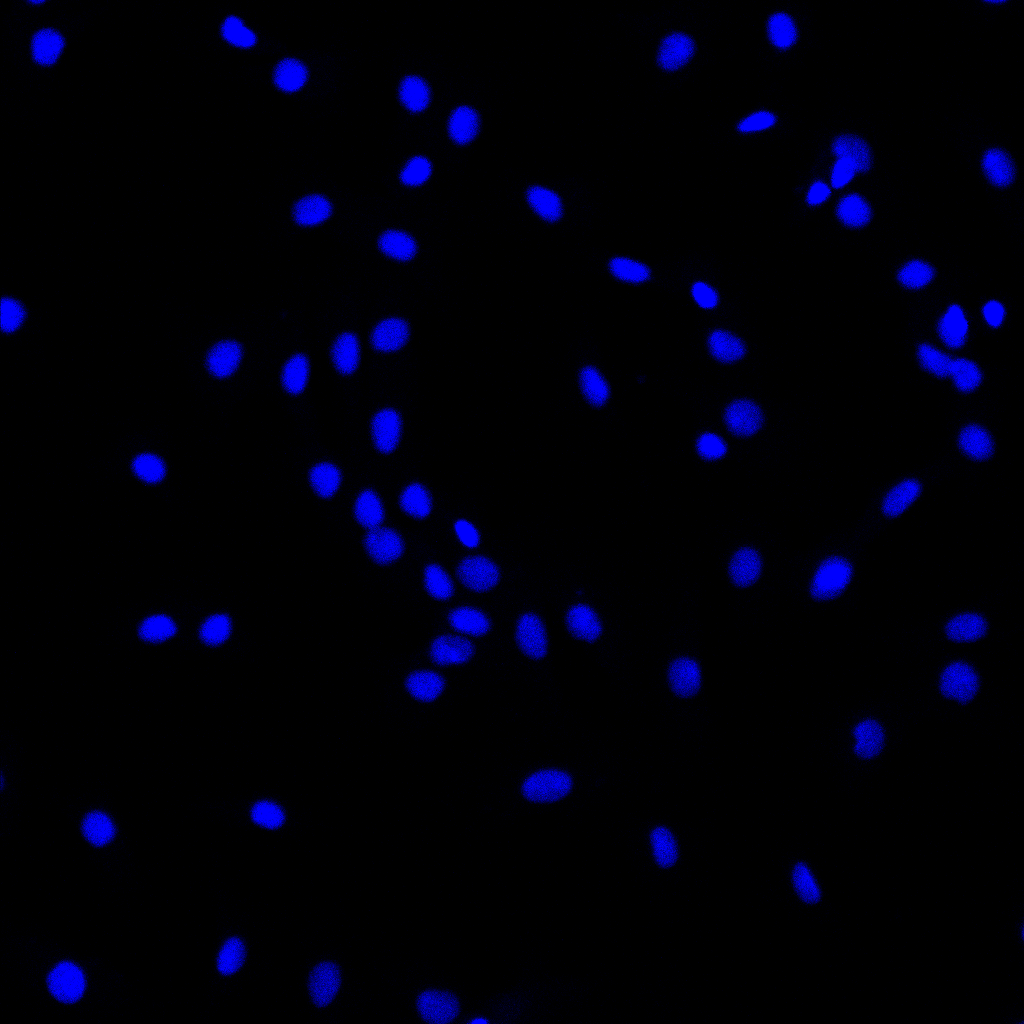

Supplement: Supplementary file 2 [file DataSheet3.ZIP › Raw data-Immunofluorescence,TUNEL,DHE/Figure 6C and 6D-DHE/Figure 6C/Sham/DAPI.tif]

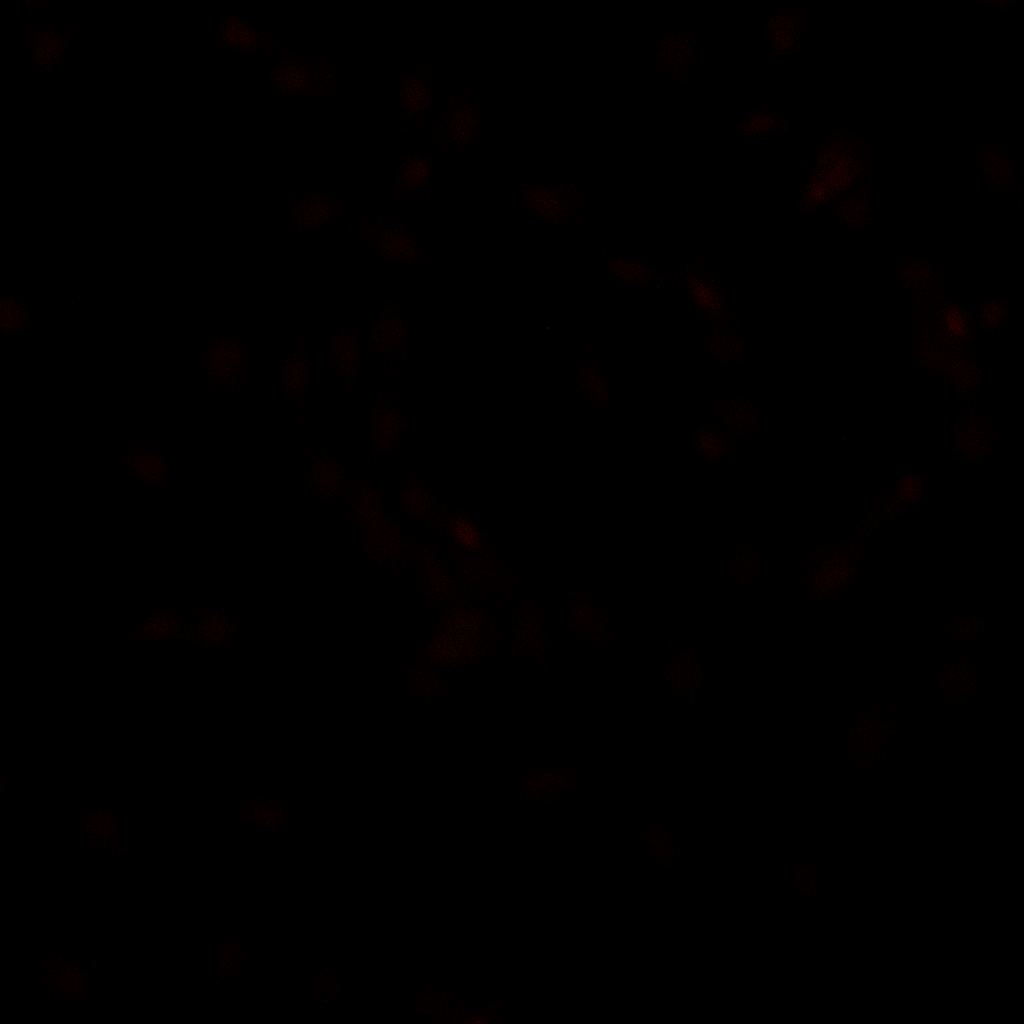

Supplement: Supplementary file 2 [file DataSheet3.ZIP › Raw data-Immunofluorescence,TUNEL,DHE/Figure 6C and 6D-DHE/Figure 6C/Sham/DHE staining.tif]

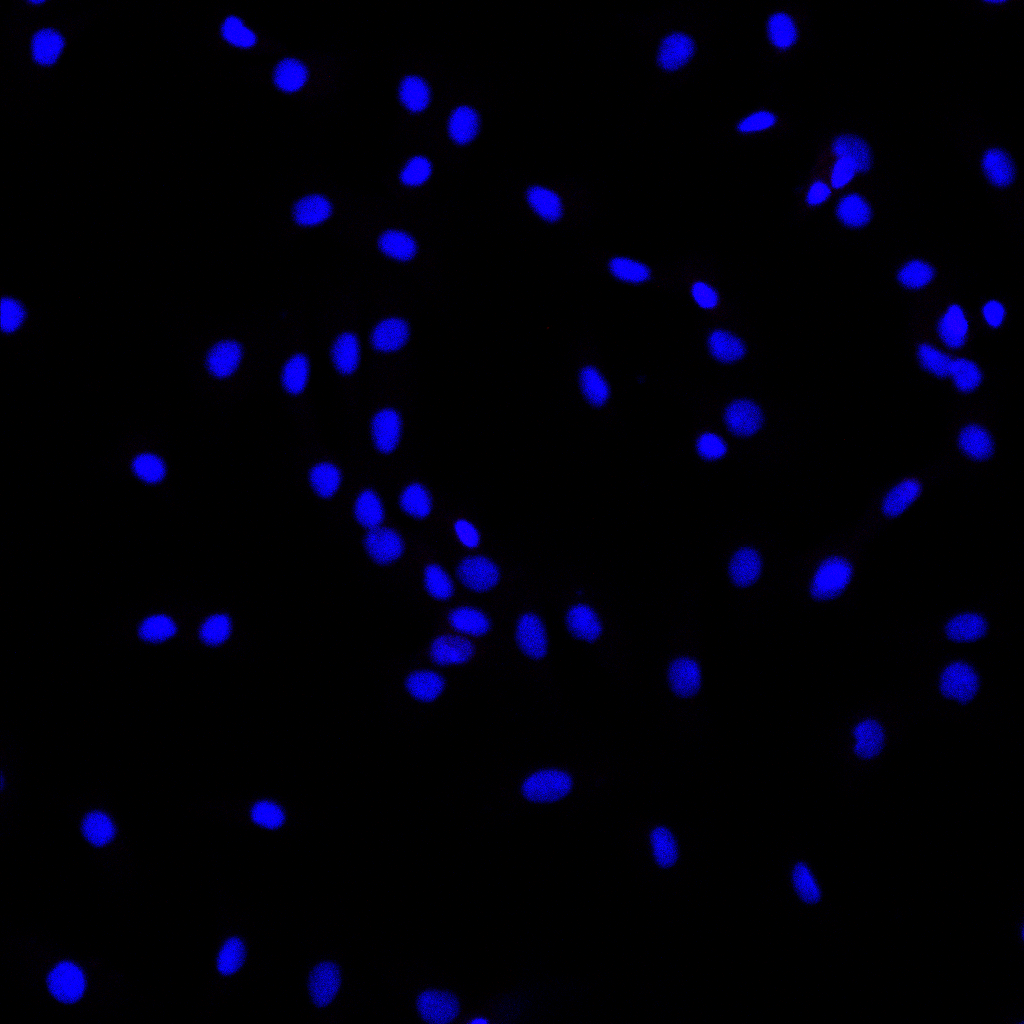

Supplement: Supplementary file 2 [file DataSheet3.ZIP › Raw data-Immunofluorescence,TUNEL,DHE/Figure 6C and 6D-DHE/Figure 6C/Sham/Merge.tif]

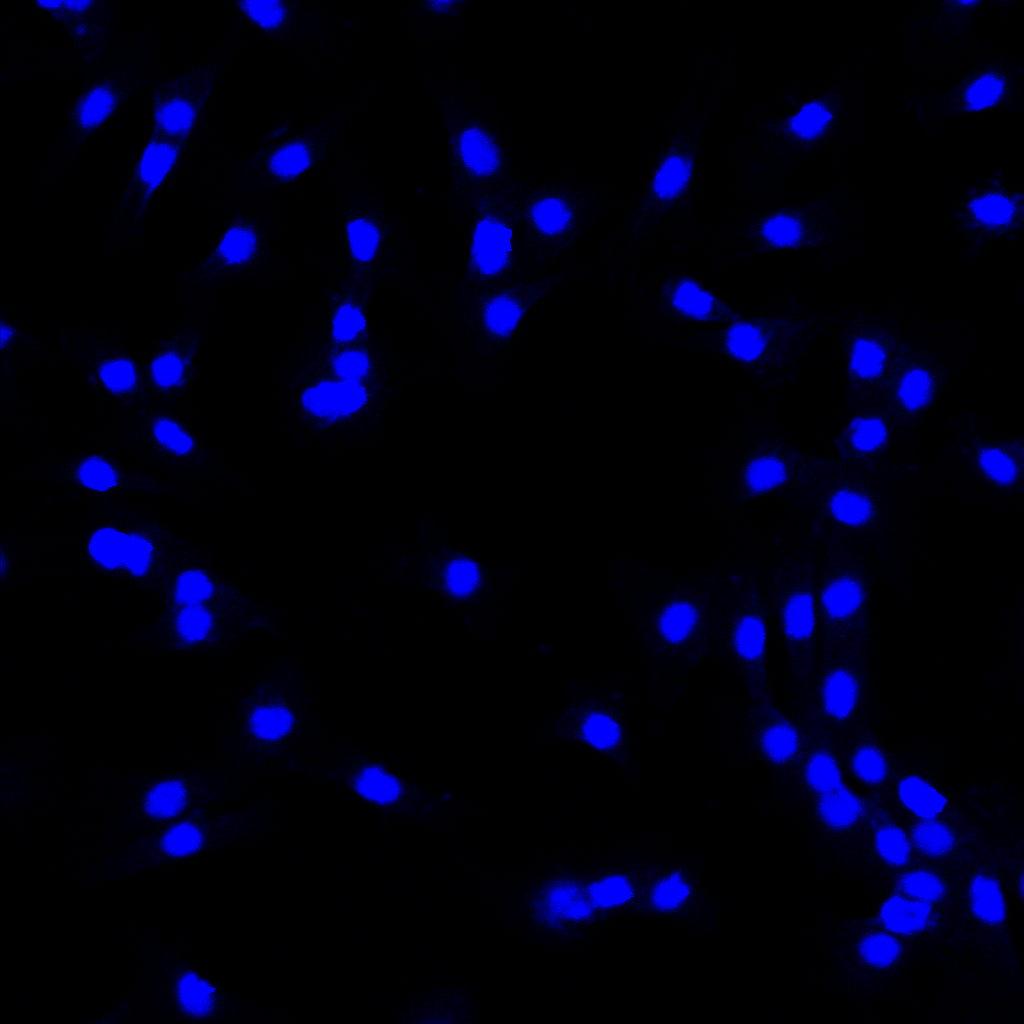

Supplement: Supplementary file 2 [file DataSheet3.ZIP › Raw data-Immunofluorescence,TUNEL,DHE/Figure 6C and 6D-DHE/Figure 6C/UA/DAPI.tif]

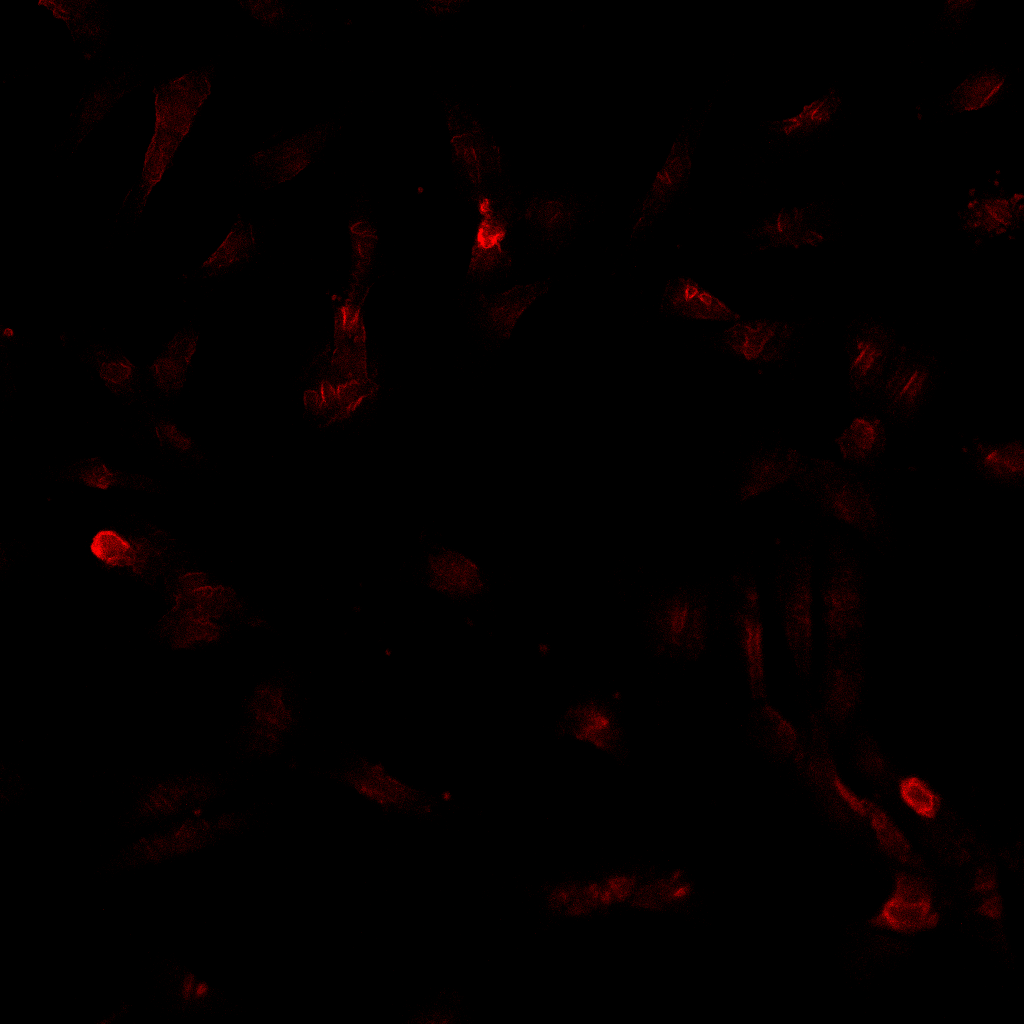

Supplement: Supplementary file 2 [file DataSheet3.ZIP › Raw data-Immunofluorescence,TUNEL,DHE/Figure 6C and 6D-DHE/Figure 6C/UA/DHE staining.tif]

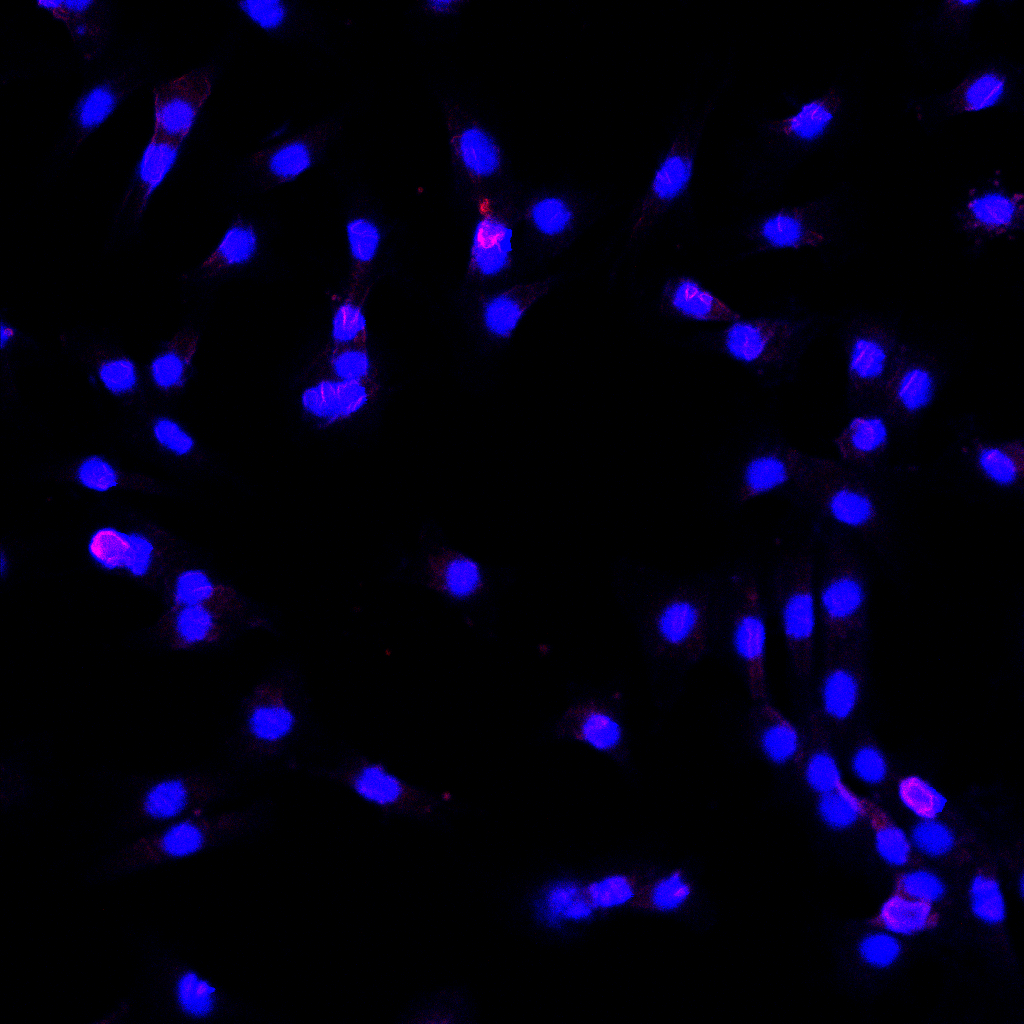

Supplement: Supplementary file 2 [file DataSheet3.ZIP › Raw data-Immunofluorescence,TUNEL,DHE/Figure 6C and 6D-DHE/Figure 6C/UA/Merge.tif]

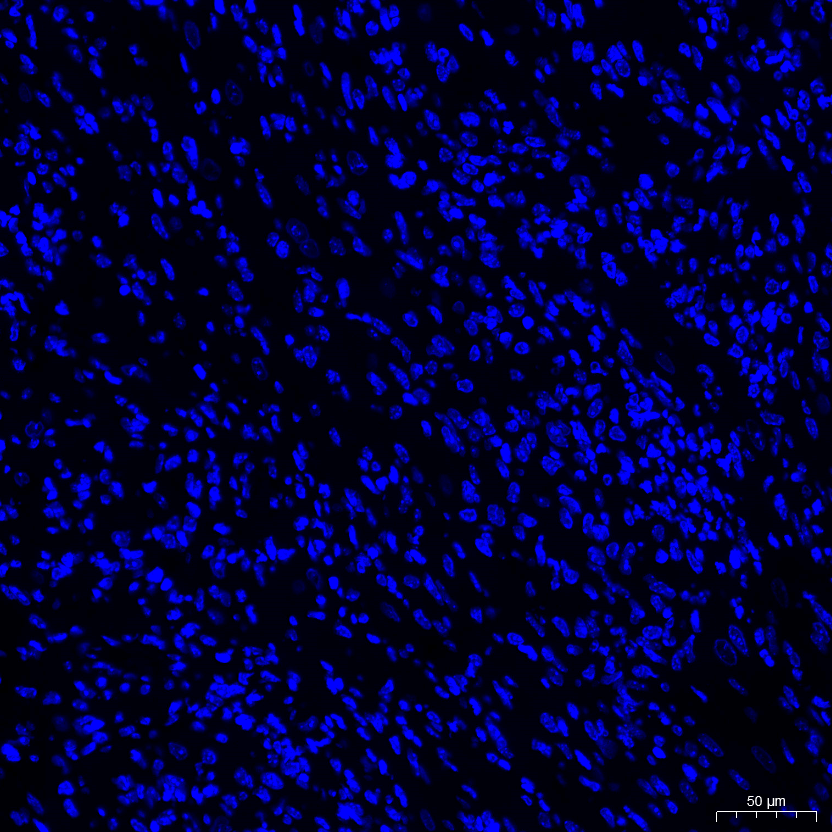

Supplement: Supplementary file 2 [file DataSheet3.ZIP › Raw data-Immunofluorescence,TUNEL,DHE/Figure S1C-a┴-SMA/EAM/DAPI.tif]

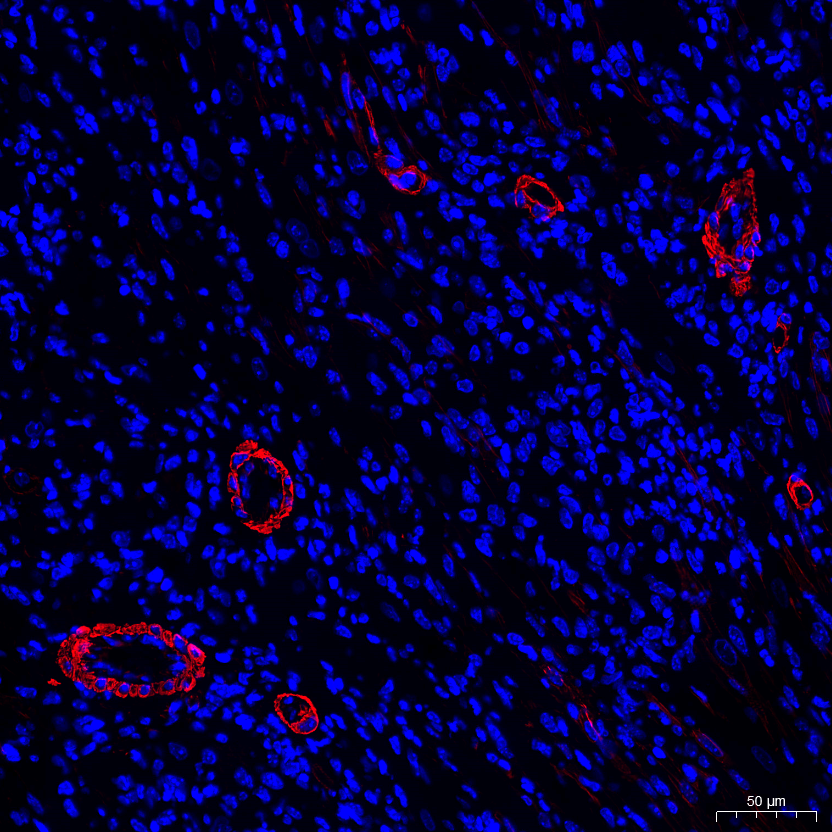

Supplement: Supplementary file 2 [file DataSheet3.ZIP › Raw data-Immunofluorescence,TUNEL,DHE/Figure S1C-a┴-SMA/EAM/Merge.tif]

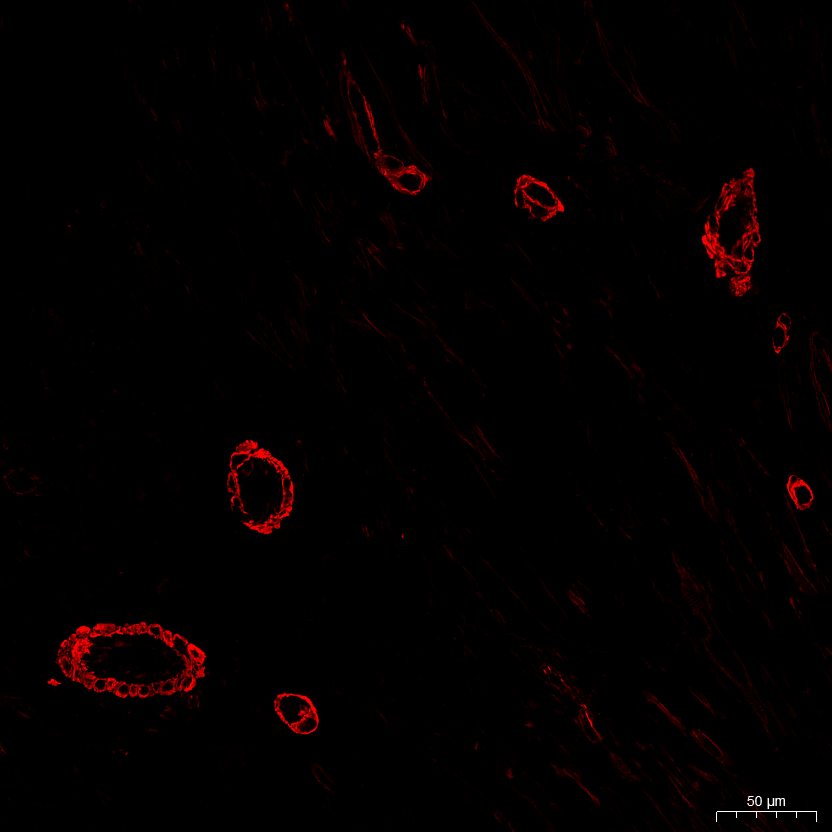

Supplement: Supplementary file 2 [file DataSheet3.ZIP › Raw data-Immunofluorescence,TUNEL,DHE/Figure S1C-a┴-SMA/EAM/a┴-SMA.tif]

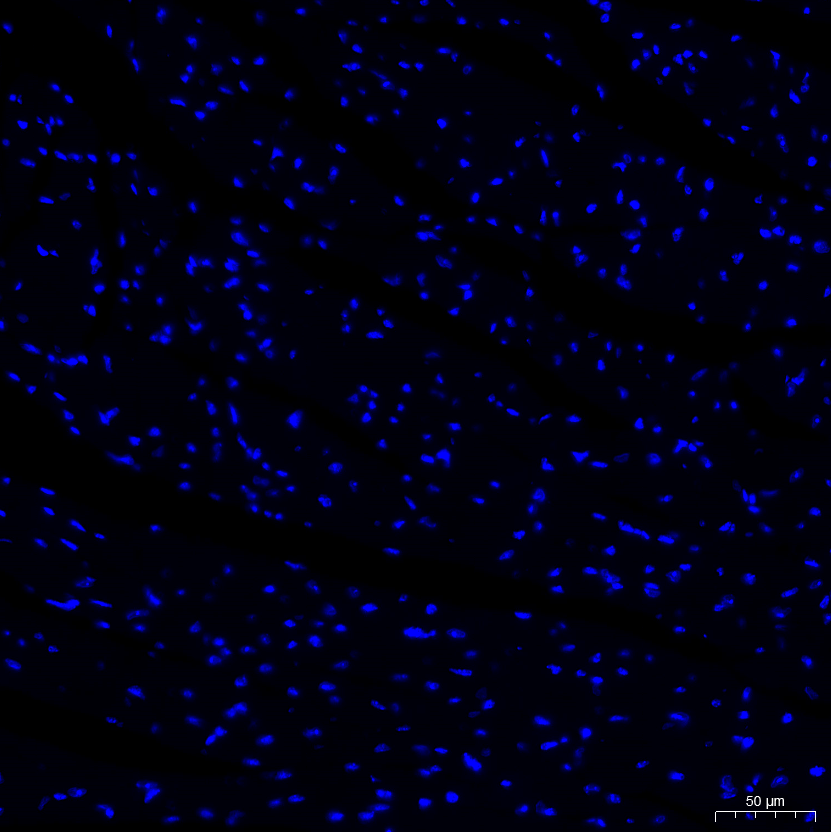

Supplement: Supplementary file 2 [file DataSheet3.ZIP › Raw data-Immunofluorescence,TUNEL,DHE/Figure S1C-a┴-SMA/Sham/DAPI.tif]

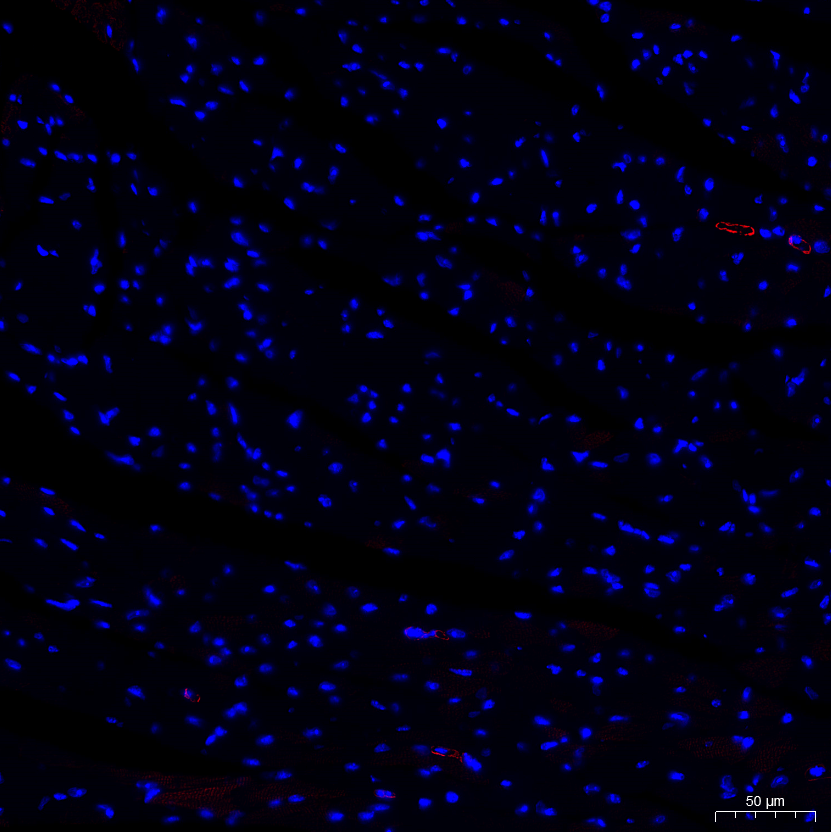

Supplement: Supplementary file 2 [file DataSheet3.ZIP › Raw data-Immunofluorescence,TUNEL,DHE/Figure S1C-a┴-SMA/Sham/Merge.tif]

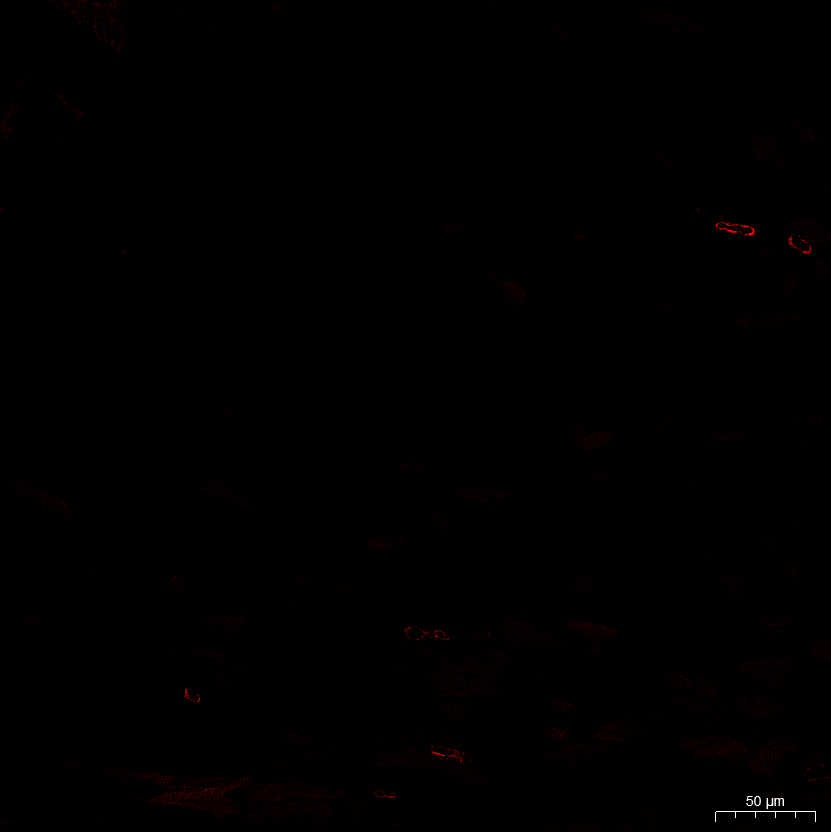

Supplement: Supplementary file 2 [file DataSheet3.ZIP › Raw data-Immunofluorescence,TUNEL,DHE/Figure S1C-a┴-SMA/Sham/a┴-SMA.tif]

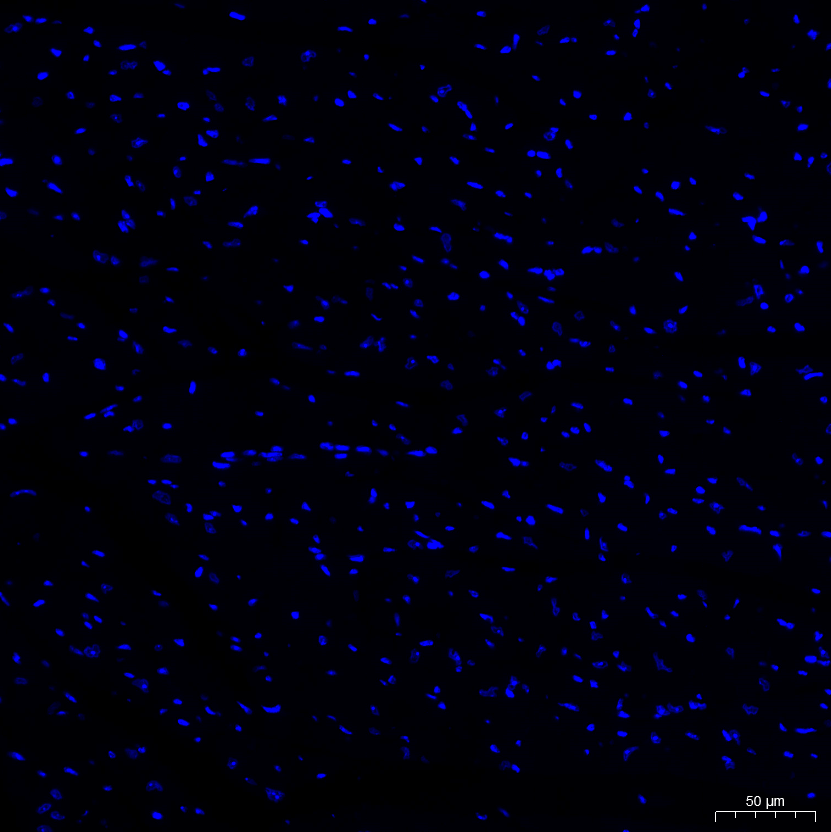

Supplement: Supplementary file 2 [file DataSheet3.ZIP › Raw data-Immunofluorescence,TUNEL,DHE/Figure S1C-a┴-SMA/UA/DAPI.tif]

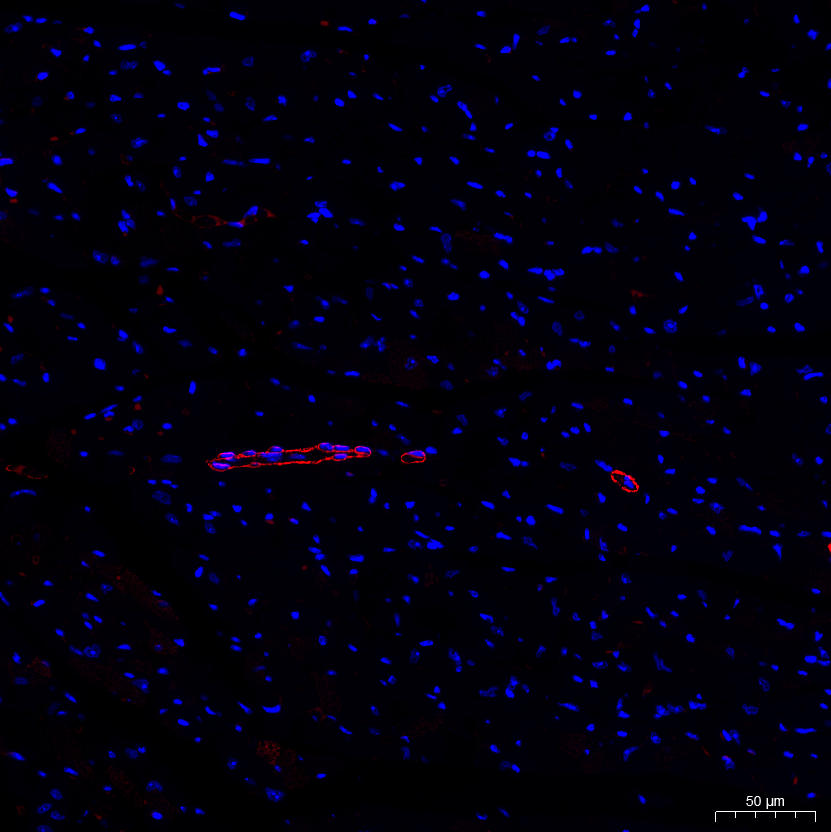

Supplement: Supplementary file 2 [file DataSheet3.ZIP › Raw data-Immunofluorescence,TUNEL,DHE/Figure S1C-a┴-SMA/UA/Merge.tif]

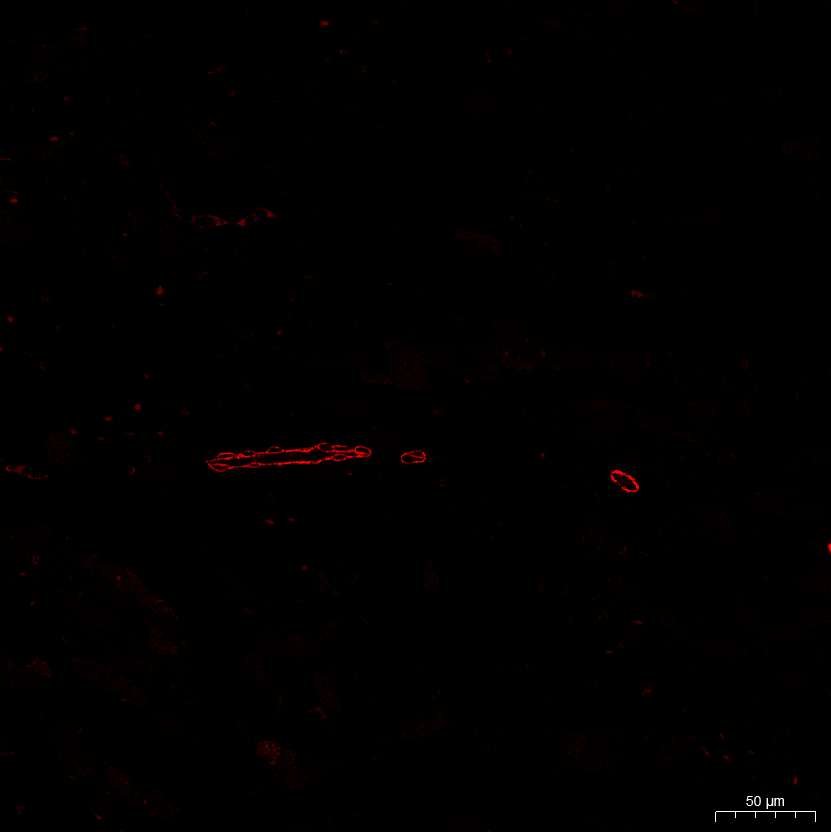

Supplement: Supplementary file 2 [file DataSheet3.ZIP › Raw data-Immunofluorescence,TUNEL,DHE/Figure S1C-a┴-SMA/UA/a┴-SMA.tif]

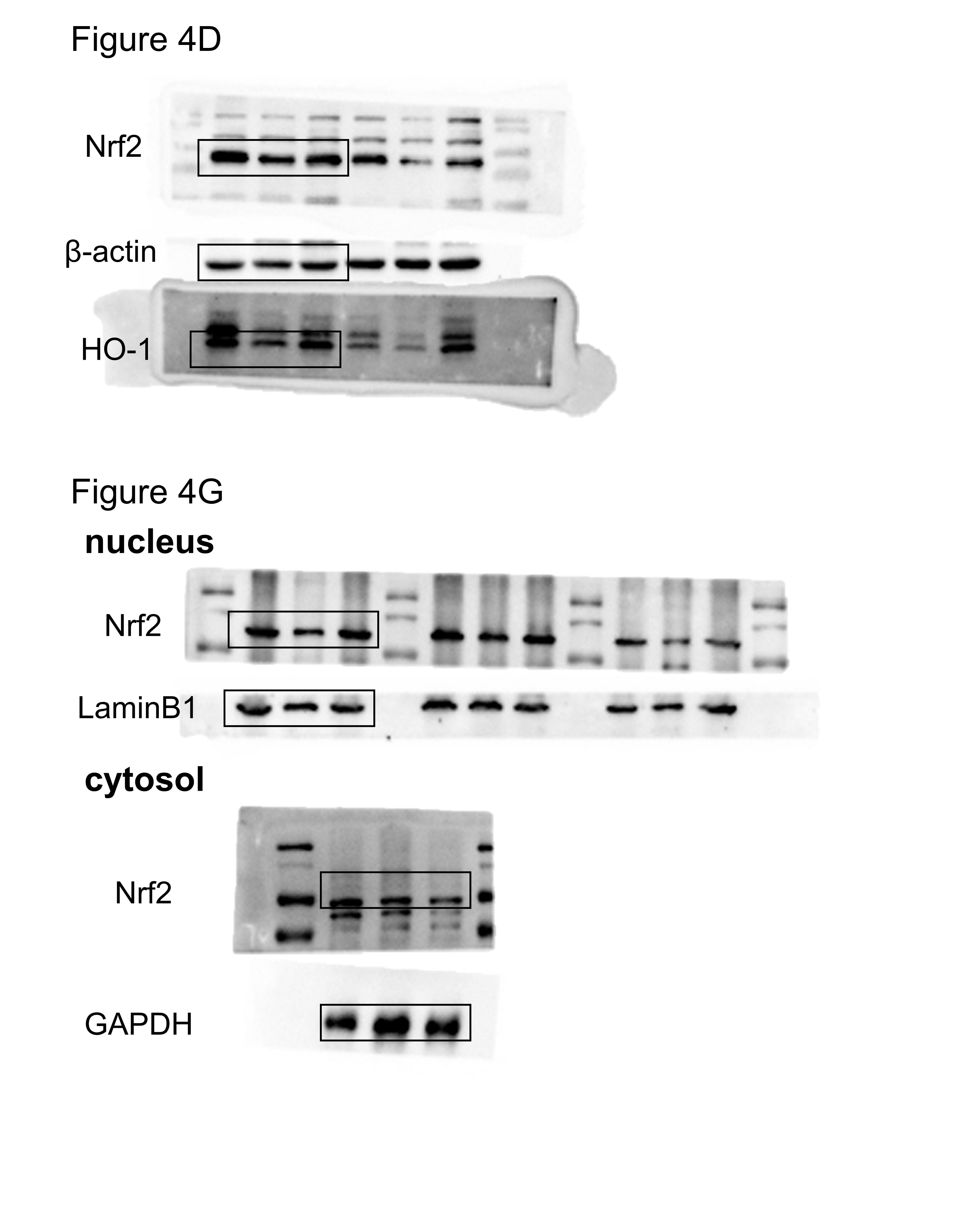

Supplement: Supplementary file 4 [file DataSheet4.ZIP › Raw data-Western Blot/Figure 4D and Figure 4G.tif]

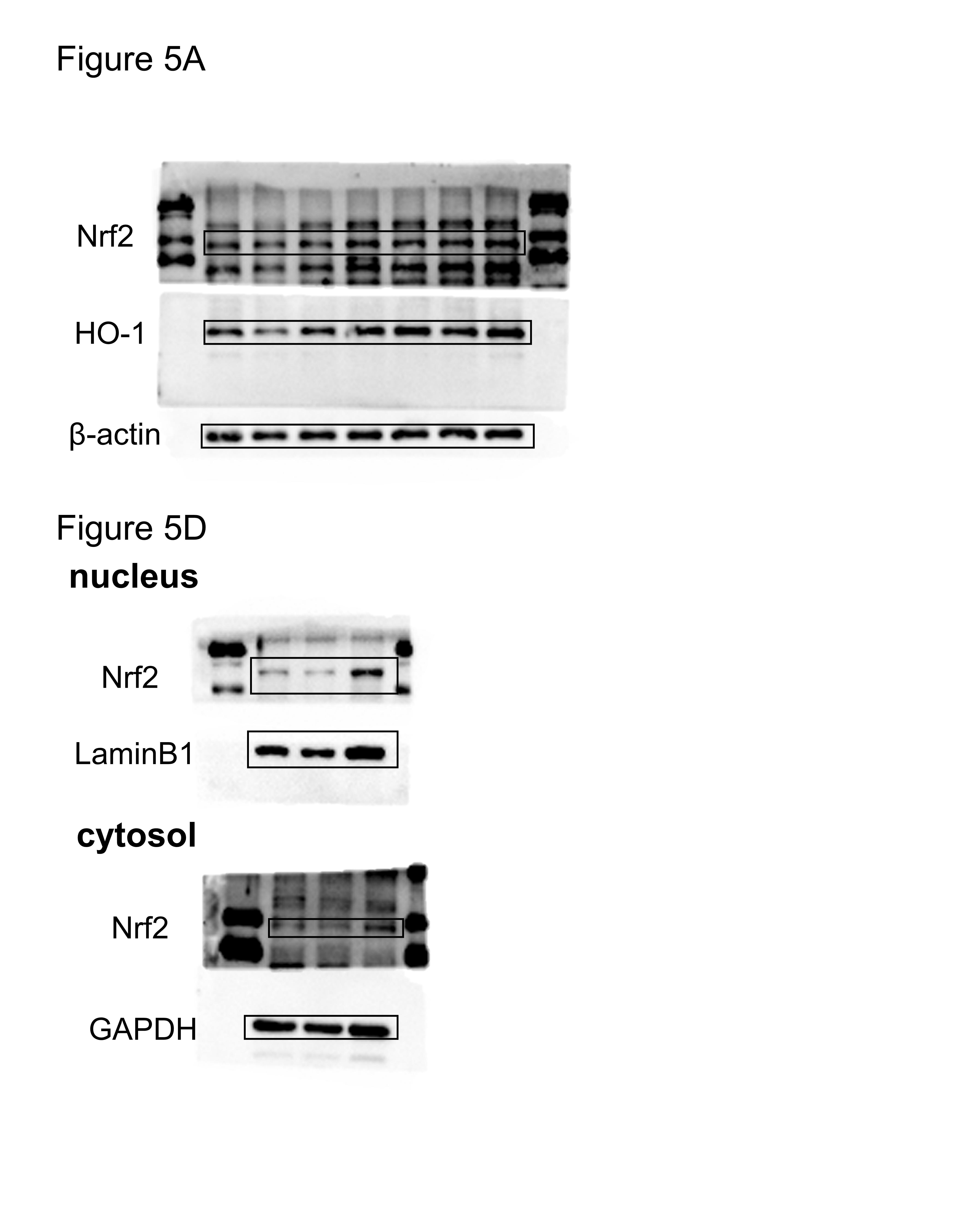

Supplement: Supplementary file 4 [file DataSheet4.ZIP › Raw data-Western Blot/Figure 5A and 5D.tif]

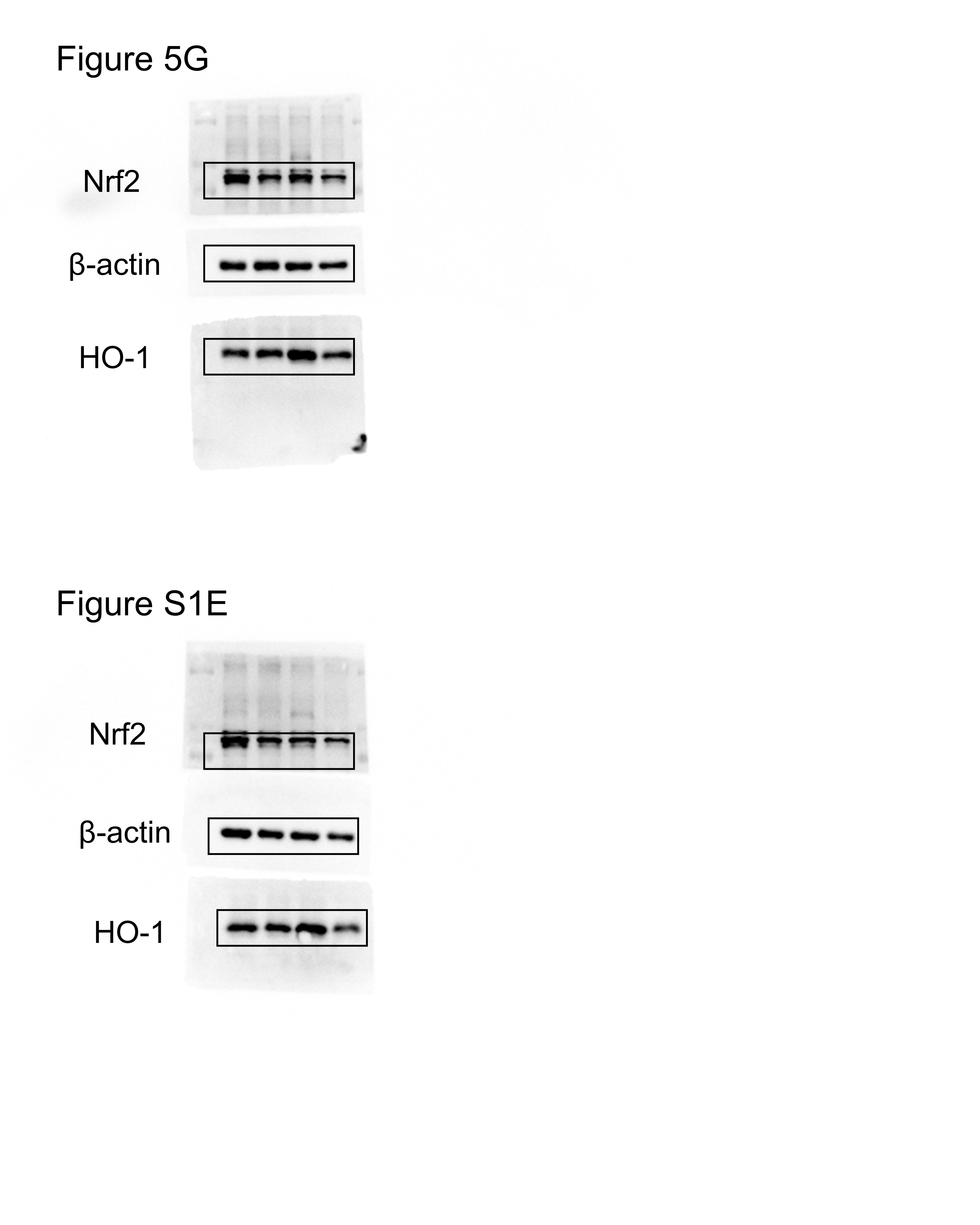

Supplement: Supplementary file 4 [file DataSheet4.ZIP › Raw data-Western Blot/Figure 5G and S1E.tif]

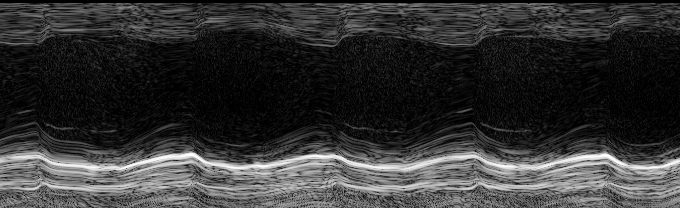

Supplement: Supplementary file 5 [file DataSheet1.ZIP › Raw data-Echocardiography/Figure 2A/EAM.tif]

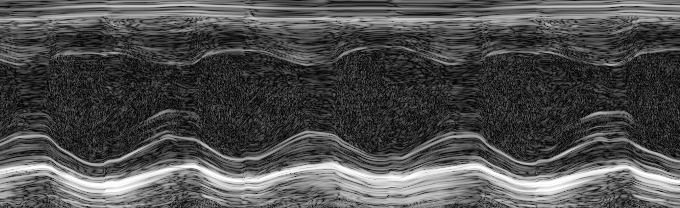

Supplement: Supplementary file 5 [file DataSheet1.ZIP › Raw data-Echocardiography/Figure 2A/Sham.tif]

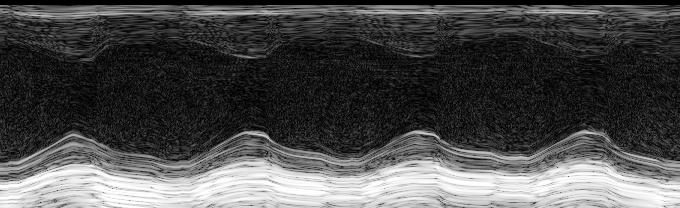

Supplement: Supplementary file 5 [file DataSheet1.ZIP › Raw data-Echocardiography/Figure 2A/UA.tif]

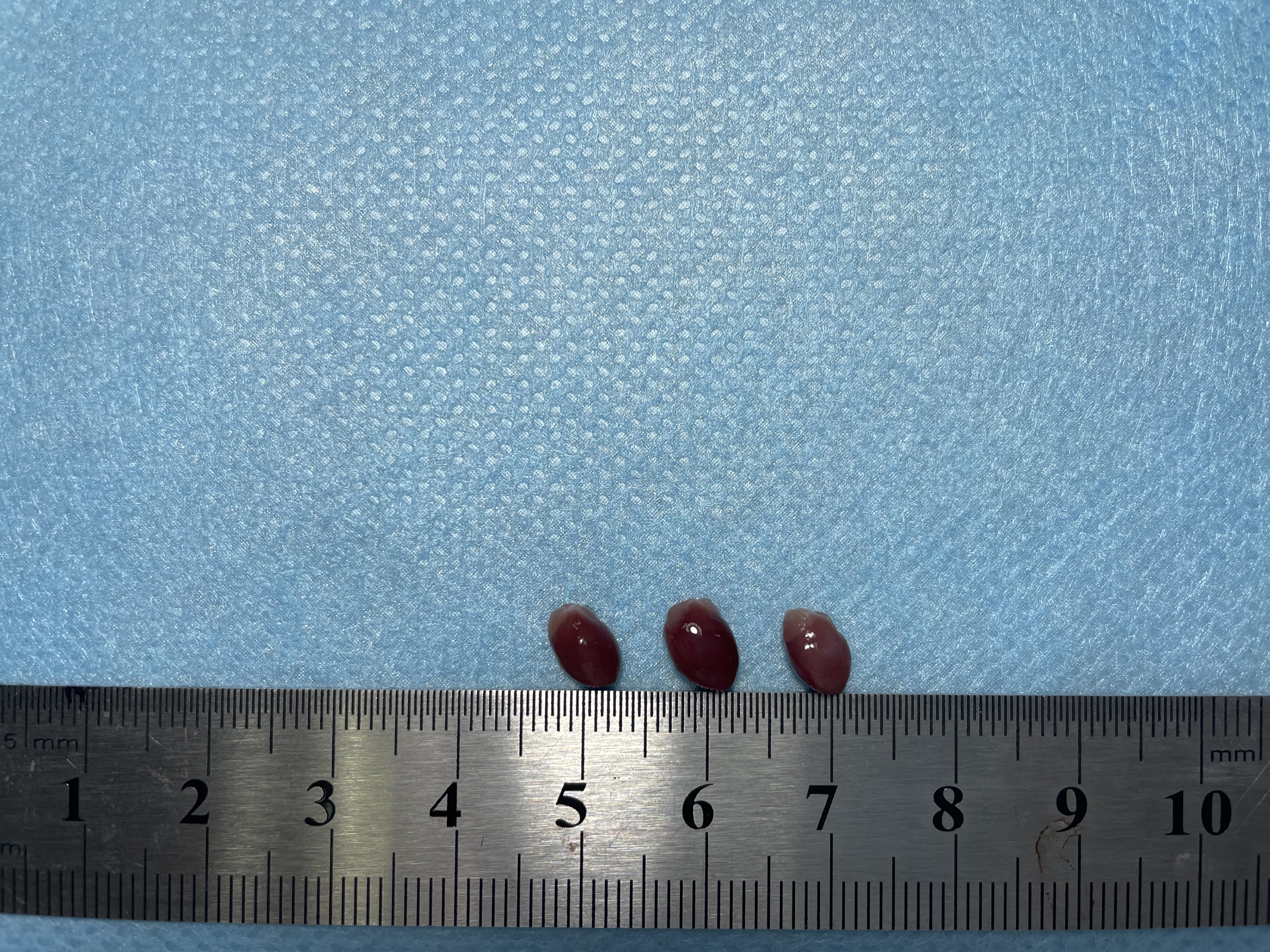

Supplement: Supplementary file 6 [file Image2.JPEG]

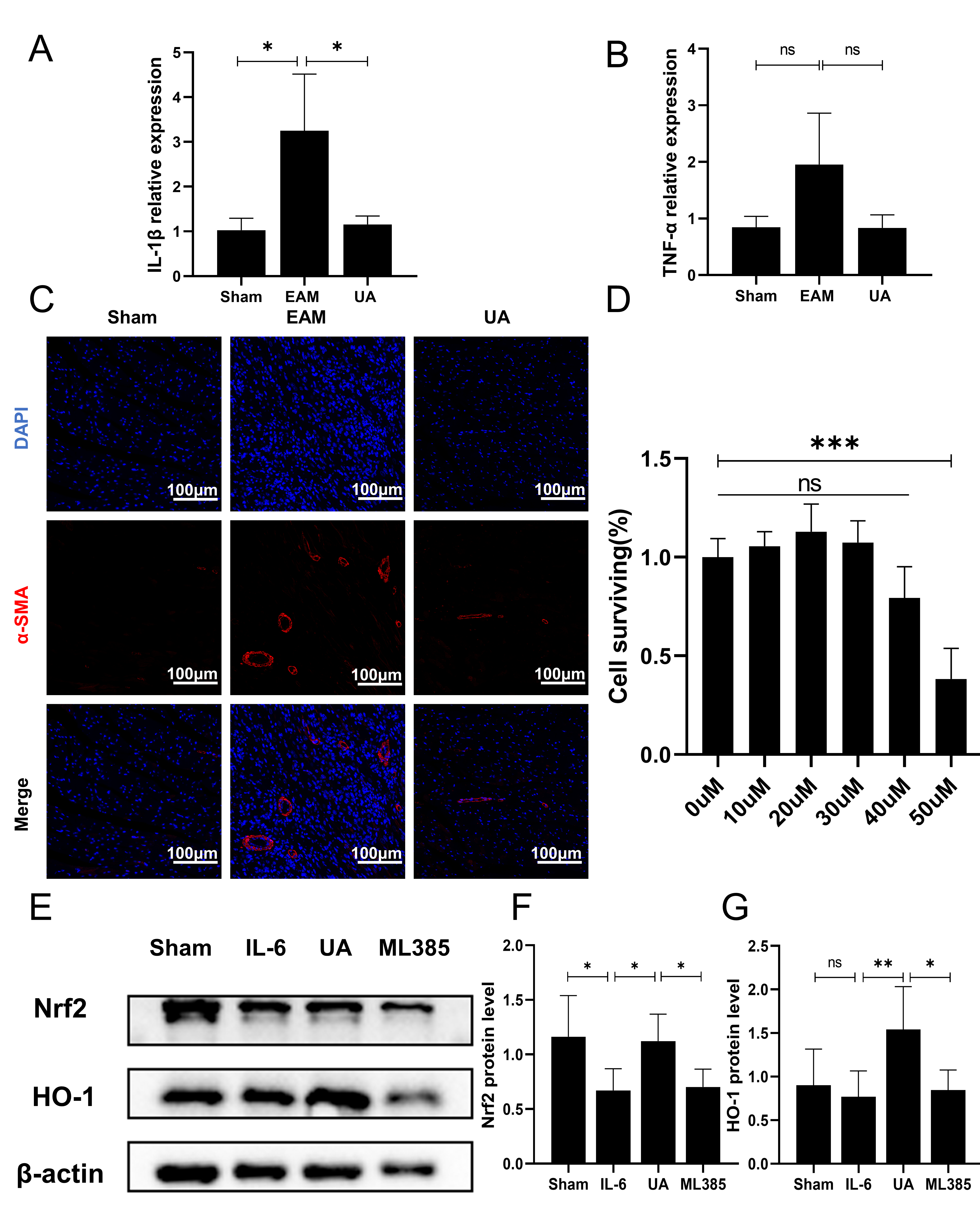

Supplement: Supplementary file 7 [file Image1.TIF]

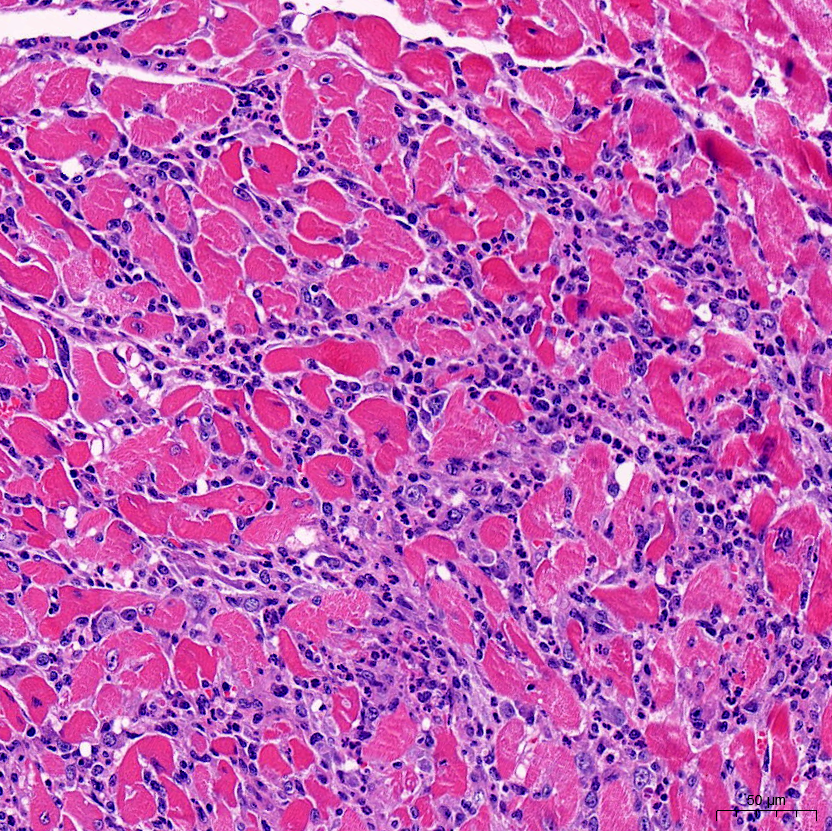

Supplement: Supplementary file 9 [file DataSheet2.ZIP › Raw data-HE, Masson/HE/inset-Scale bars 100 a╠m/EAM.tif]

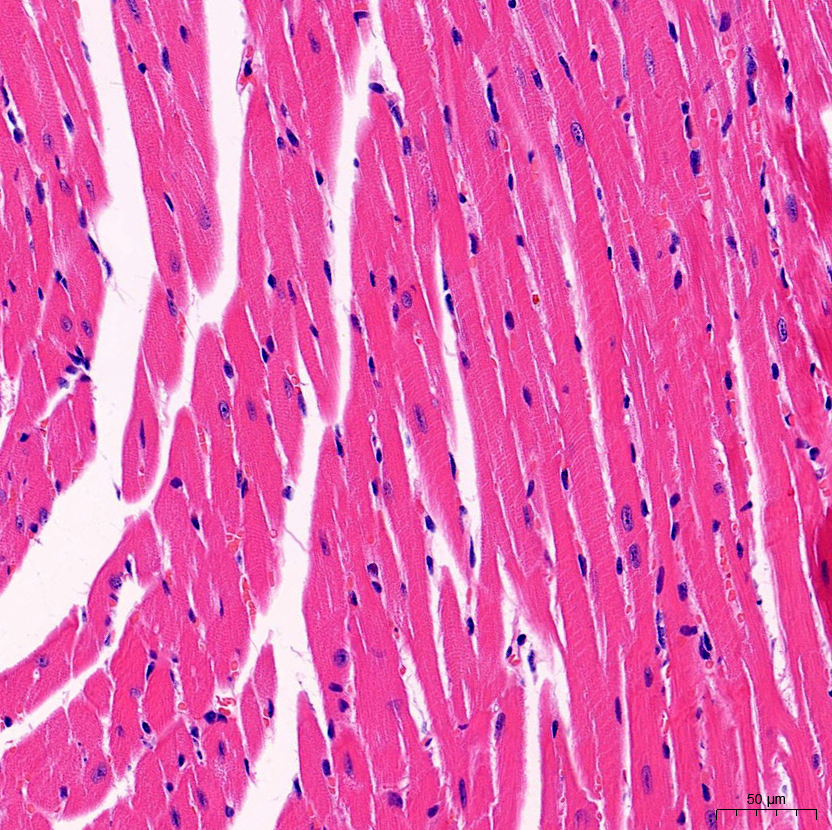

Supplement: Supplementary file 9 [file DataSheet2.ZIP › Raw data-HE, Masson/HE/inset-Scale bars 100 a╠m/Sham.tif]

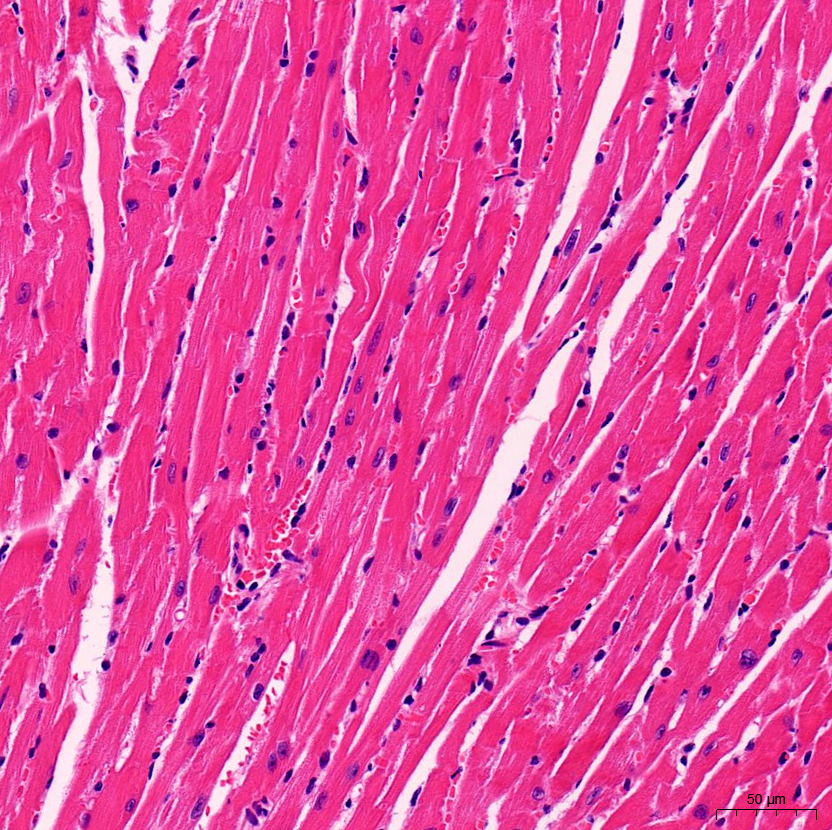

Supplement: Supplementary file 9 [file DataSheet2.ZIP › Raw data-HE, Masson/HE/inset-Scale bars 100 a╠m/UA.tif]

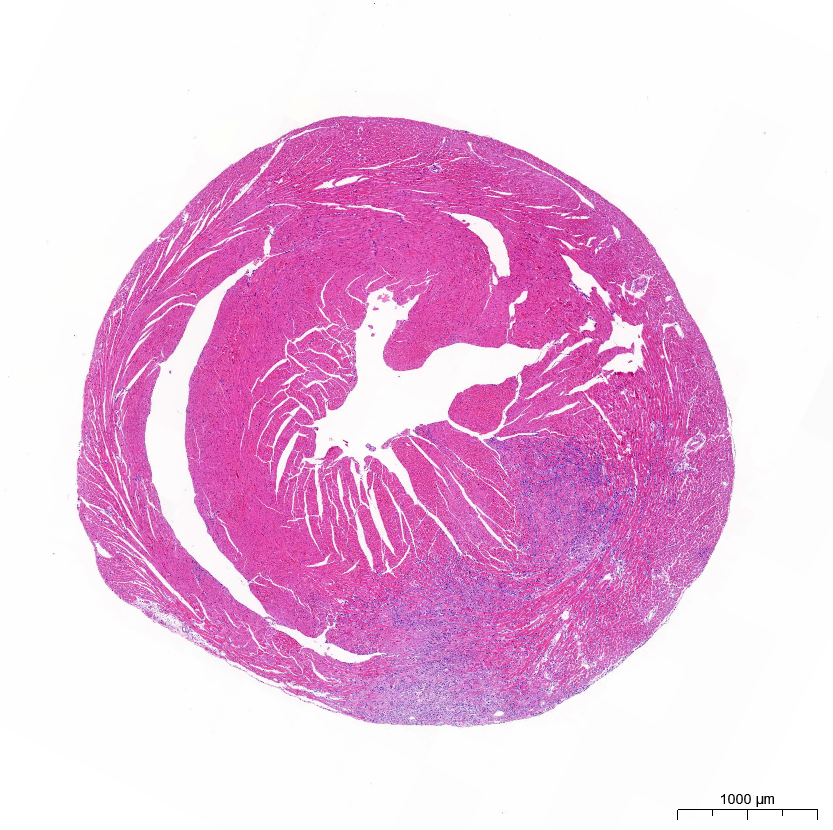

Supplement: Supplementary file 9 [file DataSheet2.ZIP › Raw data-HE, Masson/HE/left-Scale bars 1mm/EAM.tif]

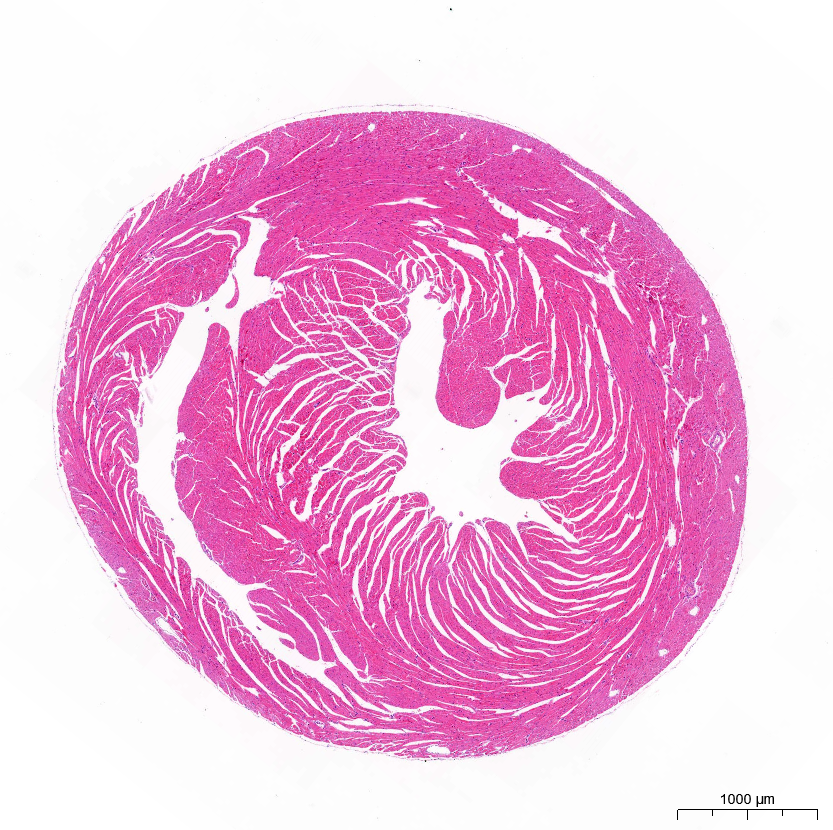

Supplement: Supplementary file 9 [file DataSheet2.ZIP › Raw data-HE, Masson/HE/left-Scale bars 1mm/Sham.tif]

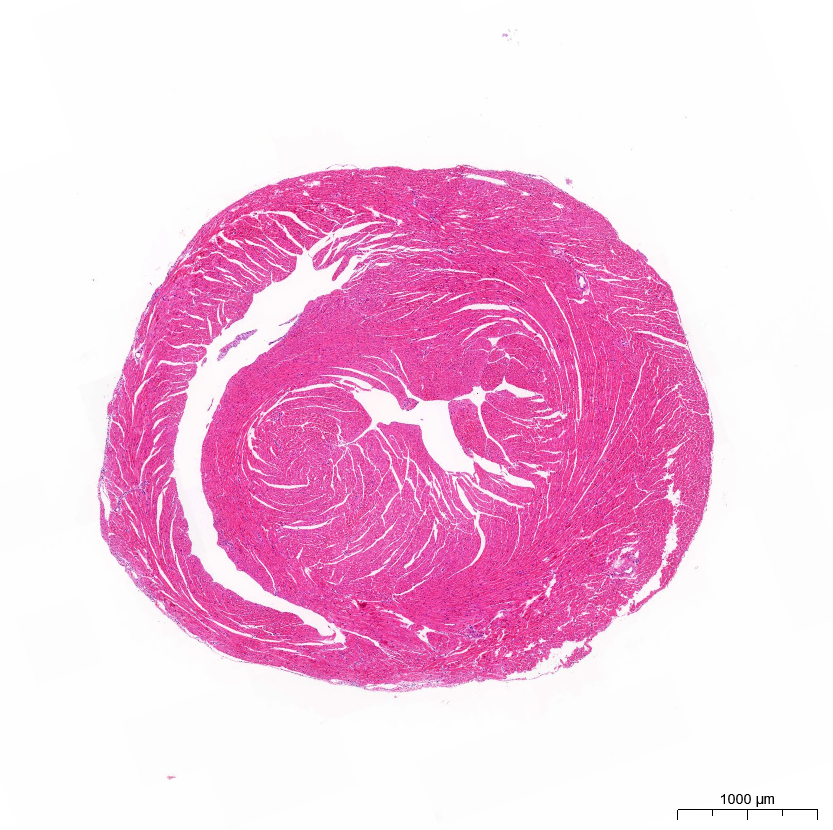

Supplement: Supplementary file 9 [file DataSheet2.ZIP › Raw data-HE, Masson/HE/left-Scale bars 1mm/UA.tif]

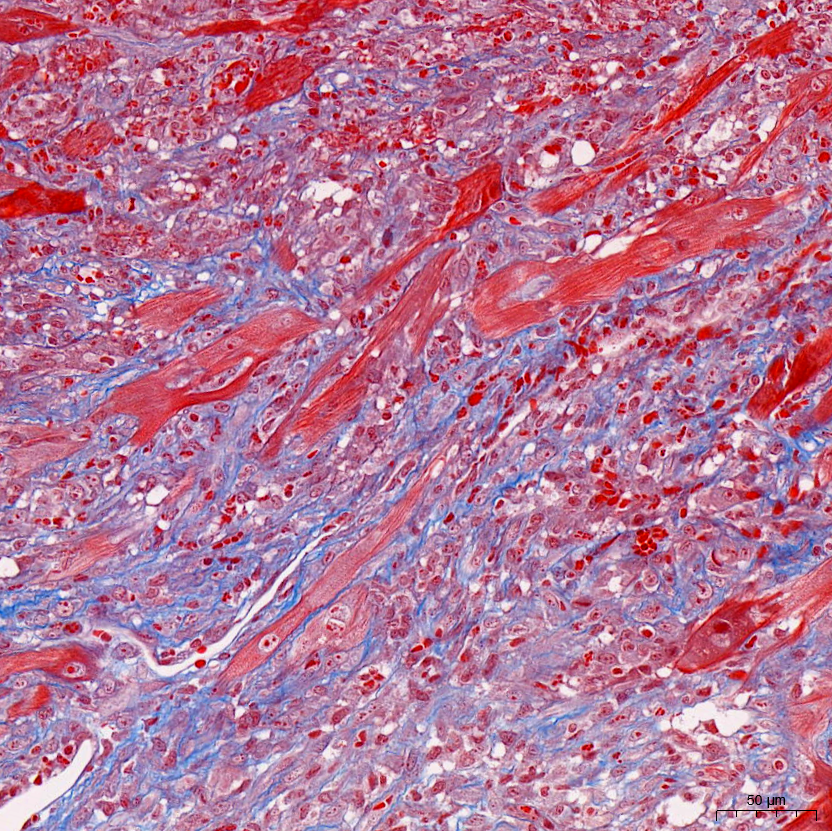

Supplement: Supplementary file 9 [file DataSheet2.ZIP › Raw data-HE, Masson/Masson/inset-Scale bars 100 a╠m/EAM.tif]

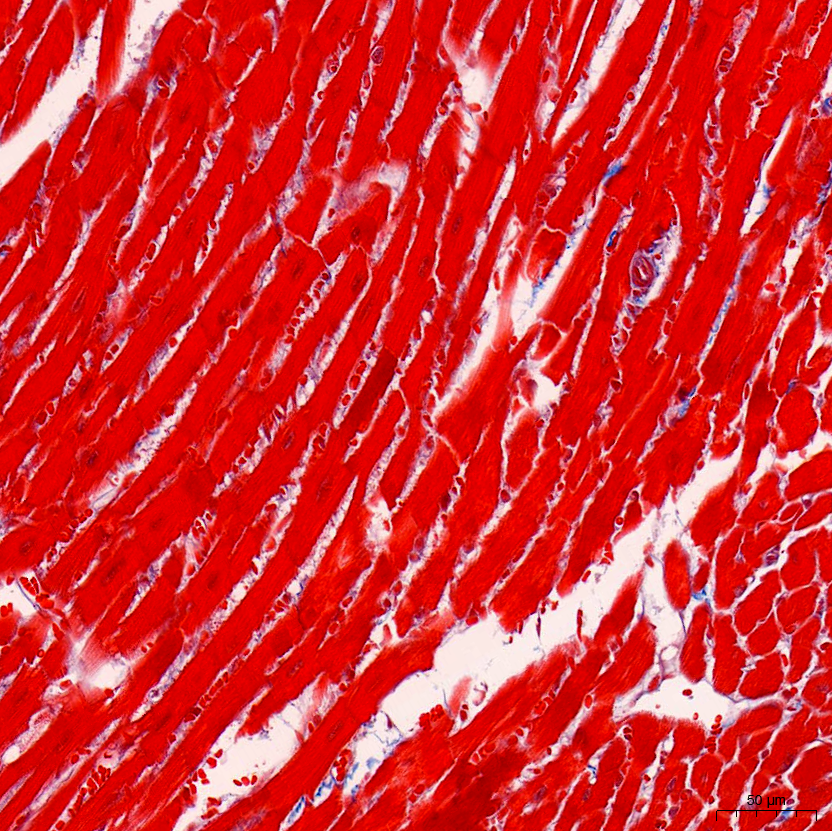

Supplement: Supplementary file 9 [file DataSheet2.ZIP › Raw data-HE, Masson/Masson/inset-Scale bars 100 a╠m/Sham.tif]

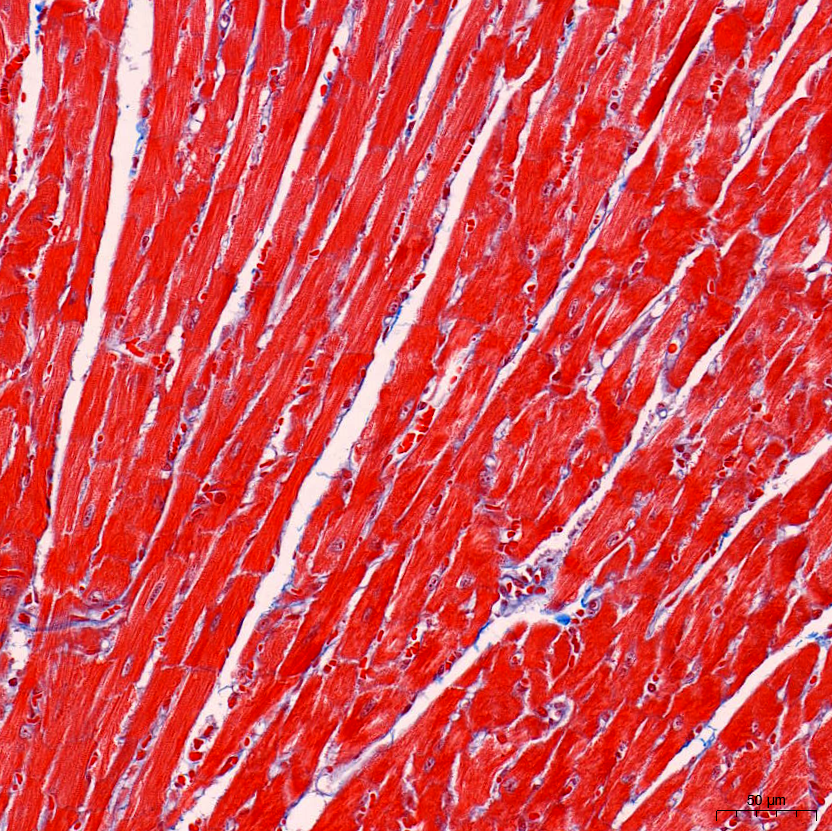

Supplement: Supplementary file 9 [file DataSheet2.ZIP › Raw data-HE, Masson/Masson/inset-Scale bars 100 a╠m/UA.tif]

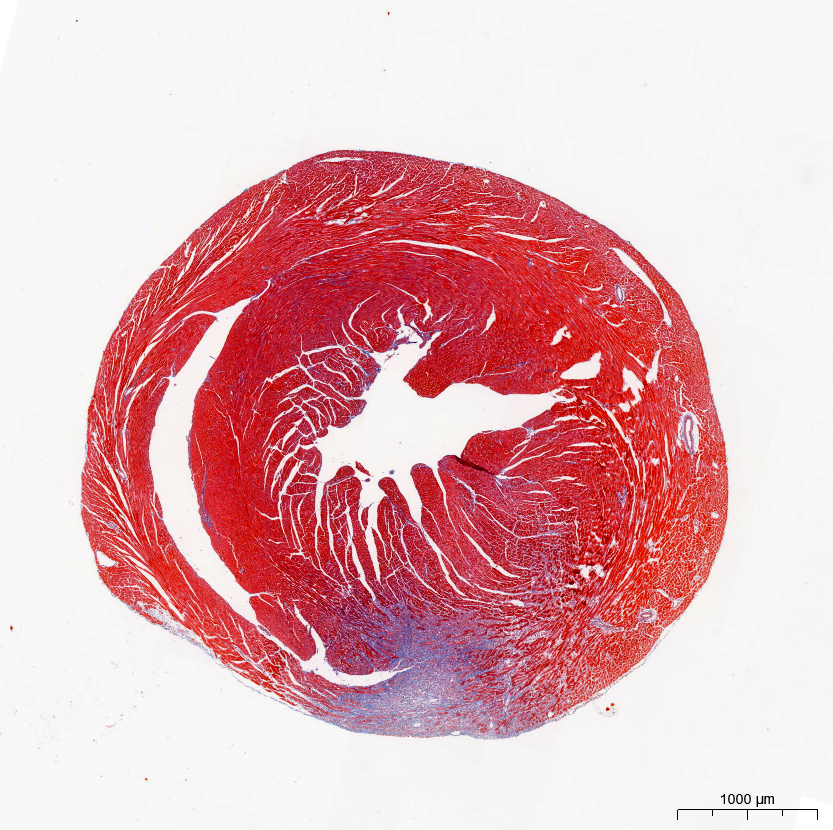

Supplement: Supplementary file 9 [file DataSheet2.ZIP › Raw data-HE, Masson/Masson/left-Scale bars 1mm/EAM.tif]

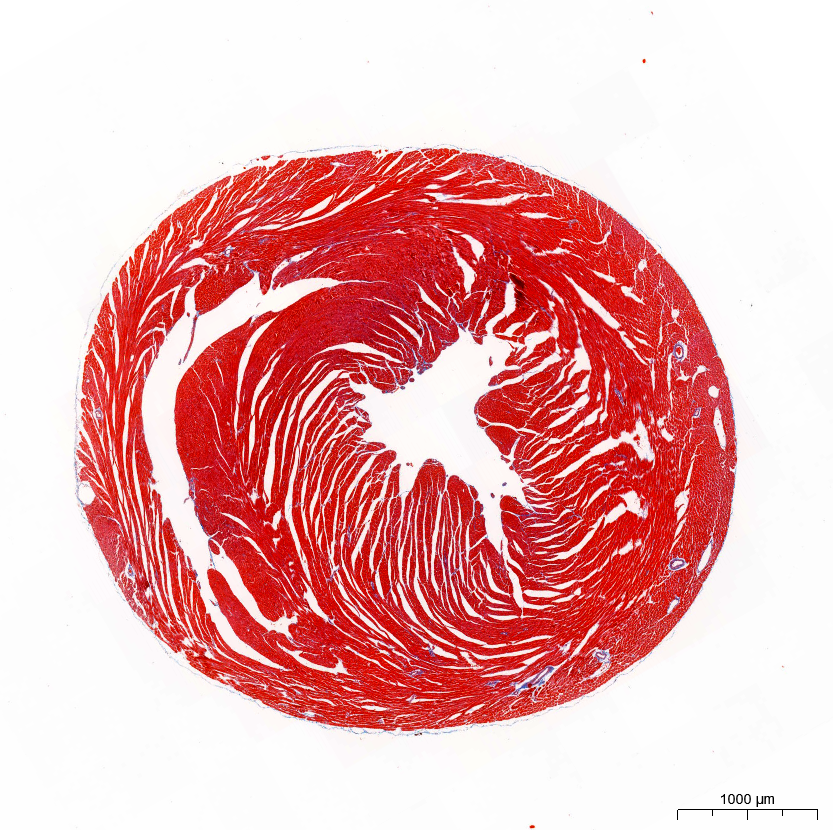

Supplement: Supplementary file 9 [file DataSheet2.ZIP › Raw data-HE, Masson/Masson/left-Scale bars 1mm/Sham.tif]

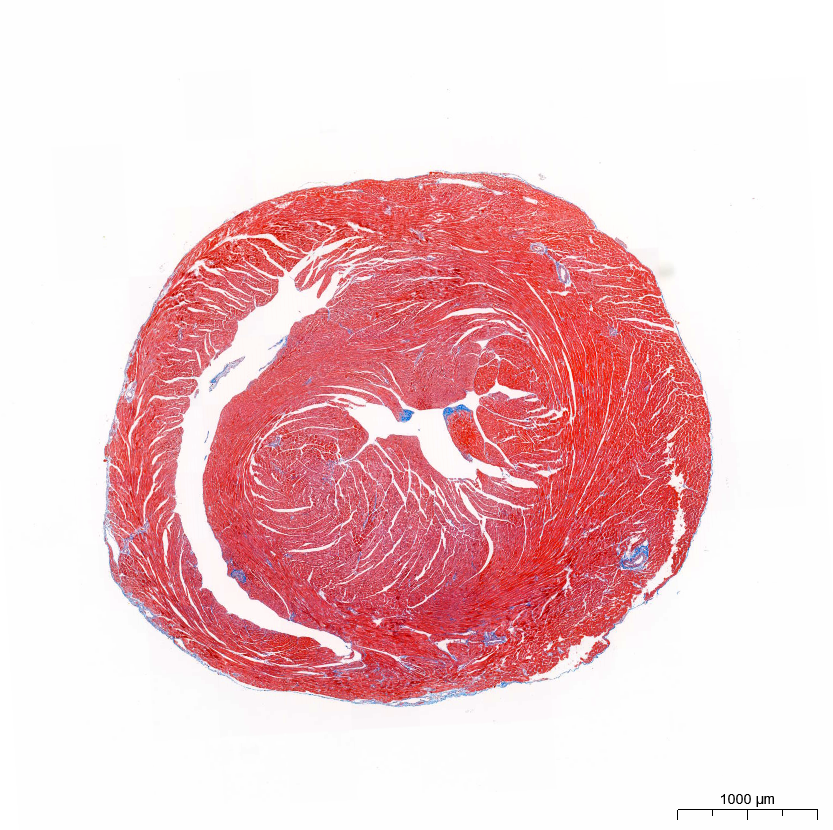

Supplement: Supplementary file 9 [file DataSheet2.ZIP › Raw data-HE, Masson/Masson/left-Scale bars 1mm/UA.tif]
